# Effects of Spore-Displayed p75 Protein from *Lacticaseibacillus rhamnosus* GG on the Transcriptional Response of HT-29 Cells

**DOI:** 10.3390/microorganisms10071276

**Published:** 2022-06-23

**Authors:** Soo-Ji Kang, Min-Joo Kim, Do-Yeong Son, Seok-Seong Kang, Kwang-Won Hong

**Affiliations:** Department of Food Science and Biotechnology, College of Life Science and Biotechnology, Dongguk University, Goyang-si 10326, Korea; susu93@naver.com (S.-J.K.); sindy8292@naver.com (M.-J.K.); sscc03117@naver.com (D.-Y.S.); sskang@dongguk.edu (S.-S.K.)

**Keywords:** p75 protein, postbiotics, spore surface display, RNA sequencing, HT-29 cells

## Abstract

A *Lacticaseibacillus rhamnosus* GG-derived protein, p75, is one of the key molecules exhibiting probiotic activity. However, the molecular mechanism and transcriptional response of p75 in human intestinal epithelial cells are not completely understood. To gain a deeper understanding of its potential probiotic action, this study investigated genome-wide responses of HT-29 cells to stimulation by spore-displayed p75 (CotG-p75) through a transcriptome analysis based on RNA sequencing. Analysis of RNA-seq data showed significant changes of gene expression in HT-29 cells stimulated by CotG-p75 compared to the control. A total of 189 up-regulated and 314 down-regulated genes was found as differentially expressed genes. Gene ontology enrichment analysis revealed that a large number of activated genes was involved in biological processes, such as epithelial cell differentiation, development, and regulation of cell proliferation. A gene–gene interaction network analysis showed that several DEGs, including *AREG*, *EREG*, *HBEGF*, *EPGN*, *FASLG*, *GLI2*, *CDKN1A*, *FOSL1*, *MYC*, *SERPINE1*, *TNFSF10*, *BCL6*, *FLG*, *IVL*, *SPRR1A*, *SPRR1B*, *SPRR3*, and *MUC5AC*, might play a critical role in these biological processes. RNA-seq results for selected genes were verified by reverse transcription-quantitative polymerase chain reaction. Overall, these results provide extensive knowledge about the transcriptional responses of HT-29 cells to stimulation by CotG-p75. This study showed that CotG-p75 can contribute to cell survival and epithelial development in human intestinal epithelial cells.

## 1. Introduction

Probiotics are live microorganisms that can confer several health benefits on the host when administered in appropriate amounts [1]. Several studies have demonstrated that probiotics can promote host health by maintaining intestinal epithelial homeostasis and improving barrier integrity [2]. However, they have some limitations, such as unknown molecular mechanism, strain-specificity, low survival rate, and risks of bacterial infection [3]. Postbiotics are proposed as promising alternatives to overcome these limitations. They are a complex mixture of metabolic products secreted by probiotics, such as soluble proteins, which can enhance the survival and proliferation of intestinal epithelial cells (IECs) [4,5,6,7]. Soluble proteins have several advantages over traditional probiotics, such as known molecular structure, use in pure forms, the specific mechanism of action, and ease of production and storage [3]. They could address concerns associated with the use of “live” probiotics [8]. Further, investigating soluble proteins is crucial for elucidating the mechanisms of probiotics that have not yet been clarified [9,10].

p40 and p75 are major secreted proteins produced by *Lacticaseibacillus rhamnosus* GG (LGG), which is the best-studied probiotic bacterium [11,12]. They were found in numerous *Lacticaseibacillus* species, such as *L. casei*, *L. paracasei*, and *L. rhamnosus* [13,14]. p40 and p75 in *L. casei* BL23 are located at the bacterial cell surface (predominantly at the septum and polar regions) and secreted to the growth medium [13,14]. They are involved in septation and separation of daughter cells [13,14]. Recombinant p40 and p75 proteins bound to mucin, collagen, and intestinal epithelial cells [13]. They stimulated epidermal growth factor receptor (EGFR) phosphorylation in the ex vivo mouse colon organ [13]. They can prevent cytokine-induced apoptosis and promote intestinal epithelial cell homeostasis by stimulating Akt activation in mouse and human intestinal epithelial cells [11,12]. Furthermore, they can attenuate the disruption of epithelial barrier function induced by hydrogen peroxide through protein kinase C (PKC)- and mitogen-activated protein kinase-dependent mechanisms [15]. For p40, its mechanism and regulation of gene expression have been extensively investigated in epithelial intestinal cells and animal models. p40 promotes IgA production via upregulation of a proliferation-inducing ligand (APRIL) and stimulates mucin gene expression and production in LS174T cells and the mouse colonic epithelium [16]. p40 has beneficial effects on prevention or treatment of dextran sulfate sodium (DSS)-induced intestinal epithelial injury and acute colitis and oxazolone-induced T-helper (Th)2 cytokine-driven and 2,4,6-trinitrobenzene sulfonic acid (TNBS)-induced Th1 cytokine-driven chronic colitis in mice [6]. However, the mechanism and transcriptional response of human intestinal epithelial cells to the stimulation by p75 have not been studied. Therefore, studies on p75 may uncover novel probiotic actions not identified in many probiotic strains.

Spore surface display technology is an economical and efficient approach for expressing heterologous proteins on the spore surface [17]. *Bacillus* spores are ideal carriers for oral administration due to their unique properties. *Bacillus* spores have a rigid structure encased in a thick peptidoglycan layer and numerous coat proteins [18]. They can germinate, outgrow, replicate, and re-sporulate in the gastrointestinal (GI) tract [19]. These characteristics help them survive under the harsh environmental conditions of the GI tract [20]. *Bacillus subtilis* spores are used in human and animal food preparations as food additives and prescription or non-prescription products to treat GI disorders [21,22]. Furthermore, the spore formation and purification process are simple and economical [23]. The protein displayed on the spore can be easily purified via simple centrifugation with a high recovery rate [24]. It can display multiple foreign proteins on a single spore. Approximately 10^3^ molecules can be displayed when CotG is used as an anchor protein [25,26]. Although clinical trials in humans have not yet been conducted, this technology has been widely applied for development of oral vaccines and peptides, and its efficacy has been proven in animal models [27,28,29,30].

In a previous study, we have displayed p75 on the spore surface of *B. subtilis* using spore coat protein G (CotG) as an anchor [31]. Evaluating the effects of spore-displayed p75 (CotG-p75), identification of its probiotic action in human intestinal epithelial cells is essential. Furthermore, transcriptional analysis of CotG-p75 may provide clues to elucidate the mechanism involved in the effect of p75 or LGG on human intestinal epithelial cells. Therefore, the aim of this study was to determine the probiotic ability of CotG-p75 in human intestinal epithelial cell line HT-29 through transcriptional analysis using RNA sequencing (RNA-seq). Biological processes and genes involved in important properties of probiotics, such as cell proliferation and cell development, were then characterized through functional analyses of differentially expressed genes (DEGs) and gene ontology (GO) enrichment. Herein, the comprehensive results of gene expression and GO enrichment analyses help to elucidate the potential probiotic action of CotG-p75 in human intestinal epithelial cells.

## 2. Materials and Methods

### 2.1. Spore Preparation

Recombinant *B. subtilis* 168 spores containing pUB19-cotG-p75 were constructed and prepared according to the protocol mentioned in our previous study [31]. Briefly, sporulation of *B. subtilis* 168 wild-type spores and recombinant spores (CotG-p75) was induced with Difco sporulation medium (DSM). After incubation at 37 °C for 62 h, spores were harvested and resuspended in 50 mM sodium phosphate buffer (pH 7.2). Thereafter, spores were treated with lysozyme for 1 h at 4 °C to destroy remaining vegetative cells and then washed 5 times with 50 mM sodium phosphate buffer to remove any residual lysozyme. After centrifugation for 10 min at 4 °C and 5000 rpm, collected spores were resuspended in 1–2 mL of phosphate-buffered saline (PBS). Final spore suspensions were titrated on LB agar to determine spores/mL and adjusted to 10^8^ spores/mL.

### 2.2. Cell Culture and Treatment

Human intestinal epithelial cell line HT-29 was purchased from the American Type Culture Collection (ATCC). These cells were cultured in Dulbecco’s modified Eagle’s medium (DMEM) supplemented with 10% (*v*/*v*) fetal bovine serum (FBS) and 1% (*v*/*v*) penicillin–streptomycin and maintained at a 37 °C in a humidified atmosphere of 95% air and 5% CO_2_. The culture medium was changed 2–3 day. For experiments, HT-29 cells were seeded into 12-well plates at a density of 5 × 10^5^ cells per well. When cells reached 90% confluence, the medium was replaced with 900 μL of serum-free medium. These cells were then treated with 100 μL of 1 × 10^8^ spores/mL of wild-type spores or CotG-p75 and incubated for 3 h. Cells treated with PBS instead of spores were used as controls. After treatment, cells were harvested for subsequent experiments.

### 2.3. Cell Viability Assay

The effect of CotG-p75 on the cell viability of HT-29 cells was determined using a D-Plus CCK Cell Viability Assay Kit (DonginLS, Seoul, Korea) according to the manufacturer’s instructions. Briefly, the cells were seeded at 5 × 10^4^ cells/well in 100 μL of cDMEM in 96 well plates. When HT-29 cells were grown to 70–80% confluency, CotG-p75 was added to final concentrations of 10^5^–10^7^ spores/mL. After 24 h cultivation, the used medium was removed, and a serum-free medium was added. Then, 10 μL of CCK reagent was added per well, and the cells were incubated for 4 h. The optical density was measured at 450 nm using an automated ELISA reader (Thermo Scientific, Waltham, MA, USA). The percentage of living cells was calculated as previously described [32]. The results are presented in Figure S1.

### 2.4. Total RNA Isolation, cDNA Library Construction, and RNA-Sequencing

Total RNAs were extracted from HT-29 cells treated or untreated with spores using TRIzol reagent (Invitrogen, Waltham, MA, USA) following the manufacturer’s protocol. Total RNAs from each sample were isolated in triplicate independently. Isolated total RNA was treated with RNase-free DNaseI to remove potential genomic DNA contaminants. The concentration and quality of total RNA were then determined with a Nanodrop 2000 spectrophotometer (Thermo Fisher Scientific, Waltham, MA, USA). Final total RNA was used for RNA-seq and RT-qPCR.

Sequencing libraries were generated with a TruSeq Stranded mRNA LT Sample Prep Kit (Illumina, San Diego, CA, USA) in accordance with the TruSeq Stranded mRNA Sample Preparation Guide (part # 15031047 Rev. E). Briefly, mRNA was purified from 1 μg of total RNA with poly-T oligo-attached magnetic beads and fragmented into small pieces. These mRNA fragments were transcribed into first-strand complementary DNAs (cDNAs) using reverse transcriptase and random primers, followed by second-strand cDNA synthesis. After purification of double-stranded cDNA, 3′ end adenylation and adapter ligation were performed subsequently. To generate the final cDNA library, products were amplified with PCR, size selected, and purified.

Library preparations and sequencing were carried out by a Macrogen sequencing service (Macrogen Inc., Seoul, Korea). Sequencing was performed on an Illumina NovaSeq 6000 system using a NovaSeq 6000 S4 reagent kit (Illumina, San Diego, CA, USA). Sequencing quality control (QC) was performed using FastQC v 0.11.7 (http://www.bioinformatics.babraham.ac.uk/projects/fastqc/ accessed on 16 February 2022). Trimmomatic 0.38 was used to eliminate 3′ end adaptors and low-quality bases [33]. After trimming, cleaned reads were aligned to the human reference genome (hg19) from the University of California Santa Cruz (UCSC) genome browser using HISAT2 version 2.1.0 software. The sequence data from this study have been deposited into the Sequence Read Archive (http://www.ncbi.nlm.nih.gov/sra accessed on 26 May 2022) under accession number PRJNA792019.

### 2.5. Identification of Differentially Expressed Genes

The mapped reads of each sample were assembled using StringTie (http://ccb.Jhu.edu/software/stringtie/ accessed on 2 March 2022). After the final transcriptome was generated, StringTie and edgeR (http://bioconductor.org/packages/release/bioc/html/edgeR.html accessed on 2 March 2022) were used to estimate the expression levels of all the transcripts. StringTie was used to examine the expression levels by calculating the FPKM (fragments per kilobase of transcripts per million mapped reads). Genes were considered to be differentially expressed between different samples if they met the following criteria: (1) absolute value of fold change (FC) ≥ 2; and (2) raw *p*-value < 0.05. DEGs were investigated for two comparison groups: wild-type treated cells (WT) vs. control (CON) and CotG-p75 (G75) treated cells vs. control (CON).

### 2.6. Hierarchical Clustering

Hierarchical clustering was performed in R Bioconductor using the heatmap.2 function of the gplots package (http://cran.r-project.org/web/packages/gplots/index.html accessed on 14 March 2022). A heatmap was generated by clustering of the 665 genes satisfying the absolute value of FC ≥ 2 and raw *p*-value < 0.05 expressed in all treatment conditions. Calculation of the Z-score was determined as follows: Z = (X−μ)/s, where X is the normalized read counts of a gene for a specific treatment condition, μ is the row mean normalized read counts of a gene across all treatment conditions, and s is the row standard deviation of a gene across all treatment conditions.

### 2.7. Gene Ontology (GO) Enrichment Analysis and Gene–Gene Interaction Network Analysis

To characterize DEGs based on specific biological functions, GO enrichment analysis was carried out using an annotation tool (DAVID; https://david.ncifcrf.gov accessed on 18 March 2022) and visualized with Cytoscape BiNGO (Version 3.2.1) [34]. A hypergeometric test was used as a statistical test. Then, Benjamini and Hochberg false discovery rate (FDR) correction was used for multiple testing correction. GO terms with FDR < 0.05 were considered enriched significantly. The gene–gene network for DEGs was constructed using STRING search tool (https://string-db.org/ accessed on 1 April 2022) with a minimum required interaction score set at >0.4 [35,36].

### 2.8. Reverse Transcription-Quantitative Polymerase Chain Reaction (RT-qPCR) Confirmation

A total of 18 genes related to cell survival and epithelial development (*AREG*, *EREG*, *HBEGF*, *EPGN*, *FASLG*, *GLI2*, *CDKN1A*, *FOSL1*, *MYC*, *SERPINE1*, *TNFSF10*, *BCL6*, *FLG*, *IVL*, *SPRR1A*, *SPRR1B*, *SPRR3*, and *MUC5AC*) were selected and validated by RT-qPCR. Total RNAs (2 μg) were transcribed to cDNAs using random hexamer (Roche, Basel, Switzerland) and M-MLV reverse transcriptase (Promega, Madison, WI, USA) according to the manufacturer’s protocols. RT-qPCR was carried out under the condition at 95 °C for 10 s, followed by 40 cycles of 95 °C for 5 s and 60 °C for 30 s using a SYBR-Green PCR Master Mix kit (Takara, Shiga, Japan) on a CFX Connect Real-Time System (Bio-Rad, Hercules, CA, USA). Gene-specific primer sets for these selected genes were obtained from OriGene technologies (Rockville, MD, USA). They are listed in Table S1. RT-qPCR analysis was performed in triplicate independently. Target genes were normalized against *GAPDH* gene as a reference. Relative expression levels of targeted genes were calculated with the 2^−ΔΔCt^ method [37].

### 2.9. HBEGF and MUC5AC Protein Expression Verification

The level of HBEGF and MUC5AC protein was measured using an HBEGF ELISA kit (Assay Genie, Dublin, Ireland) and MUC5AC ELISA assay kit (Cusabio Biotech, Houston, TX, USA) following the manufacturer’s protocol. HT-29 cells were stimulated by CotG-p75 (10^7^ spores/mL) for 0, 3, 6, and 12 h. The cells treated with no spores or wild-type spores were used as controls. The amount of HBEGF and MUC5AC protein in cell lysates and culture supernatants was normalized to total protein in cell lysates and culture supernatants. The amount of total protein was determined using the BCA protein assay (Pierce, Thermo Scientific, Waltham, MA, USA). The results were expressed as relative expression by normalizing protein expression levels for CotG-p75-treated conditions versus control at all time courses.

### 2.10. Statistical Analysis

All experiments were performed independently in biological triplicates. For RNA-seq data, all reads obtained from libraries were normalized to FPKM. A raw *p*-value < 0.05 and FDR < 0.05 were considered statistically significant in DEG analysis and GO enrichment analysis, respectively.

For RT-qPCR analysis, data were expressed as mean ± standard deviation (SD). Statistical analysis was carried out by a one-way ANOVA and Tukey–Kramer post hoc analysis using GraphPad Prism 5.0 software (GraphPad, San Diego, CA, USA). A *p*-value < 0.05 indicated a statistically significant difference.

## 3. Results

### 3.1. Summary of Transcriptome Sequencing Data

To evaluate the effects of CotG-p75 on human intestinal epithelial cells, transcriptome responses of HT-29 cells to stimulation with wild-type spores or CotG-p75 for 3 h were analyzed through RNA-seq. Prior to in-depth functional analysis, the quality of RNA-seq data was confirmed to remove technical biases in the experiment. The average ratio of clean reads was greater than 95%, and the quality score of all bases was more than 30 in all samples. The GC content of all samples was also very similar to the ideal GC distribution. In addition, the mapping rate was above 95% for all samples, demonstrating the sequencing accuracy. Based on the quality analysis results of RNA-seq data, the sequencing quality was sufficient for the following research. The quality control results for all samples are provided in Table S2.

### 3.2. Differential Gene Expression in HT-29 Cells Stimulated with CotG-p75

**Figure 1:**
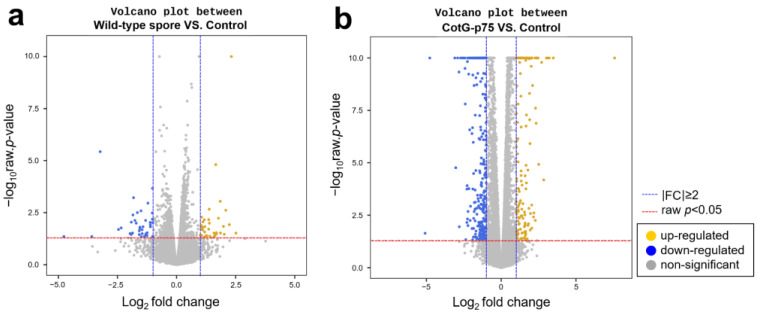
Differentially expressed gene (DEG) distribution trends in HT-29 cells in two comparison groups: wild-type spore-stimulated cells versus control cells (**a**), and CotG-p75-stimulated cells versus control cells (**b**). Blue vertical dashed lines indicate |Log2 (fold change)| = 1. Red horizontal dashed lines indicate the preset statistical threshold corresponding to an adjusted *p*-value < 0.05. Yellow and blue dots represent up-regulated and down-regulated DEGs, respectively. Gray dots represent non-differentially expressed genes.

To identify changes in gene expression that might be related to the probiotic action in human intestinal epithelial cells, DEGs were monitored by RNA-seq. After stimulating HT-29 cells with wild-type spore or CotG-p75 for 3 h, gene expression profiles were compared to those of the control. Genes satisfying the absolute value of FC ≥ 2 and raw *p*-value < 0.05 were considered to be statistically significant. The volcano plot in Figure 1 shows DEGs for two comparison groups: wild-type spore-stimulated cells vs. control cells (Figure 1a) and CotG-p75-stimulated cells vs. control cells (Figure 1b). Yellow and blue dots indicate significantly up- and down-regulated DEGs, respectively. A total of 74 (35 up-regulated and 39 down-regulated) and 503 (189 up-regulated and 314 down-regulated) DEGs was found in cells stimulated by wild-type spores and CotG-p75, respectively.

**Figure 2:**
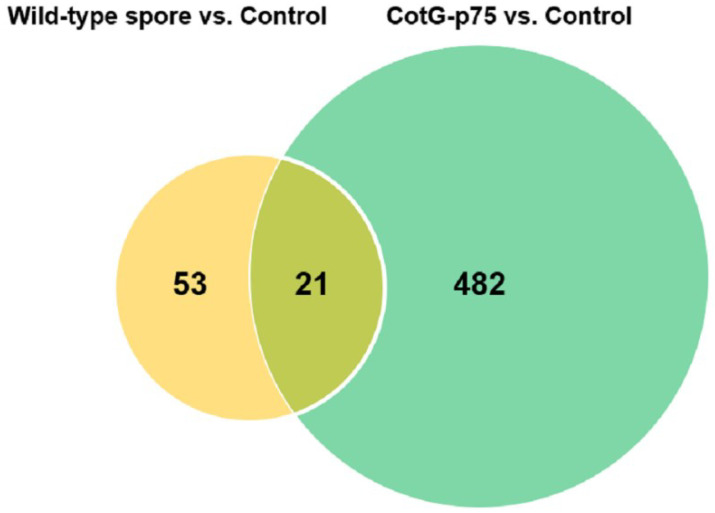
Venn diagram of differentially expressed genes (DEGs) in HT-29 cells stimulated with wild-type spore or CotG-p75 compared to control. Overlapping portions of different circles represent the number of DEGs that are common, and non-overlapping portions represent the number of DEGs that are unique to the comparison group.

**Figure 3:**
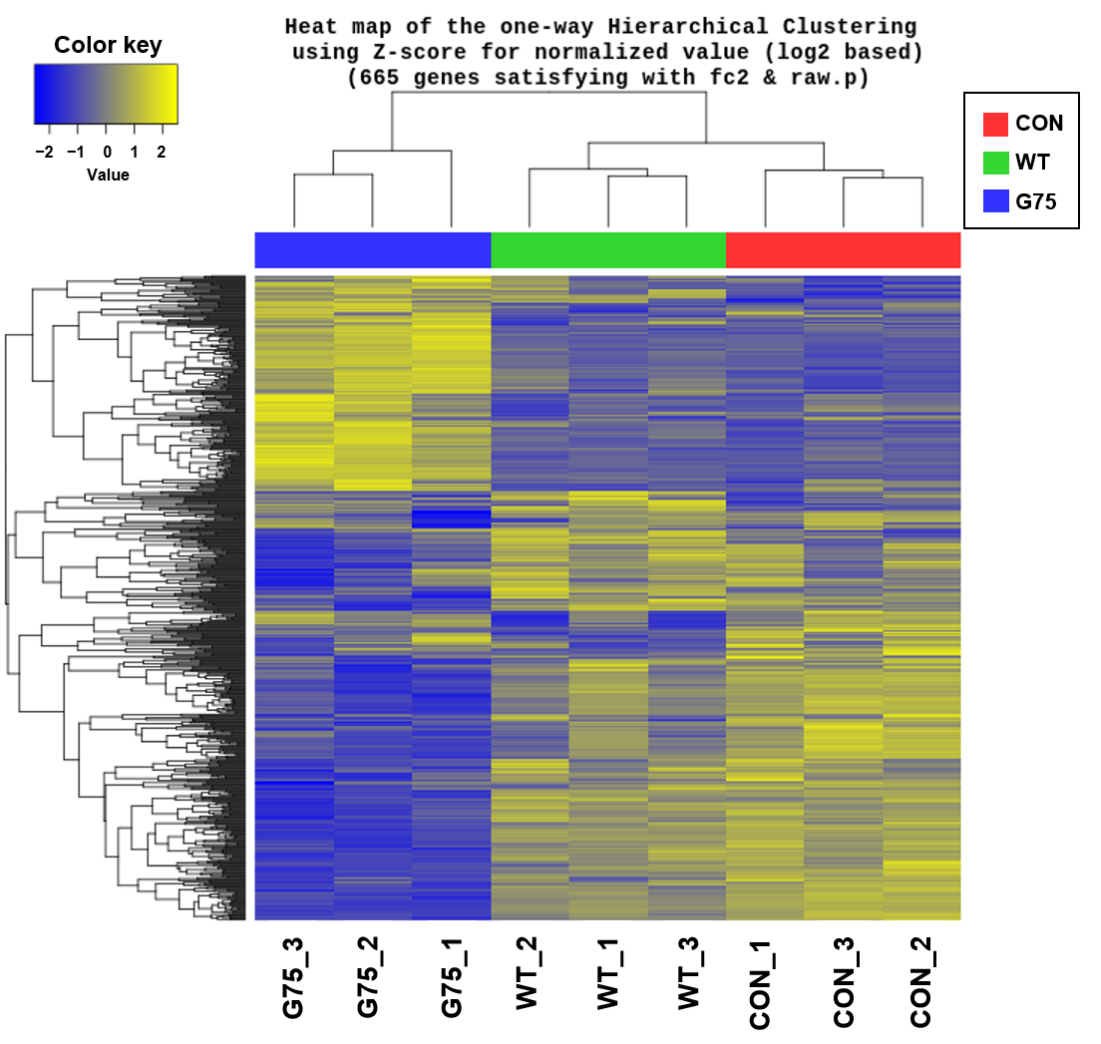
Hierarchical clustering of differentially expressed genes (DEGs) in HT-29 cells stimulated by wild-type spores (WT) or CotG-p75 (G75) compared to control (CON). Each row represents one of the common genes, and each column represents each sample. The color scale means gene expression standard deviations from the mean represented as Z-score. Yellow indicates up-regulation, and blue indicates down-regulation.

A Venn diagram showed shared DEGs between wild-type spore-treated cells and CotG-p75-treated cells. As shown in Figure 2, wild-type spores and CotG-p75 affected the expression levels of 74 and 503 genes, respectively. Of these genes, 21 were commonly affect by both wild-type spores and CotG-p75. This result shows that gene expression in HT-29 cells is affected more by CotG-p75 than by wild-type spores. Furthermore, hierarchical clustering was carried out to compare gene expression patterns among control, wild-type spore-treated, and CotG-p75-treated cells. The results were visualized as a heat-map (Figure 3). The heatmap showed a distinct difference in gene expression patterns between CotG-p75-treated cells and untreated control cells. The overall gene expression profile of HT-29 cells stimulated with CotG-p75 was significantly different from that of wild-type spore-treated cells or control cells. This indicated that CotG-p75 mediated transcriptome alterations in HT-29 cells. DEG analysis results for all investigated comparison groups, namely, wild-type spore treated cells vs. control, CotG-p75 treated cells vs. control, and CotG-p75 treated cells vs. wild-type spore treated cells, are provided in Tables S3–S5, respectively.

### 3.3. Gene Ontology (GO) Enrichment Analysis

**Figure 4:**
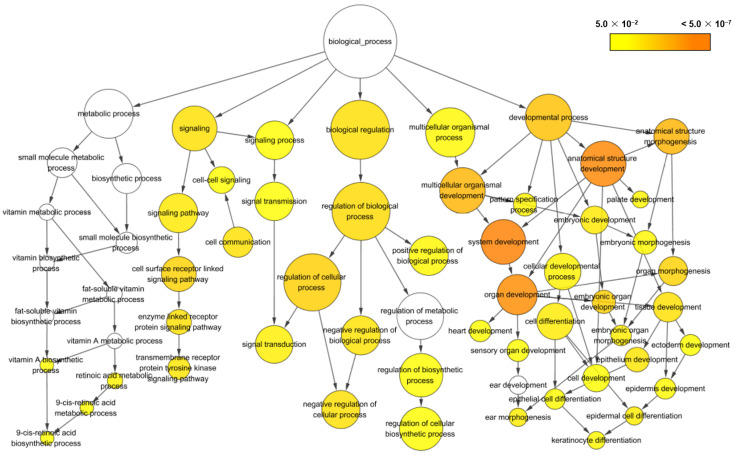
Gene ontology (GO) networks of differentially expressed genes generated by Cytoscape BiNGO. All significantly enriched GO terms in biological processes are presented. Circle size represents GO hierarchy. Yellow shade represents enrichment level. The threshold of hypergeometric distribution of the functional annotation was set at corrected *p*-value < 0.01.

To investigate the biological functions of genes in HT-29 cells affected by wild-type spores and CotG-p75, a GO enrichment analysis of the filtered DEGs was conducted using the Cytoscape BiNGO annotation tool. GO terms with FDR < 0.05 were considered enriched significantly. Analysis of all 503 DEGs found in CotG-p75-stimulated cells revealed 135 significantly enriched GO terms, which were separated into the main categories: biological process (124), molecular function (9), and cellular component (2). Among them, the 27 GO terms satisfying an FDR ≤ 0.01 are presented in Table 1. The relationship among GO terms as a network is shown in Figure 4. The DEGs enriched in biological process ontology were mainly related to the developmental process (GO:0032502), multicellular organismal process (GO:0032501), biological regulation (GO:0065007), signaling (GO:0023052), signaling process (GO:0023046), and the metabolic process (GO:0008152). Most DEGs were involved in the developmental process and associated with cell development and differentiation in the multicellular organismal process. In addition, CotG-p75 influenced signaling processes, including signal transduction and signal pathways. Some DEGs also participated in the vitamin A biosynthetic processes and the retinoic acid metabolic process. However, we could find only a few minor enriched GO terms in the wild-type spore-treated group (Figure S2). Our results suggested that treatment with CotG-p75 mainly affects biological processes of HT-29 cells, especially cell development, cell differentiation, and cell survival. GO enrichment analysis results for all GO terms and related genes are provided in Table S6 for wild-type-stimulated cells and Table S7 for CotG-p75 stimulated cells.

### 3.4. Identification of DEGs Related to Cell Survival and Development

**Figure 5:**
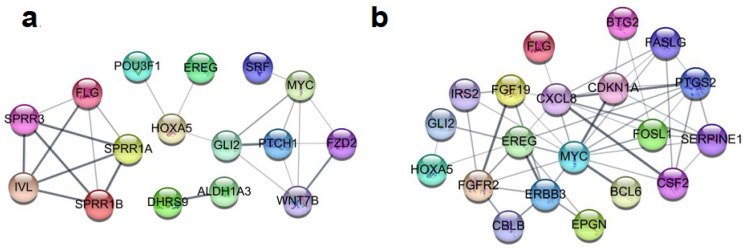
Networks of gene–gene interactions involved in epithelial cell differentiation and development (**a**) and cell proliferation (**b**). These networks were visualized with a search tool for the retrieval of interacting genes (STRING) and constructed with a required confidence score of 0.4. Nodes and lines represent genes and interactions between genes, respectively.

The GO enrichment analysis revealed that most DEGs were related to cell survival and cell development in HT-29 cells. As cell proliferation and development are important factors of probiotic action, we focused on DEGs associated with these properties. These DEGs were clustered into two major categories: “epithelial cell differentiation and development” (16 genes), and “cell proliferation” (20 genes). The genes included in these categories are listed in Table 2, and their interactions are presented in Figure 5. Specifically, epithelial barrier- and tissue remodeling-related genes, such as *FLG*, *IVL*, *SPPR1A*, *SPPR1B*, and *SPRR3*, were up-regulated and involved in epithelial cell differentiation and development processes. Moreover, genes associated with cell survival and proliferation were up-regulated, such as *MYC*, *EREG*, *FASLG*, *GLI2*, *EPGN*, *FOSL1*, and *SERPINE1*. On the contrary, pro-apoptotic genes, such as *BCL6*, *TNFSF10*, and *BTG2*, were down-regulated. Among these genes, seven major genes (*FASLG*, *CDKN1A*, *CSF2*, *SERPINE1*, *EREG*, *CXCL8*, and *MYC*) showed the highest connectivity in the network. Thus, they might play a pivotal role in cell survival and development.

### 3.5. Validation of RNA-seq Data by RT-qPCR

**Figure 6:**
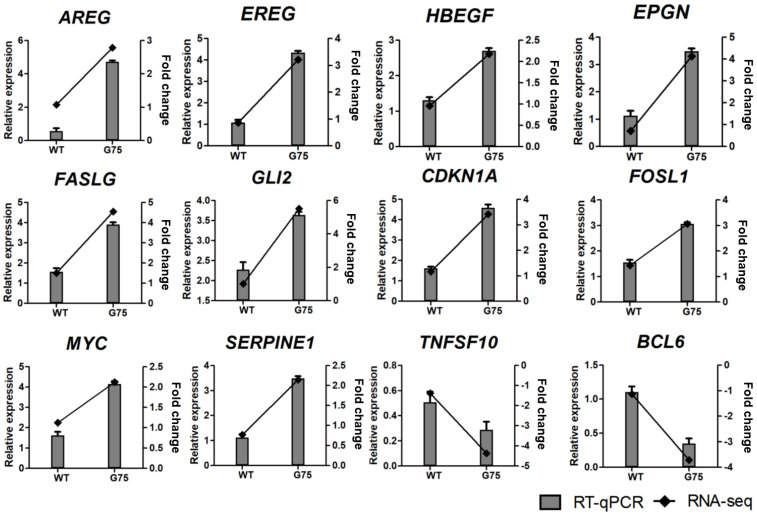
Real-time quantitative PCR validation of RNA-seq data for genes involved in cell survival and proliferation from HT-29 stimulated by CotG-p75. The right and left y-axis represent the fold change of RNA-seq data and relative expression of RT-qPCR data, respectively. The relative mRNA expression was compared with spore-untreated control. RT-qPCR analysis for mRNA expression of selected genes was normalized against *GAPDH*. RT-qPCR data are presented as mean ± standard deviation (*n* = 3).

**Figure 7:**
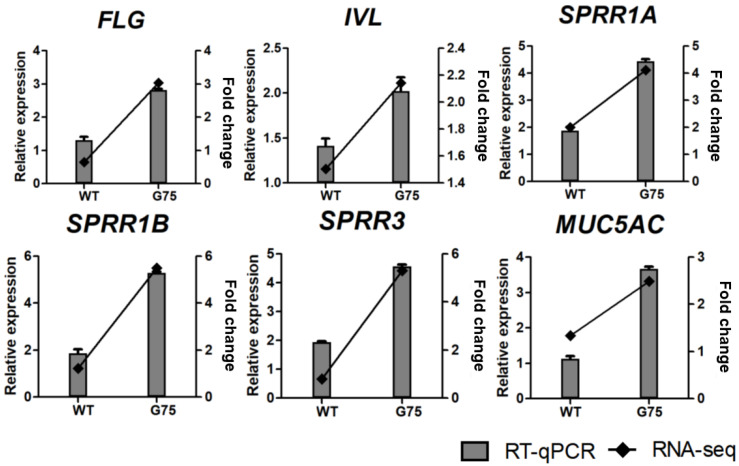
Real-time quantitative PCR validation of RNA-seq data for genes involved in epithelium development and differentiation from HT-29 stimulated by CotG-p75. The right and left y-axis represent the fold change of RNA-seq data and relative expression of RT-qPCR data, respectively. The relative mRNA expression was compared with spore-untreated control. RT-qPCR analysis for mRNA expression of selected genes was normalized against *GAPDH*. RT-qPCR data are presented as mean ± standard deviation (*n* = 3).

To validate the results of DEGs identified by RNA-seq, we performed RT-qPCR for 18 selected genes, including 12 genes related to cell survival and proliferation (*AREG*, *EREG*, *HBEGF*, *EPGN*, *FASLG*, *GLI2*, *CDKN1A*, *FOSL1*, *MYC*, *SERPINE1*, *TNFSF10*, and *BCL6*) and 6 genes related to epithelial development and differentiation (*FLG*, *IVL*, *SPRR1A*, *SPRR1B*, *SPRR3*, and *MUC5AC*). RT-qPCR analysis showed that the tendency of DEG expression was consistent with the RNA-seq results (Figure 6 and Figure 7). The mRNA levels of selected genes were dose-dependently increased or decreased by CotG-p75 (Figures S3 and S4). Therefore, the accuracy and reliability of the RNA-seq data were confirmed by RT-qPCR.

### 3.6. Effect of CotG-p75 on Heparin-Binding Epidermal Growth Factor (HBEGF) and MUC5AC Protein Expression

**Figure 8:**
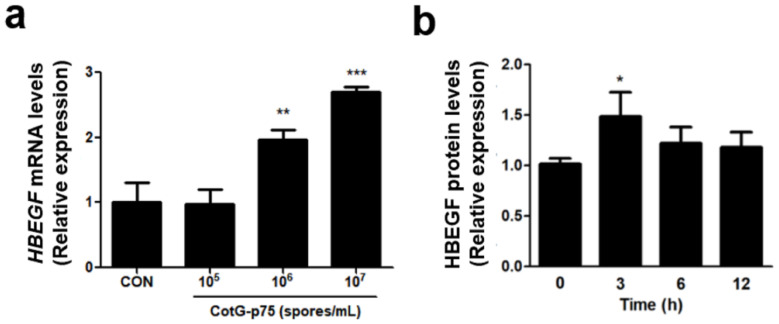
Effect of CotG-p75 on HBEGF mRNA expression (**a**) and HBEGF protein production (**b**). In panel (**a**), cells were treated with various concentrations of CotG-p75 (10^5^ to 10^7^ spores/mL) for 3 h. In panel (**b**), cells were treated with CotG-p75 (10^7^ spores/mL) during various times (0, 3, 6, and 12 h). The mRNA expression levels and protein production levels in treated groups were compared with the control group. All data are expressed as mean ± standard deviation (*n* = 3). Asterisks (*) indicate a significance difference from the control (* *p* < 0.05, ** *p* < 0.01, *** *p* < 0.001).

**Figure 9:**
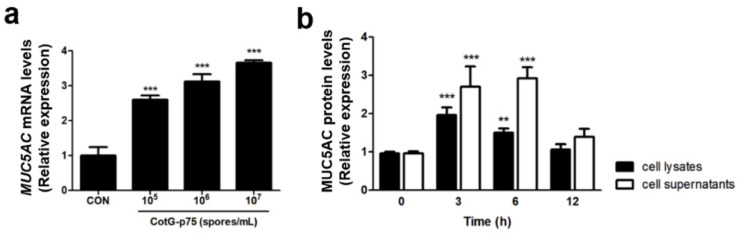
Effect of CotG-p75 on MUC5AC mRNA expression (**a**) and MUC5AC protein production (**b**). In panel (**a**), cells were treated with various concentrations of CotG-p75 (10^5^ to 10^7^ spores/mL) for 3 h. In panel (**b**), cells were treated with CotG-p75 (10^7^ spores/mL) during various times (0, 3, 6, and 12 h). The mRNA expression levels and protein production levels in treated groups were compared with the control group. All data are expressed as mean ± standard deviation (*n* = 3). Asterisks (*) indicate a significance difference from the control (** *p* < 0.01, *** *p* < 0.001).

HBEGF and MUC5AC genes associated with the EGFR signaling pathway and mucin production were selected for RNA-seq analysis to verify the DEGs results. The mRNA expression of HBEGF was increased dose-dependently in cells treated with CotG-p75 (Figure 8a). The protein expression of HBEGF was increased significantly by CotG-p75 (Figure 8b). The maximum induction was reached at 3 h and was maintained until 6 h. CotG-p75 induced the expression of MUC5AC mRNA in a dose-dependent manner (Figure 9a) and induced the expression of MUC5AC protein in HT-29 cells (Figure 9b). The highest protein level of MUC5AC in cell lysates was observed at 3 h. The maximal protein level of MUC5AC in cell supernatants was observed at 6 h. However, the MUC5AC protein level returned to baseline after CotG-p75 treatment for 12 h. These data indicated that CotG-p75 induced HBEGF release and mucin production.

## 4. Discussion

In our previous study, we displayed p75 protein on the surface of *B. subtilis* spores using CotG as an anchor [31]. In the present study, we investigated the effects of CotG-p75 on transcriptional responses of the human intestinal epithelial cell line HT-29. We focused on biological processes involved in the probiotic properties, especially cell survival and development. Our results demonstrated that CotG-p75 affected genes involved in cell survival and development. We also discovered genes involved in these processes through DEG and GO enrichment analyses.

An interesting finding from GO enrichment analysis was the prevalence of GO terms related to cell developmental processes, especially cell proliferation and differentiation. Notably, genes involved in the tumor necrosis factor (TNF) superfamily were regulated by CotG-p75. This superfamily regulates a variety of cellular functions, including immune response, inflammation, proliferation, differentiation, and apoptosis [38]. FASLG and TNFSF10 (also known as TRAIL) are reported to contribute to apoptosis induction as ligands of the TNF pathway [39,40]. Moreover, TNFSF10 has a pro-inflammatory effect. Its up-regulation has been observed in intestinal inflammatory conditions, such as inflammatory bowel disease (IBD) [41]. TNFRSF1B is a receptor of the TNF superfamily and a critical signaling protein in the immune system [42,43]. In the present work, CotG-p75 up-regulated the expression of *FASLG* and *TNFRSF1B* but down-regulated the expression of *TNFSF10*. It also up-regulated *CCL20* and *CSF2* known to play important roles in leukocyte recruitment and activation of downstream signals of the TNF signaling pathway (Figure S5). Additionally, CotG-p75 down-regulated pro-apoptotic and antiproliferation genes such as *BCL6*, *TNFSF10*, and *BTG2*. By contrast, CotG-p75 up-regulated anti-apoptotic and pro-proliferation genes in the Hippo signaling pathway, such as *GLI2*, *AREG*, *SERPINE1*, *NKD2*, and *MYC* (Figure S6). These alterations in gene expression indicated that CotG-p75 could modulate cell apoptosis, proliferation, and immune responses in human intestinal epithelial cells.

Notably, genes encoding EGFR ligands, such as *HBEGF*, *EREG*, *AREG*, and *EPGN*, were all up-regulated in CotG-p75-stimulated cells. The EGFR signaling pathway is critical to maintaining epithelial homeostasis by promoting cell proliferation, differentiation, migration, and survival [44,45]. EGFR activation also contributes to ameliorating chronic inflammation, limiting colitis-associated tumorigenesis [46]. It has also been reported to regulate mucin production [47]. Similar to our results, many studies have shown that p40, another secreted protein of LGG, modulates intestinal epithelial cell homeostasis through HBEGF release, leading to EGFR activation [6,16,48]. p75 activates Akt, a downstream target of the EGFR signaling pathway, in intestinal epithelial cells [15]. Therefore, we could speculate that CotG-p75 may affect EGFR activation by stimulating the EGFR ligand genes, thereby contributing to cell proliferation.

Fortification of the epithelial barrier is crucial for gut health because the major function of the epithelial barrier is to prevent the invasion of microorganisms and their products [49]. We found that epithelial barrier-associated genes, such as *FLG*, *IVL*, *SPRR1A*, *SPRR1B*, and *SPRR3*, were up-regulated in CotG-p75-stimulated cells and were involved in epithelial cell development and differentiation processes. These barrier-related molecules belong to the epidermal differentiation complex and are essential for maintaining appropriate barrier function [50]. FLG (filaggrin) and IVL (involucrin) are major proteins that affect the skin barrier [51,52]. A loss-of-function mutation in these proteins is closely associated with atopic dermatitis [52,53]. Some studies have suggested that FLG might affect the barrier function of the GI tract and alter the risk of various GI disorders, such as IBD [54].

The small proline-rich protein (SPRR) family is a cornified cell envelope precursor. It is associated with epithelial proliferation and barrier function [55]. SPRRs can lead to reduced hydration, antioxidant defense, wound healing, drug permeability, and resistance to infection [56]. Many previous studies have provided evidence that SPRR2 plays an important role in barrier development and function in the intestinal epithelium. They showed that the SPRR2 participates in fortifying the intestinal epithelial barrier in response to bacterial infection [57,58,59]. A study suggested that SPRR2 may be crucial for cross-bridge formation in the serosa or between newly produced enterocytes [60].

Up-regulation of polymeric gel-forming mucin MUC5AC, encoded by the *MUC5AC* gene, and promoted mucin production were observed in CotG-p75-treated cells. Mucins are key components in maintaining intestinal epithelial homeostasis and improving epithelial barrier integrity [61,62]. The mucus layer prevents the adhesion and binding of pathogenic microorganisms, thereby preventing the invasion of intestinal pathogens [63]. LGG induces mucin gene expression and mucin production in human colon cells, including LS174T, Caco-2, and HT-29 [64,65,66]. p40 induces mucin gene expression and mucin production in LS174T cells and mouse colonic epithelium in an EFGR-dependent manner [15]. Moreover, EGFR signaling regulates mucin production [47]. These results are consistent with our findings that CotG-p75 increases gene expression of EGFR ligands. Therefore, these results suggested that CotG-p75 might play a critical role in protecting the intestinal epithelium and maintaining barrier homeostasis by promoting mucin production. Overall, our results suggested that CotG-p75 might participate in cell proliferation and differentiation by stimulating these epithelial barrier fortification-related molecules in human intestinal epithelial cells.

In conclusion, transcriptome analysis of HT-29 cells stimulated with CotG-p75 suggested that several genes activated by CotG-p75 are involved in biological processes, especially cell survival and epithelial development. Our findings provide novel insight into the transcriptional response of human intestinal epithelial cells to stimulation by CotG-p75. Since we identified transcriptional responses only in the HT-29 cell line, we cannot predict whether CotG-p75 will act in the same way in vivo. Therefore, further studies are needed to investigate whether these responses can activate related factors in the signaling pathway or induce differential phenotype expression in other human intestinal epithelial cell lines or animal models. The transcriptomic-based approach of this study may help determine the targets of several signal transduction pathways for in vivo studies. These results indicate that CotG-p75 can contribute to the improvement of survival and development of human intestinal epithelial cells.

## Figures and Tables

**Table 1:** Gene ontology (GO) enrichment analysis of differentially expressed genes in HT-29 stimulated with CotG-p75.

GO ID	GO Description	Adjusted *p*-Value ^a^	Cluster Frequency ^b^	Total Frequency ^c^
GO:0048731	system development	2.04 × 10^−8^	103/387 (26.6%)	2421/17,777 (13.6%)
GO:0048856	anatomical structure development	2.14 × 10^−7^	106/387 (27.3%)	2655/17,777 (14.9%)
GO:0048513	organ development	3.23 × 10^−7^	80/387 (20.6%)	1791/17,777 (10.0%)
GO:0009653	anatomical structure morphogenesis	1.12 × 10^−5^	58/387 (14.9%)	1217/17,777 (6.8%)
GO:0007275	multicellular organismal development	1.16 × 10^−5^	108/387 (27.9%)	2970/17,777 (16.7%)
GO:0032502	developmental process	3.53 × 10^−5^	113/387 (29.1%)	3233/17,777 (18.1%)
GO:0048568	embryonic organ development	1.27 × 10^−4^	19/387 (4.9%)	215/17,777 (1.2%)
GO:0050794	regulation of cellular process	1.35 × 10^−4^	183/387 (47.2%)	6218/17,777 (34.9%)
GO:0007166	cell surface receptor linked signaling pathway	1.55 × 10^−4^	56/387 (14.4%)	1279/17,777 (7.1%)
GO:0009887	organ morphogenesis	1.62 × 10^−4^	35/387 (9.0%)	634/17,777 (3.5%)
GO:0050789	regulation of biological process	3.76 × 10^−4^	188/387 (48.5%)	6548/17,777 (36.8%)
GO:0065007	biological regulation	5.21 × 10^−4^	196/387 (50.6%)	6937/17,777 (39.0%)
GO:0007167	enzyme linked receptor protein signaling pathway	6.06 × 10^−4^	23/387 (5.9%)	346/17,777 (1.9%)
GO:0048523	negative regulation of cellular process	6.13 × 10^−4^	70/387 (18.0%)	1844/17,777 (10.3%)
GO:0023052	signaling	6.46 × 10^−4^	104/387 (26.8%)	3130/17,777 (17.6%)
GO:0009888	tissue development	6.46 × 10^−4^	37/387 (9.5%)	748/17,777 (4.2%)
GO:0007154	cell communication	6.58 × 10^−4^	39/387 (10.0%)	812/17,777 (4.5%)
GO:0048519	negative regulation of biological process	8.37 × 10^−4^	74/387 (19.1%)	2019/17,777 (11.3%)
GO:0060429	epithelium development	8.69 × 10^−4^	22/387 (5.6%)	336/17,777 (1.8%)
GO:0007169	transmembrane receptor protein tyrosine kinase signaling pathway	8.69 × 10^−4^	60/387 (15.5%)	1526/17,777 (8.5%)
GO:0009790	embryonic development	1.06 × 10^−3^	17/387 (4.3%)	219/17,777 (1.2%)
GO:0023033	signaling pathway	1.39 × 10^−3^	31/387 (8.0%)	602/17,777 (3.3%)
GO:0030154	cell differentiation	1.39 × 10^−3^	75/387 (19.3%)	2099/17,777 (11.8%)
GO:0048562	embryonic organ morphogenesis	1.47 × 10^−3^	63/387 (16.2%)	1668/17,777 (9.3%)
GO:0042471	ear morphogenesis	1.64 × 10^−3^	13/387 (3.3%)	140/17,777 (0.7%)
GO:0009913	epidermal cell differentiation	1.64 × 10^−3^	9/387 (2.3%)	66/17,777 (0.3%)
GO:0007165	signal transduction	2.02 × 10^−3^	9/387 (2.3%)	68/17,777 (0.3%)
GO:0048869	cellular developmental process	2.09 × 10^−3^	68/387 (17.5%)	1877/17,777 (10.5%)
GO:0007389	pattern specification process	2.78 × 10^−3^	63/387 (16.2%)	1714/17,777 (9.6%)
GO:0042573	retinoic acid metabolic process	2.78 × 10^−3^	60/387 (15.5%)	1607/17,777 (9.0%)
GO:0060021	palate development	3.25 × 10^−3^	18/387 (4.6%)	271/17,777 (1.5%)
GO:0048598	embryonic morphogenesis	3.47 × 10^−3^	5/387 (1.2%)	18/17,777 (0.1%)
GO:0048518	positive regulation of biological process	3.75 × 10^−3^	7/387 (1.8%)	43/17,777 (0.2%)
GO:0032501	multicellular organismal process	5.09 × 10^−3^	20/387 (5.1%)	336/17,777 (1.8%)
GO:0008544	epidermis development	5.09 × 10^−3^	75/387 (19.3%)	2207/17,777 (12.4%)
GO:0030216	keratinocyte differentiation	5.50 × 10^−3^	129/387 (33.3%)	4374/17,777 (24.6%)
GO:0030855	epithelial cell differentiation	5.50 × 10^−3^	14/387 (3.6%)	186/17,777 (1.0%)
GO:0031326	regulation of cellular biosynthetic process	5.78 × 10^−3^	8/387 (2.0%)	63/17,777 (0.3%)
GO:0007267	cell–cell signaling	6.60 × 10^−3^	17/387 (4.3%)	265/17,777 (1.4%)
GO:0042904	9-cis-retinoic acid biosynthetic process	7.01 × 10^−3^	13/387 (3.3%)	168/17,777 (0.9%)
GO:0042905	9-cis-retinoic acid metabolic process	7.10 × 10^−3^	95/387 (24.5%)	3021/17,777 (16.9%)
GO:0035238	vitamin A biosynthetic process	7.10 × 10^−3^	8/387 (2.0%)	66/17,777 (0.3%)
GO:0007507	heart development	7.10 × 10^−3^	28/387 (7.2%)	587/17,777 (3.3%)
GO:0007423	sensory organ development	7.10 × 10^−3^	3/387 (0.7%)	5/17,777 (0.0%)
GO:0009889	regulation of biosynthetic process	7.10 × 10^−3^	3/387 (0.7%)	5/17,777 (0.0%)
GO:0023046	signaling process	7.10 × 10^−3^	3/387 (0.7%)	5/17,777 (0.0%)
GO:0023060	signal transmission	7.10 × 10^−3^	3/387 (0.7%)	5/17,777 (0.0%)
GO:0048468	cell development	7.10 × 10^−3^	3/387 (0.7%)	5/17,777 (0.0%)
GO:0007398	ectoderm development	7.40 × 10^−3^	15/387 (3.8%)	221/17,777 (1.2%)

^a^*p*-value in hypergeometric test after correction. ^b^ The denominator represents the total number of genes with GO annotation, and the numerator represents the number of genes belonging to each GO term. ^c^ The denominator represents the number of reference genes with GO annotation, and the numerator represents the number of references genes annotated in the listed GO term.

**Table 2:** List of genes involved in epithelial cell differentiation and development and cell proliferation in HT-29 cells stimulated with CotG-p75.

Gene Symbol	Gene Name	Fold Change	*p*-Value
Epithelial Cell Differentiation and Development			
*ALDH1A3*	aldehyde dehydrogenase 1 family member A3	8.154	0.000
*GLI2*	GLI family zinc finger 2	5.524	0.000
*SPRR1B*	small proline rich protein 1B	5.493	0.000
*DHRS9*	dehydrogenase/reductase 9	5.330	0.000
*SPRR3*	small proline rich protein 3	5.304	0.000
*SPRR1A*	small proline rich protein 1A	4.118	0.001
*EREG*	epiregulin	3.221	0.000
*FLG*	filaggrin	3.037	0.031
*IVL*	involucrin	2.141	0.000
*WNT7B*	Wnt family member 7B	2.134	0.000
*SRF*	serum response factor	2.124	0.046
*MYC*	MYC proto-oncogene, bHLH transcription factor	2.120	0.000
*HOXA5*	homeobox A5	−2.043	0.000
*FZD2*	frizzled class receptor 2	−2.697	0.022
*PTCH1*	patched 1	−2.987	0.000
*POU3F1*	POU class 3 homeobox 1	−3.769	0.000
Cell proliferation			
*GLI2*	GLI family zinc finger 2	5.524	0.000
*FASLG*	Fas ligand	4.559	0.002
*EPGN*	epithelial mitogen	4.132	0.013
*CDKN1A*	cyclin dependent kinase inhibitor 1A	3.420	0.000
*CSF2*	colony stimulating factor 2	3.225	0.000
*EREG*	epiregulin	3.221	0.000
*FOSL1*	FOS like 1, AP-1 transcription factor subunit	3.063	0.000
*FLG*	filaggrin	3.037	0.031
*CXCL8*	C-X-C motif chemokine ligand 2	2.510	0.003
*PTGS2*	prostaglandin-endoperoxide synthase 2	2.424	0.000
*FGF19*	fibroblast growth factor 19	2.400	0.000
*MYC*	N-myc downstream regulated 1	2.164	0.000
*SERPINE1*	serpin family E member 1	2.143	0.000
*HOXA5*	homeobox A5	−2.043	0.000
*ERBB3*	erb-b2 receptor tyrosine kinase 3	−2.140	0.000
*IRS2*	insulin receptor substrate 2	−2.605	0.000
*FGFR2*	fibroblast growth factor receptor 2	−2.613	0.000
*CBLB*	Cbl proto-oncogene B	−3.391	0.000
*BCL6*	B-cell CLL/lymphoma 6	−3.718	0.000
*BTG2*	BTG anti-proliferation factor 2	−4.712	0.000

## Data Availability

The datasets supporting the conclusions of this article are included within the article and its additional files. The raw sequence data have been submitted to the Sequence Read Archive (SRA) under Bioproject accession number PRJNA792019 (https://www.ncbi.nlm.nih.gov/sra/ PRJNA792019 (accessed on 26 May 2022)) and reference BioSample accession number SAMN24371861–9 (https://www.ncbi.nlm.nih.gov/biosample/24371861–9 (accessed on 26 May 2022)).

## Supplementary Materials

The following supporting information can be downloaded at: https://www.mdpi.com/article/10.3390/microorganisms10071276/s1. Table S1: Primers used for RT-qPCR for selected genes, Table S2: Throughput and quality of RNA-seq data, Table S3: List of differentially expressed genes between wild-type spore-stimulated HT-29 cells and control, Table S4: List of differentially expressed genes between CotG-p75-stimulated HT-29 cells and control, Table S5: List of differentially expressed genes between CotG-p75-stimulated and wild-type spore-stimulated HT-29 cells, Table S6: Gene ontology (GO) enrichment analysis of differentially expressed genes in HT-29 stimulated with wild-type spore, Table S7: Gene ontology (GO) enrichment analysis of differentially expressed genes in HT-29 stimulated with CotG-p75, Figure S1: Effect of wild-type spores (**a**) and CotG-p75 (**b**) on cell viability of intestinal epithelial HT-29 cells, Figure S2: Illustration of gene ontology (GO) term networks of differentially expressed genes between wild-type spore-stimulated HT-29 cells and control, Figure S3: The mRNA expression of the genes involved in cell survival and proliferation from HT-29 stimulated by different doses of CotG-p75, Figure S4: The mRNA expression of the genes involved in epithelium development and differentiation from HT-29 stimulated by different doses of CotG-p75, Figure S5: TNF signaling pathway in KEGG pathway analysis., Figure S6: Hippo signaling pathway in KEGG pathway analysis.
