# Supplementary material for: Effects of Spore-Displayed p75 Protein from Lacticaseibacillus rhamnosus GG on the Transcriptional Response of HT-29 Cells
Source: Microorganisms. 2022 Jun 23;10(7):1276. doi: 10.3390/microorganisms10071276 (PMC9323162; doi:10.3390/microorganisms10071276)
Supplement: Supplementary file 1 [file microorganisms-10-01276-s001.zip › microorganisms-1771067-supplementary.pdf]

Journal name:

Microorganisms

Manuscript title:

Effects of spore-displayed p75 protein from *Lacticaseibacillus rhamnosus* GG on the transcriptional response of HT-29 cells

The names of the authors:

Soo Ji Kang[1], Min Joo Kim[1], Do Yeong Son[1], Seok Seong Kang[1], and Kwang Won Hong[1,*]

The affiliation and address of the authors:

[1] Department of Food Science and Biotechnology, College of Life Science and Biotechnology, Dongguk University, Goyang-si 10326, Republic of Korea

*** Corresponding author:** Kwang Won Hong (E-mail address: hkwon@dongguk.edu, Tel: +82-31-961-5140, Fax: +82-31-961-5108)

Table S1 Primers used for RT-qPCR for selected genes

Accession number	Gene symbols	Forward primer (5'→3')	Reverse primer (5'→3')
NM_001657	*AREG*	GCACCTGGAAGCAGTAACATGC	GGCAGCTATGGCTGCTAATGCA
NM_001432	*EREG*	CTTATCACAGTCGTCGGTTCCAC	GCCATTCAGACTTGCGGCAACT
NM_001945	*HBEGF*	TGTATCCACGGACCAGCTGCTA	TGCTCCTCCTTGTTTGGTGTGG
NM_001013442	*EPGN*	GTTCTCACACCTTTGCCTGGAAG	GTCAAGTGCTCACACCTTTCTCC
NM_000639	*FASLG*	GGTTCTGGTTGCCTTGGTAGGA	CTGTGTGCATCTGGCTGGTAGA
NM_005270	*GLI2*	GTCAGAGCCATCAAGACCGAGA	GCATCTCCACGCCACTGTCATT
NM_000389	*CDKN1A*	AGGTGGACCTGGAGACTCTCAG	TCCTCTTGGAGAAGATCAGCCG
NM_005438	*FOSL1*	GGAGGAAGGAACTGACCGACTT	CTCTAGGCGCTCCTTCTGCTTC
NM_002467	*MYC*	CCTGGTGCTCCATGAGGAGAC	CAGACTCTGACCTTTTGCCAGG
NM_000602	*SERPINE1*	CTCATCAGCCACTGGAAAGGCA	GACTCGTGAAGTCAGCCTGAAAC
NM_003810	*TNFSF10*	TGGCAACTCCGTCAGCTCGTTA	AGCTGCTACTCTCTGAGGACCT
NM_001706	*BCL6*	CATGCAGAGATGTGCCTCCACA	TCAGAGAAGCGGCAGTCACACT
NM_002016	*FLG*	GCTGAAGGAACTTCTGGAAAAGG	GTTGTGGTCTATATCCAAGTGATC
NM_005547	*IVL*	GGTCCAAGACATTCAACCAGCC	TCTGGACACTGCGGGTGGTTAT
NM_005987	*SPRR1A*	GTGAAACAACCTTGCCAGCCTC	TGGCAGGGCTCTGGAACCTTG
NM_003125	*SPRR1B*	CTGCCCTTCAATAGTCACTCCAG	CTCATACGCAGAATGGGATAGGG
NM_001097589	*SPRR3*	TGAACCAGGCAGCATCAAGGTC	GAAGGACATGGCTCTGGTAGCT
NM_017511	*MUC5AC*	CCACTGGTTCTATGGCAACACC	GCCGAAGTCCAGGCTGTGCG

Table S2 Throughput and quality of RNA-seq data

Sample name[a]	Raw reads	Clean reads	GC content (%)	Q30 (%)[b]	Mapped reads[c]
CON-1	78,305,704	77,090,466	50.09	96.08	76,116,020 (98.74%)
CON-2	69,390,858	67,876,956	46.84	95.54	65,763,348 (96.89%)
CON-3	79935,542	78,407,308	46.98	95.53	74,804,251 (95.4%)
WT-1	80,578,234	79,502,480	49.40	96.25	78,534,207 (98.78%)
WT-2	63,678,206	62,388,808	48.07	95.69	61,301,961 (98.26%)
WT-3	59,788,048	58,381,418	47.45	95.54	57,391,672 (98.3%)
G75-1	69,795,644	68,885,098	49.98	96.53	67,886,087 (98.55%)
G75-2	81,607,548	79,218,066	48.56	95.74	77,111,419 (97.34%)
G75-3	87,526,726	86,105,694	46.82	95.69	84,492,377 (98.13%)

[a] Each sample represents HT-29 cells unstimulated (CON) or stimulated with wild-type spore (WT) and CotG-p75 (G75), respectively

[b] Q30 indicates the percentage of sequencing data with an error rate less than 0.1%

[c] Mapped reads represent the reads of clean bases mapped to the reference genome

Table S3 List of differentially expressed genes between wild-type spores stimulated HT-29 cells and control

Gene ID	Transcript ID	Gene Symbol	Description	Fold change (WT/CON)	Raw *p*-val
100532736	NM_001204088,NM_001204089	MINOS1-NBL1	MINOS1-NBL1 readthrough	5.711302	0.030415887
140710	NM_080627,NM_199181	SOGA1	suppressor of glucose, autophagy associated 1	4.993611	7.22189E-18
147111	NM_178493	NOTUM	NOTUM, palmitoleoyl-protein carboxylesterase	4.697269	0.011976435
5463	NM_001330422,NM_001351088,NR_026893,NR_147046,NR_147047,NR_147048,NR_147049,NR_147050,NR_147051,NR_147052	POU6F1	POU class 6 homeobox 1	4.234451	0.002442382
5243	NM_000927,NM_001348944,NM_001348945,NM_001348946	ABCB1	ATP binding cassette subfamily B member 1	4.049126	0.034347626
201181	NM_001242704	ZNF385C	zinc finger protein 385C	3.950943	0.014494083
80125	NM_001287181,NM_025055,NM_182791	CCDC33	coiled-coil domain containing 33	3.618958	0.03014839
9501	NM_001190411,NM_001190412,NM_001190413,NM_006987	RPH3AL	rabphilin 3A like (without C2 domains)	3.602920	0.000907002
2979	NM_002098	GUCA1B	guanylate cyclase activator 1B	3.416902	0.005862771
9154	NM_001287761,NM_001287762,NM_001321721,NM_001321722,NM_004213,NM_201651	SLC28A1	solute carrier family 28 member 1	3.218961	0.029048749

401251	NM_001039651	SAPCD1	suppressor APC domain containing 1	3.212568	0.033779138
108903150	NR_144447	ZNF8-ERVK3-1	ZNF8-ERVK3-1 readthrough	3.157648	1.56552E-05
203102	NM_001313994,NM_145004	ADAM32	ADAM metallopeptidase domain 32	3.080939	0.041062969
7846	NM_001270399,NM_001270400,NM_006009	TUBA1A	tubulin alpha 1a	3.004302	0.045567622
160428	NM_001034173,NR_027752	ALDH1L2	aldehyde dehydrogenase 1 family member L2	2.988939	0.030491199
23148	NM_001146334	NACAD	NAC alpha domain containing	2.662285	0.043102239
400618	NR_103737,NR_103738	SOX9-AS1	SOX9 antisense RNA 1	2.618078	0.010155644
79413	NM_024508	ZBED2	zinc finger BED-type containing 2	2.602981	0.002156233
80790	NM_030629,NM_198390	CMIP	c-Maf inducing protein	2.566630	0.019739208
138199	NM_001320497,NM_152420,NR_135282	CARNMT1	carnosine N-methyltransferase 1	2.564506	0.00687444
83886	NM_001318395,NM_031948	PRSS27	protease, serine 27	2.440817	0.04065593
677825	NR_002976	SNORA44	small nucleolar RNA, H/ACA box 44	2.353757	0.041160895
7352	NM_003356,NM_022803	UCP3	uncoupling protein 3	2.294909	0.015013487
7052	NM_001323316,NM_001323317,NM_001323318,NM_004613,NM_198951	TGM2	transglutaminase 2	2.283907	0.026895712
25764	NM_001199885,NM_016400	HYPK	huntingtin interacting protein K	2.258744	0.006963514

101928689	NR_109928	LINC01424	long intergenic non-protein coding RNA 1424	2.244268	0.048553463
6364	NM_001130046,NM_004591	CCL20	C-C motif chemokine ligand 20	2.166385	0.008796954
2920	NM_002089	CXCL2	C-X-C motif chemokine ligand 2	2.154919	0.021575445
2840	NM_001161415,NM_001161416,NM_001161417,NM_005291	GPR17	G protein-coupled receptor 17	2.154634	0.015847274
163590	NM_001199260,NM_001349931,NM_001349933,NM_001349934,NM_001349935,NM_001349936,NM_001349937,NM_022347,NM_145034	TOR1AIP2	torsin 1A interacting protein 2	2.108915	0.01493783
254099	NR_038869	LINC01342	long intergenic non-protein coding RNA 1342	2.082970	0.039009893
728658	NR_026712	RPL13AP5	ribosomal protein L13a pseudogene 5	2.080822	0.005238317
29114	NM_001008272,NM_001008273,NM_013259	TAGLN3	transgelin 3	2.076381	0.019636428
55588	NM_001317770,NM_017592,NR_133915	MED29	mediator complex subunit 29	2.047612	0.012890371
6640	NM_003098	SNTA1	syntrophin alpha 1	2.002687	0.038991439
100529144	NM_001201479	CORO7-PAM16	CORO7-PAM16 readthrough	-2.013298	0.009531695
200315	NM_001270406,NM_145699	APOBEC3A	apolipoprotein B mRNA editing enzyme catalytic subunit 3A	-2.015495	0.043606522
9991	NM_001163788,NM_001163790,NM_001244896,NM_001244897,NM_001244898,	PTBP3	polypyrimidine tract binding protein 3	-2.023599	0.046782533

	NM_005156				
5166	NM_002612	PDK4	pyruvate dehydrogenase kinase 4	-2.028992	0.000216423
8732	NM_001286426,NM_001286428,NM_003800	RNGTT	RNA guanylyltransferase and 5'-phosphatase	-2.035854	0.006541802
121268	NM_001303126,NM_144593,NR_130123	RHEBL1	Ras homolog enriched in brain like 1	-2.039483	0.003287191
23306	NM_001130963,NM_015257	NEMP1	nuclear envelope integral membrane protein 1	-2.148945	0.015217889
2583	NM_001276468,NM_001276469,NM_001478	B4GALNT1	beta-1,4-N-acetyl-galactosaminyltransferase 1	-2.158066	0.012364564
7802	NM_003462	DNALI1	dynein axonemal light intermediate chain 1	-2.168669	0.009393765
8339	NM_003518	HIST1H2BG	histone cluster 1 H2B family member g	-2.316632	0.001106169
101929549	NR_110112	LOC101929549	uncharacterized LOC101929549	-2.364050	0.029187118
105371267	NR_136518,NR_147075	LOC105371267	p53-regulated lncRNA 1	-2.379005	0.040032435
1719	NM_000791,NM_001290354,NM_001290357,NR_110936	DHFR	dihydrofolate reductase	-2.453515	0.032538864
377841	NM_001033113,NM_198585	ENTPD8	ectonucleoside triphosphate diphosphohydrolase 8	-2.460757	0.035359747
100996724	NR_144516,NR_144517	LOC100996724	phosphodiesterase 4D interacting protein-like	-2.479262	0.00749269
26206	NM_001039592,NM_172312	SPAG8	sperm associated antigen 8	-2.564928	0.043915365

9403	NM_004261,NM_203341,NR_144512,NR_144513	SELENOF	selenoprotein F	-2.673010	0.015073822
2901	NM_001301030,NM_002088	GRIK5	glutamate ionotropic receptor kainate type subunit 5	-2.736920	0.032389078
10891	NM_001330751,NM_001330752,NM_001330753,NM_013261	PPARGC1A	PPARG coactivator 1 alpha	-2.749119	0.020904192
161753	NM_175881	ODF3L1	outer dense fiber of sperm tails 3 like 1	-2.805109	0.045557441
653113	NR_024254	FAM86FP	family with sequence similarity 86 member F, pseudogene	-2.836866	0.002604823
378938	NR_002819,NR_144567,NR_144568	MALAT1	metastasis associated lung adenocarcinoma transcript 1 (non-protein coding)	-2.923931	0.015785377
677767	NR_003001	SCARNA7	small Cajal body-specific RNA 7	-2.937906	0.018131273
29071	NM_001011551,NM_152692	C1GALT1C1	C1GALT1 specific chaperone 1	-2.982188	0.023708309
6345	NM_001098814,NM_001323667,NM_001323668	SRL	sarcalumenin	-2.983180	0.03587902
8351	NM_003530	HIST1H3D	histone cluster 1 H3 family member d	-3.158637	0.003155483
144193	NM_152435	AMDHD1	amidohydrolase domain containing 1	-3.264341	0.010144148
8348	NM_003527	HIST1H2BO	histone cluster 1 H2B family member o	-3.389114	0.007510994
85495	NR_002312	RPPH1	ribonuclease P RNA component H1	-3.530340	0.000607651

8329	NM_003509	HIST1H2AI	histone cluster 1 H2A family member i	-3.594152	0.004087093
100507266	NR_037888	STX18-AS1	STX18 antisense RNA 1 (head to head)	-3.635330	0.03025544
8343	NM_003522	HIST1H2BF	histone cluster 1 H2B family member f	-3.772859	0.00907002
106099130	NR_132348	GSTT1-AS1	GSTT1 antisense RNA 1	-3.778788	0.035325805
344657	NM_001080460	LRRIQ4	leucine rich repeats and IQ motif containing 4	-3.915645	0.031801416
6023	NR_003051	RMRP	RNA component of mitochondrial RNA processing endoribonuclease	-5.073627	0.017091649
126961	NM_021059,NM_021059_dup1	HIST2H3C	histone cluster 2 H3 family member c	-5.492560	0.020315907
121274	NM_001172681,NM_001172682,NM_152320	ZNF641	zinc finger protein 641	-9.394567	3.68015E-06
904	NM_001240,NM_001277842	CCNT1	cyclin T1	-12.019002	0.044362546
101060321	NM_001291462,NM_001291462_dup1,NM_001291462_dup2,NM_001291462_dup3	TBC1D3G	TBC1 domain family member 3G	-27.240369	0.044164274

Table S4 List of differentially expressed genes between CotG-p75 stimulated HT-29 cells and control

Gene ID	Transcript ID	Gene Symbol	Description	Fold change (G75/CON)	Raw *p*-val
1543	NM_000499,NM_001319216,NM_001319217	CYP1A1	cytochrome P450 family 1 subfamily A member 1	191.945423	1.37257E-78
29114	NM_001008272,NM_001008273,NM_013259	TAGLN3	transgelin 3	11.148347	2.28108E-17
1545	NM_000104	CYP1B1	cytochrome P450 family 1 subfamily B member 1	9.239607	0
51702	NM_016233	PADI3	peptidyl arginine deiminase 3	8.747112	6.33867E-33
220	NM_000693,NM_001293815	ALDH1A3	aldehyde dehydrogenase 1 family member A3	8.154219	2.7368E-298
221472	NM_173558	FGD2	FYVE, RhoGEF and PH domain containing 2	7.183803	6.58136E-05
2840	NM_001161415,NM_001161416,NM_001161417, NM_005291	GPR17	G protein-coupled receptor 17	6.522831	1.61687E-10
8120	NM_001278511,NM_001278512,NM_001348440, NM_001348441,NM_004644	AP3B2	adaptor related protein complex 3 beta 2 subunit	5.699657	1.19502E-05
2736	NM_005270	GLI2	GLI family zinc finger 2	5.524409	3.97038E-15
6699	NM_003125	SPRR1B	small proline rich protein 1B	5.493314	7.581E-18
10170	NM_001142270,NM_001	DHRS9	dehydrogenase/reductase 9	5.329982	5.86E-45

	142271,NM_001289763,<br>NM_199204				
6707	NM_001097589,NM_005<br>416	SPRR3	small proline rich protein 3	5.304377	4.25519E-22
7133	NM_001066	TNFRSF1B	TNF receptor superfamily member 1B	5.271079	3.48243E-11
375791	NM_199001	CYSRT1	cysteine rich tail 1	5.104884	2.52469E-19
254439	NM_001136485	C11orf86	chromosome 11 open reading frame 86	5.057713	1.2881E-07
8482	NM_001146029,NM_001<br>146030,NM_003612	SEMA7A	semaphorin 7A (John Milton Hagen blood group)	4.909711	2.46531E-08
100846999	NR_049831_chr2,NR_04<br>9831_chr3	MIR4444-2	microRNA 4444-2	4.860051	0.005623582
2702	NM_005266,NM_181703	GJA5	gap junction protein alpha 5	4.842982	6.2038E-28
356	NM_000639,NM_001302<br>746	FASLG	Fas ligand	4.558933	0.00245931
7846	NM_001270399,NM_001<br>270400,NM_006009	TUBA1A	tubulin alpha 1a	4.474841	0.003847627
6398	NM_003004	SECTM1	secreted and transmembrane 1	4.285178	2.04839E-09
85409	NM_001271082,NM_033<br>120	NKD2	naked cuticle homolog 2	4.194734	9.85416E-44
255324	NM_001270989,NM_001<br>270990,NM_001270991,<br>NM_001270992,NM_001<br>270993,NR_073114,NR_	EPGN	epithelial mitogen	4.131741	0.013039185

	073117				
6698	NM_001199828,NM_005987	SPRR1A	small proline rich protein 1A	4.118056	0.00103516
164312	NM_152611	LRRN4	leucine rich repeat neuronal 4	4.113114	1.39408E-14
285154	NR_027252	CYP1B1-AS1	CYP1B1 antisense RNA 1	4.028174	2.49637E-10
5582	NM_001316329,NM_002739	PRKCG	protein kinase C gamma	3.946057	8.27897E-14
5463	NM_001330422,NM_001351088,NR_026893,NR_147046,NR_147047,NR_147048,NR_147049,NR_147050,NR_147051,NR_147052	POU6F1	POU class 6 homeobox 1	3.882133	0.002627753
9788	NM_001282971,NM_001282974,NM_014751	MTSS1	MTSS1, I-BAR domain containing	3.854426	4.87339E-31
144453	NM_001282613,NM_001282614,NM_001282615,NM_001282616,NM_032735,NM_152439,NR_104220	BEST3	bestrophin 3	3.841074	0.016040629
219855	NM_001145290,NM_198277	SLC37A2	solute carrier family 37 member 2	3.830376	1.98055E-16
6364	NM_001130046,NM_004591	CCL20	C-C motif chemokine ligand 20	3.804218	1.20057E-06
245973	NM_001039362,NM_144	ATP6V1C2	ATPase H+ transporting V1 subunit C2	3.752930	8.74923E-08

	583				
3038	NM_001199280,NM_005329,NM_138612	HAS3	hyaluronan synthase 3	3.717783	4.88841E-09
79094	NM_001142776,NM_024111	CHAC1	ChaC glutathione specific gamma-glutamylcyclotransferase 1	3.688559	1.09396E-10
7433	NM_001251882,NM_001251883,NM_001251884,NM_001251885,NM_004624	VIPR1	vasoactive intestinal peptide receptor 1	3.611321	3.70838E-81
885	NM_000729,NM_001174138	CCK	cholecystokinin	3.610318	0.001486433
101929125	NR_109859	LINC01730	long intergenic non-protein coding RNA 1730	3.541326	0.008034132
140710	NM_080627,NM_199181	SOGA1	suppressor of glucose, autophagy associated 1	3.466092	3.41389E-12
117157	NM_053282	SH2D1B	SH2 domain containing 1B	3.455596	0.009690138
79413	NM_024508	ZBED2	zinc finger BED-type containing 2	3.453758	2.23807E-05
1026	NM_000389,NM_001220777,NM_001220778,NM_001291549,NM_078467	CDKN1A	cyclin dependent kinase inhibitor 1A	3.419799	8.01542E-94
340393	NM_001252402,NM_001252404,NM_001280561,NR_047684	TMEM249	transmembrane protein 249	3.334595	0.042034507
111	NM_001199642,NM_183357	ADCY5	adenylate cyclase 5	3.324753	0.049279363

25907	NM_015444	TMEM158	transmembrane protein 158 (gene/pseudogene)	3.320155	8.27219E-18
54541	NM_019058	DDIT4	DNA damage inducible transcript 4	3.271894	6.70371E-70
84623	NM_001161707,NM_001301097,NM_032531	KIRREL3	kin of IRRE like 3 (Drosophila)	3.228350	9.17243E-05
1437	NM_000758	CSF2	colony stimulating factor 2	3.225479	5.19346E-08
2069	NM_001432	EREG	epiregulin	3.221198	2.0928E-109
7128	NM_001270507,NM_001270508,NM_006290	TNFAIP3	TNF alpha induced protein 3	3.187974	2.03684E-11
3352	NM_000864	HTR1D	5-hydroxytryptamine receptor 1D	3.169428	7.01724E-41
6624	NM_003088	FSCN1	fascin actin-bundling protein 1	3.167804	1.7598E-07
9940	NM_001321153,NM_007335,NM_007337	DLEC1	deleted in lung and esophageal cancer 1	3.167403	1.32736E-05
78990	NM_023112	OTUB2	OTU deubiquitinase, ubiquitin aldehyde binding 2	3.148013	1.00829E-20
8193	NM_001135155,NM_001135156,NM_001289978, NM_004647	DPF1	double PHD fingers 1	3.144756	0.025166525
9841	NM_001164313,NM_014797	ZBTB24	zinc finger and BTB domain containing 24	3.133866	1.28589E-10
9536	NM_004878	PTGES	prostaglandin E synthase	3.128326	0.045531525
3384	NM_000873,NM_001099786,NM_001099787,NM_001099788,NM_001099	ICAM2	intercellular adhesion molecule 2	3.120727	0.019057569

	789				
8862	NM_017413	APLN	apelin	3.107111	0.020805675
147929	NM_001042474,NM_152477	ZNF565	zinc finger protein 565	3.077691	4.20123E-54
8061	NM_001300844,NM_001300855,NM_001300856,NM_001300857,NM_005438,NR_125339	FOSL1	FOS like 1, AP-1 transcription factor subunit	3.062739	0.01895985
101928841	NM_001304433	LOC101928841	collagen alpha-1(II) chain-like	3.045369	1.42336E-45
3872	NM_000422	KRT17	keratin 17	3.044978	0.030591911
2312	NM_002016	FLG	filaggrin	3.036836	4.20727E-05
1735	NM_001362	DIO3	iodothyronine deiodinase 3	3.036003	0.009405687
57514	NM_020754	ARHGAP31	Rho GTPase activating protein 31	3.026232	0.007540874
55084	NM_018013	SOBP	sine oculis binding protein homolog	3.021249	4.01005E-52
57214	NM_001293298,NM_001293304,NM_018689	CEMIP	cell migration inducing hyaluronan binding protein	3.009316	0.003029468
2331	NM_002023,NR_103757	FMOD	fibromodulin	3.001652	4.58295E-24
3283	NM_000862,NM_001328615	HSD3B1	hydroxy-delta-5-steroid dehydrogenase, 3 beta- and steroid delta-isomerase 1	2.986105	0.020496109
10896	NM_022375	OCLM	oculomedin	2.963107	0.002233085
100131378	NM_001166692	C11orf91	chromosome 11 open reading frame 91	2.943090	0.03958522

6317	NM_006919	SERPINB3	serpin family B member 3	2.932880	0.011226637
2979	NM_002098	GUCA1B	guanylate cyclase activator 1B	2.925289	1.04219E-75
374	NM_001657,NM_001657_dup1	AREG	amphiregulin	2.792427	2.42195E-49
10123	NM_001282431,NM_005737	ARL4C	ADP ribosylation factor like GTPase 4C	2.760695	0.004434847
2498	NR_002201	FTH1P3	ferritin heavy chain 1 pseudogene 3	2.754462	0.000888934
6536	NM_001024845,NM_001261380,NM_001328626,NM_001328627,NM_001328628,NM_001328629,NM_001328630,NM_006934,NM_201649,NR_048548	SLC6A9	solute carrier family 6 member 9	2.744465	8.59642E-80
54206	NM_018948	ERRFI1	ERBB receptor feedback inhibitor 1	2.722261	0.022235995
259236	NM_147196	TMIE	transmembrane inner ear	2.659485	0.000116243
84981	NR_028502,NR_028503,NR_028504,NR_028505	MIR22HG	MIR22 host gene	2.635537	2.76889E-59
126917	NM_001136265	IFFO2	intermediate filament family orphan 2	2.616536	0.022407478
56241	NM_019601	SUSD2	sushi domain containing 2	2.614846	0.003347381
117155	NM_001282309,NM_001282310,NM_172095,NR_110319	CATSPER2	cation channel sperm associated 2	2.610799	1.81529E-46

84803	NM_001256421,NM_001256422,NM_032717	GPAT3	glycerol-3-phosphate acyltransferase 3	2.608791	0.039721486
171169	NM_133498	SPACA4	sperm acrosome associated 4	2.607807	1.08142E-62
266977	NM_025048,NM_153840	ADGRF1	adhesion G protein-coupled receptor F1	2.603004	0.001633467
100506737	NR_131244	LINC01589	long intergenic non-protein coding RNA 1589	2.602534	7.62893E-39
100499467	NR_036488,NR_137280, NR_137281	LINC00673	long intergenic non-protein coding RNA 673	2.576686	3.91133E-13
137994	NM_001199659,NM_001199660,NM_001286787, NM_001286819,NM_001286821,NM_001330515, NM_144652	LETM2	leucine zipper and EF-hand containing transmembrane protein 2	2.572450	4.03826E-42
360	NM_001318144,NM_004925	AQP3	aquaporin 3 (Gill blood group)	2.570650	6.2413E-35
23135	NM_001080424,NM_001348716	KDM6B	lysine demethylase 6B	2.544534	1.04954E-48
91663	NM_001020818,NM_001020819,NM_001020820, NM_001020821,NM_001290188,NM_001290189, NM_001290190,NM_001290191,NM_001290192, NM_001290193,NM_001290194,NM_138373	MYADM	myeloid associated differentiation marker	2.539238	7.46149E-05
100499177	NR_034075,NR_034076, NR_034077	THAP9-AS1	THAP9 antisense RNA 1	2.529425	1.95098E-07

164832	NM_198461	LONRF2	LON peptidase N-terminal domain and ring finger 2	2.522746	2.46176E-05
399473	NM_001042522,NR_073032	SPRED3	sprouty related EVH1 domain containing 3	2.518955	4.04157E-05
26086	NM_001145638,NM_001145639,NM_001200003, NM_015597	GPSM1	G protein signaling modulator 1	2.517783	1.3941E-13
11010	NM_006851	GLIPR1	GLI pathogenesis related 1	2.516977	1.90843E-34
168455	NM_175884	CCDC71L	coiled-coil domain containing 71 like	2.516709	0.003452083
2920	NM_002089	CXCL2	C-X-C motif chemokine ligand 2	2.509510	6.60711E-09
4586	NM_001304359	MUC5AC	mucin 5AC, oligomeric mucus/gel-forming	2.492205	1.6051E-34
9518	NM_004864	GDF15	growth differentiation factor 15	2.477376	8.96234E-17
11240	NM_007365	PADI2	peptidyl arginine deiminase 2	2.467617	0.014713806
84290	NM_032330	CAPNS2	calpain small subunit 2	2.444739	0.042235493
25758	NM_012194	KIAA1549L	KIAA1549 like	2.440597	0.000638616
3371	NM_002160	TNC	tenascin C	2.439789	9.53819E-27
283174	NR_024344	MIR4697HG	MIR4697 host gene	2.437494	3.74979E-12
57491	NM_001242412,NM_020731	AHRR	aryl-hydrocarbon receptor repressor	2.437478	5.53821E-10
6581	NM_021977	SLC22A3	solute carrier family 22 member 3	2.432945	5.10832E-38
5743	NM_000963	PTGS2	prostaglandin-endoperoxide synthase 2	2.424420	1.41594E-43

112574	NM_001102575,NM_001145427,NM_052870	SNX18	sorting nexin 18	2.422601	1.22828E-25
3576	NM_000584	CXCL8	C-X-C motif chemokine ligand 8	2.417164	2.81662E-52
9965	NM_005117	FGF19	fibroblast growth factor 19	2.400426	6.31072E-15
29126	NM_001267706,NM_001314029,NM_014143,NR_052005	CD274	CD274 molecule	2.381806	0.000802025
6781	NM_003155	STC1	stanniocalcin 1	2.381257	1.62214E-10
84541	NM_032505	KBTBD8	kelch repeat and BTB domain containing 8	2.357111	3.47238E-20
23328	NM_001346505,NM_001346506,NM_001346507, NM_001346508,NM_001346509,NM_015278	SASH1	SAM and SH3 domain containing 1	2.335536	0.004282334
2669	NM_005261,NM_181702	GEM	GTP binding protein overexpressed in skeletal muscle	2.327218	1.42652E-25
29943	NM_013358	PADI1	peptidyl arginine deiminase 1	2.318097	7.71605E-59
7791	NM_001010972,NM_003461	ZYX	zyxin	2.317488	0.007893833
6640	NM_003098	SNTA1	syntrophin alpha 1	2.312051	0.000233849
4242	NM_001166343,NM_002405,NR_029413	MFNG	MFNG O-fucosylpeptide 3-beta-N-acetylglucosaminyltransferase	2.310436	0.008476167
168620	NM_177455	BHLHA15	basic helix-loop-helix family member a15	2.276086	9.01723E-32
9590	NM_005100,NM_144497	AKAP12	A-kinase anchoring protein 12	2.273980	1.00901E-23

2571	NM_000817,NM_013445	GAD1	glutamate decarboxylase 1	2.271789	5.99216E-65
8140	NM_003486	SLC7A5	solute carrier family 7 member 5	2.263884	1.07369E-71
22822	NM_007350	PHLDA1	pleckstrin homology like domain family A member 1	2.260046	0.001027526
1958	NM_001964	EGR1	early growth response 1	2.259638	0.000179646
339894	NR_034007	LINC00880	long intergenic non-protein coding RNA 880	2.256482	8.17417E-71
6484	NM_001254757,NM_001254758,NM_001254759,NM_001348396,NM_001348397,NM_001348398,NM_001348399,NM_001348400,NM_006278,NR_145671	ST3GAL4	ST3 beta-galactoside alpha-2,3-sialyltransferase 4	2.232742	1.72609E-26
65981	NM_001002259,NM_001206856,NM_001319842,NM_001319843,NM_001319844,NM_001319845,NM_001319846,NM_023925,NM_032156	CAPRIN2	caprin family member 2	2.223298	0.002073422
10137	NM_001198838,NM_001198840,NM_006047,NM_152838	RBM12	RNA binding motif protein 12	2.220596	2.00214E-15
130497	NM_145260	OSR1	odd-skipped related transciption factor 1	2.215743	0.02431871
677839	NR_003017	SNORA71C	small nucleolar RNA, H/ACA box 71C	2.206251	0.02431871
57473	NM_020713	ZNF512B	zinc finger protein 512B	2.197257	0.004020972

100505994	NR_103548,NR_103549	LUCAT1	lung cancer associated transcript 1 (non-protein coding)	2.194235	0.00016789
108903150	NR_144447	ZNF8-ERVK3-1	ZNF8-ERVK3-1 readthrough	2.191464	0.001775745
6513	NM_006516	SLC2A1	solute carrier family 2 member 1	2.189117	5.15137E-57
11227	NM_001329868,NM_014568	GALNT5	polypeptide N-acetylgalactosaminyltransferase 5	2.186057	1.55485E-08
1839	NM_001945	HBEGF	heparin binding EGF like growth factor	2.180764	9.10819E-06
9120	NM_001174166,NM_004694	SLC16A6	solute carrier family 16 member 6	2.178564	0.016133014
100507178	NR_037868	SLFNL1-AS1	SLFNL1 antisense RNA 1	2.178044	0.0059774
22997	NM_001277285	IGSF9B	immunoglobulin superfamily member 9B	2.177263	1.64836E-16
9586	NM_001011666,NM_004904,NM_182898,NM_182899	CREB5	cAMP responsive element binding protein 5	2.170680	7.08338E-07
4188	NM_001300804,NM_001300805,NM_001300806,NM_005586	MDFI	MyoD family inhibitor	2.169043	0.001280461
100820829	NM_001018100,NM_152451	MYZAP	myocardial zonula adherens protein	2.163825	1.93088E-33
10397	NM_001135242,NM_001258432,NM_001258433,NM_006096	NDRG1	N-myc downstream regulated 1	2.163677	4.67021E-14
80153	NM_001142443,NM_001	EDC3	enhancer of mRNA decapping 3	2.145487	1.20944E-52

	142444,NM_001351378, NM_001351379,NM_025083				
90102	NM_001134437,NM_001134438,NM_001134439, NM_145753	PHLDB2	pleckstrin homology like domain family B member 2	2.143867	1.26832E-12
5054	NM_000602	SERPINE1	serpin family E member 1	2.142781	3.73661E-06
3713	NM_005547	IVL	involucrin	2.141007	1.17282E-07
7477	NM_058238	WNT7B	Wnt family member 7B	2.134060	1.65228E-32
8572	NM_001131027,NM_003687	PDLIM4	PDZ and LIM domain 4	2.133899	0.021724785
6722	NM_001292001,NM_003131	SRF	serum response factor	2.123975	0.046328683
9957	NM_005114	HS3ST1	heparan sulfate-glucosamine 3-sulfotransferase 1	2.122846	2.54439E-43
5795	NM_001098503,NM_002843	PTPRJ	protein tyrosine phosphatase, receptor type J	2.122527	5.0724E-37
4609	NM_002467	MYC	MYC proto-oncogene, bHLH transcription factor	2.120155	1.33277E-40
23657	NM_014331	SLC7A11	solute carrier family 7 member 11	2.101824	8.63694E-37
250	NM_001632	ALPP	alkaline phosphatase, placental	2.101648	0.041074205
2706	NM_004004	GJB2	gap junction protein beta 2	2.097460	2.46252E-43
8771	NM_003823	TNFRSF6B	TNF receptor superfamily member 6b	2.091485	0.028242108
89870	NM_033229	TRIM15	tripartite motif containing 15	2.080390	8.36575E-18

55901	NM_018676,NM_199263	THSD1	thrombospondin type 1 domain containing 1	2.079871	0.017245524
23109	NM_015086	DDN	dendrin	2.075644	0.032912451
22936	NM_012081	ELL2	elongation factor for RNA polymerase II 2	2.075086	3.98991E-29
10019	NM_001291424,NM_005475	SH2B3	SH2B adaptor protein 3	2.073470	2.1724E-09
805	NM_001305624,NM_001305625,NM_001305626,NM_001743	CALM2	calmodulin 2	2.072885	1.03106E-25
55243	NM_001286349,NM_018240	KIRREL	kin of IRRE like (Drosophila)	2.063314	3.11531E-11
27063	NM_014391	ANKRD1	ankyrin repeat domain 1	2.061869	0.002724582
8728	NM_033274	ADAM19	ADAM metallopeptidase domain 19	2.053270	1.58861E-21
163732	NM_133467	CITED4	Cbp/p300 interacting transactivator with Glu/Asp rich carboxy-terminal domain 4	2.052827	0.001930559
399972	NR_033839	GSEC	G-quadruplex forming sequence containing lncRNA	2.051453	7.63169E-05
1045	NM_001265	CDX2	caudal type homeobox 2	2.049061	5.8418E-05
1506	NM_001907	CTRL	chymotrypsin like	2.048620	0.000331527
6004	NM_002928	RGS16	regulator of G protein signaling 16	2.046753	9.28378E-07
1847	NM_004419	DUSP5	dual specificity phosphatase 5	2.038920	2.83221E-17
116936	NR_000026	SNORD139	small nucleolar RNA, C/D box 139	2.035013	0.020342554

84951	NM_032865	TNS4	tensin 4	2.032424	2.01517E-42
59285	NM_031897,NM_145814,NM_145815,NR_102308	CACNG6	calcium voltage-gated channel auxiliary subunit gamma 6	2.030350	1.70511E-06
5778	NM_001199797,NM_002832,NM_080588,NR_037663,NR_037664	PTPN7	protein tyrosine phosphatase, non-receptor type 7	2.027542	1.75707E-14
254427	NM_153256	PROSER2	proline and serine rich 2	2.027244	3.46484E-34
3556	NM_001167928,NM_001167929,NM_001167930,NM_001167931,NM_002182,NM_134470	IL1RAP	interleukin 1 receptor accessory protein	2.027198	4.34928E-08
105374177	NR_135546	LINC02029	long intergenic non-protein coding RNA 2029	2.025677	0.000717704
64651	NM_001320559,NM_001320560,NM_033027	CSRNP1	cysteine and serine rich nuclear protein 1	2.021440	1.91772E-12
3628	NM_001128928,NM_002194	INPP1	inositol polyphosphate-1-phosphatase	2.012929	4.97391E-38
9388	NM_001308006,NM_006033	LIPG	lipase G, endothelial type	2.009272	6.67785E-37
440193	NM_001080414	CCDC88C	coiled-coil domain containing 88C	2.006184	4.42887E-41
221468	NM_001162900,NM_001286401,NM_145316,NR_104442,NR_104443	TMEM217	transmembrane protein 217	2.003065	0.002278448
79465	NM_024518	ULBP3	UL16 binding protein 3	2.002097	1.22755E-18

400673	NM_001017921	VMAC	vimentin-type intermediate filament associated coiled-coil protein	-2.001977	0.0020581
57167	NM_001318031,NM_020436	SALL4	spalt like transcription factor 4	-2.003012	1.41372E-06
56260	NM_019607	C8orf44	chromosome 8 open reading frame 44	-2.003040	9.1142E-05
84952	NM_001252335,NM_032866	CGNL1	cingulin like 1	-2.003991	4.99893E-05
387787	NM_001144869,NM_001329941,NM_001329942	LIPT2	lipoyl(octanoyl) transferase 2 (putative)	-2.005380	5.0905E-07
143872	NM_152432	ARHGAP42	Rho GTPase activating protein 42	-2.006057	1.32283E-13
25764	NM_001199885,NM_016400	HYPK	huntingtin interacting protein K	-2.014833	0.014075041
150759	NR_103734	LINC00342	long intergenic non-protein coding RNA 342	-2.018011	5.21346E-07
6843	NM_001297438,NM_014231,NM_016830,NM_199245,NR_123717	VAMP1	vesicle associated membrane protein 1	-2.018349	5.20101E-07
642366	NR_046243	LOC642366	uncharacterized LOC642366	-2.018567	1.21415E-07
26269	NM_012180	FBXO8	F-box protein 8	-2.020903	9.85161E-09
5652	NM_002773	PRSS8	protease, serine 8	-2.021801	7.91939E-37
192670	NM_017629,NR_146062	AGO4	argonaute 4, RISC catalytic component	-2.022005	3.82463E-18
253018	NR_026791	HCG27	HLA complex group 27 (non-protein coding)	-2.033615	0.003468705
203228	NM_001256054,NM_018	C9orf72	chromosome 9 open reading frame 72	-2.036136	0.000441187

	325,NM_145005				
286144	NM_001171795,NM_001171796,NM_001171797,NM_001171798,NM_001171799,NM_001191035,NM_001191036	TRIQK	triple QxxK/R motif containing	-2.037722	0.048780511
3707	NM_002221	ITPKB	inositol-trisphosphate 3-kinase B	-2.039248	2.17736E-13
83935	NM_032021	TMEM133	transmembrane protein 133	-2.039421	2.25113E-09
220359	NM_145719	TIGD3	tigger transposable element derived 3	-2.040293	0.005228195
27141	NM_001318807,NM_014430	CIDEB	cell death-inducing DFFA-like effector b	-2.042098	0.030950694
3202	NM_019102	HOXA5	homeobox A5	-2.042930	1.15683E-26
55593	NM_001136157,NM_001136158,NM_001136159,NM_017602	OTUD5	OTU deubiquitinase 5	-2.043956	2.91431E-05
51027	NM_001321025,NM_001321026,NM_016074	BOLA1	bolA family member 1	-2.047546	2.70265E-06
100505783	NR_038337,NR_038338,NR_038339	OSER1-AS1	OSER1 antisense RNA 1 (head to head)	-2.051336	7.95615E-07
9481	NM_001204051,NM_001204052,NM_004277	SLC25A27	solute carrier family 25 member 27	-2.053717	9.15261E-39
84181	NM_032221	CHD6	chromodomain helicase DNA binding protein 6	-2.056669	4.28386E-05
113220	NM_138424	KIF12	kinesin family member 12	-2.057832	0.0048857

285313	NM_001178145,NM_001178146,NM_178822	IGSF10	immunoglobulin superfamily member 10	-2.058676	5.01438E-05
11067	NM_007021	C10orf10	chromosome 10 open reading frame 10	-2.059031	1.46631E-13
341640	NM_207361	FREM2	FRAS1 related extracellular matrix protein 2	-2.059067	0.001120302
84140	NM_001201543,NM_032180,NR_037710	FAM161A	family with sequence similarity 161 member A	-2.062177	2.6933E-11
1945	NM_005227,NM_182689,NM_182690	EFNA4	ephrin A4	-2.067051	0.005749027
80741	NM_025262	LY6G5C	lymphocyte antigen 6 family member G5C	-2.068540	1.85093E-09
220213	NM_001145373	OTUD1	OTU deubiquitinase 1	-2.069453	1.44309E-13
8611	NM_003711,NM_176895,NR_103485	PLPP1	phospholipid phosphatase 1	-2.069463	2.36575E-30
57476	NM_001286563,NM_001286564,NM_001330396,NM_020716	GRAMD1B	GRAM domain containing 1B	-2.074839	1.75009E-05
2861	NM_005302	GPR37	G protein-coupled receptor 37	-2.075599	3.46475E-08
91894	NM_080659	C11orf52	chromosome 11 open reading frame 52	-2.076392	0.013206842
256691	NM_001347990,NM_153267,NR_125850	MAMDC2	MAM domain containing 2	-2.080711	1.30343E-11
619279	NM_001033723	ZNF704	zinc finger protein 704	-2.081680	1.88639E-05
25961	NM_001283014,NM_001283015,NM_001283016,NM_001283017,NM_001	NUDT13	nudix hydrolase 13	-2.082047	0.00046424

	283019,NM_015901,NR_104264				
50640	NM_001256007,NM_001256008,NM_001256009,NM_001256010,NM_001256011,NM_015723	PNPLA8	patatin like phospholipase domain containing 8	-2.082214	2.32654E-08
8705	NM_003782	B3GALT4	beta-1,3-galactosyltransferase 4	-2.082632	2.32654E-08
4929	NM_006186	NR4A2	nuclear receptor subfamily 4 group A member 2	-2.084025	2.49302E-06
2524	NM_000511,NM_001097638	FUT2	fucosyltransferase 2	-2.084393	5.62897E-10
256302	NM_152914	NATD1	N-acetyltransferase domain containing 1	-2.084716	0.000525885
3955	NM_001040167,NM_001040168,NM_001166355,NM_002304	LFNG	LFNG O-fucosylpeptide 3-beta-N-acetylglucosaminyltransferase	-2.091160	3.78797E-25
8269	NM_003492	TMEM187	transmembrane protein 187	-2.096133	5.44933E-09
65983	NM_001146319,NM_001146320,NM_001146321,NM_001146322,NM_001349541,NM_001349542,NM_001349543,NM_001349544,NM_023927,NR_146195,NR_146196,NR_146197	GRAMD2B	GRAM domain containing 2B	-2.099456	1.30459E-11
11320	NM_001160154,NM_012214	MGAT4A	mannosyl (alpha-1,3-)-glycoprotein beta-1,4-N-acetylglucosaminyltransferase, isozyme A	-2.101867	0.01962194

55893	NM_018660	ZNF395	zinc finger protein 395	-2.103568	3.07535E-14
2628	NM_001321015,NM_001482	GATM	glycine amidinotransferase	-2.105119	0.042145798
100130872	NR_024569	LOC100130872	uncharacterized LOC100130872	-2.108729	0.03061536
101928068	NR_111907	LOC101928068	uncharacterized LOC101928068	-2.109143	0.011394672
23522	NM_001256468,NM_001256469,NM_012330	KAT6B	lysine acetyltransferase 6B	-2.110533	3.12582E-28
93589	NM_172364	CACNA2D4	calcium voltage-gated channel auxiliary subunit alpha2delta 4	-2.112354	0.006075623
100287896	NM_001319240	LOC100287896	uncharacterized LOC100287896	-2.112767	8.07578E-09
57822	NM_001195010,NM_021180,NM_198173,NM_198174	GRHL3	grainyhead like transcription factor 3	-2.115761	5.0653E-11
11174	NM_197941,NR_135689	ADAMTS6	ADAM metallopeptidase with thrombospondin type 1 motif 6	-2.119841	3.02639E-20
219902	NM_001198670,NM_001198671,NM_001198672,NM_001198673,NM_001198674,NM_001198675,NM_174926	TMEM136	transmembrane protein 136	-2.120334	0.000510852
148418	NM_001010971,NM_001134663,NM_001134664	SAMD13	sterile alpha motif domain containing 13	-2.124038	0.013583588
11074	NM_007028,NR_134870,NR_134871	TRIM31	tripartite motif containing 31	-2.127565	2.08694E-48

55812	NM_001040428,NM_018418	SPATA7	spermatogenesis associated 7	-2.127687	0.001101027
63920	NM_001303251,NM_022090	ZBED8	zinc finger BED-type containing 8	-2.128422	2.98134E-07
2624	NM_001145661,NM_001145662,NM_032638	GATA2	GATA binding protein 2	-2.129013	5.55213E-46
64283	NM_001080479,NM_001177693,NM_001244364	ARHGEF28	Rho guanine nucleotide exchange factor 28	-2.129389	6.13691E-31
8322	NM_012193	FZD4	frizzled class receptor 4	-2.132271	3.56931E-05
283460	NR_024345	HNF1A-AS1	HNF1A antisense RNA 1	-2.133019	0.000226686
2065	NM_001005915,NM_001982	ERBB3	erb-b2 receptor tyrosine kinase 3	-2.140027	1.18815E-43
56256	NM_019605	SERTAD4	SERTA domain containing 4	-2.147460	0.001041684
23531	NM_012329	MMD	monocyte to macrophage differentiation associated	-2.153879	3.81582E-16
389136	NM_001320493,NM_001320494,NM_016206	VGLL3	vestigial like family member 3	-2.160728	3.87553E-05
283375	NM_001135195,NM_173596	SLC39A5	solute carrier family 39 member 5	-2.162040	0.00039528
51091	NM_016955	SEPSECS	Sep (O-phosphoserine) tRNA:Sec (selenocysteine) tRNA synthase	-2.167899	3.59988E-14
51646	NM_001127399,NM_001127400,NM_001127401,	YPEL5	yippee like 5	-2.168425	3.80066E-27

	NM_016061				
100271722	NR_027036	LINC00899	long intergenic non-protein coding RNA 899	-2.175730	0.002478481
100507410	NR_040018,NR_040019	C1QTNF1-AS1	C1QTNF1 antisense RNA 1	-2.178109	0.02584857
9886	NM_001242359,NM_001350902,NM_001350903, NM_001350904,NM_001350905,NM_001350906, NM_001350907,NM_001350908,NM_001350909, NM_001350910,NM_001350911,NM_014836	RHOBTB1	Rho related BTB domain containing 1	-2.180782	0.000215245
285512	NR_002806	FAM13A-AS1	FAM13A antisense RNA 1	-2.181430	0.005921307
114907	NM_001242463,NM_058229,NM_148177	FBXO32	F-box protein 32	-2.184331	2.48497E-13
9630	NM_004297	GNA14	G protein subunit alpha 14	-2.187301	1.19399E-05
196883	NM_001198568,NM_001198592,NM_139247	ADCY4	adenylate cyclase 4	-2.200861	0.011996594
80318	NM_001135953,NM_025211	GKAP1	G kinase anchoring protein 1	-2.201255	0.001247979
79845	NM_024787	RNF122	ring finger protein 122	-2.202172	0.000791241
283487	NR_027701	LINC00346	long intergenic non-protein coding RNA 346	-2.202225	6.88909E-17
3174	NM_001330561,NM_004133	HNF4G	hepatocyte nuclear factor 4 gamma	-2.204547	0.001339568

149076	NM_152493	ZNF362	zinc finger protein 362	-2.206522	0.012235337
26150	NM_015653	RIBC2	RIB43A domain with coiled-coils 2	-2.210808	8.65237E-06
6821	NM_000456,NM_001032386,NM_001032387	SUOX	sulfite oxidase	-2.214526	5.28456E-20
28984	NM_014059	RGCC	regulator of cell cycle	-2.227335	2.97294E-17
64599	NM_022574	GIGYF1	GRB10 interacting GYF protein 1	-2.228137	1.18102E-49
220972	NM_001002266,NM_001282866,NM_145021	MARCH8	membrane associated ring-CH-type finger 8	-2.228404	2.25163E-23
127733	NM_152376	UBXN10	UBX domain protein 10	-2.231835	0.000261702
55139	NM_001042410,NM_001282792,NM_018089	ANKZF1	ankyrin repeat and zinc finger domain containing 1	-2.236661	7.41849E-22
134548	NM_175873	SOWAHA	sosondowah ankyrin repeat domain family member A	-2.241501	7.19251E-06
10608	NM_006454	MXD4	MAX dimerization protein 4	-2.241963	3.41665E-16
115294	NM_001286782,NM_001286783,NM_052937	PCMTD1	protein-L-isoaspartate (D-aspartate) O-methyltransferase domain containing 1	-2.244913	7.61926E-20
151354	NM_145175,NR_144632	FAM84A	family with sequence similarity 84 member A	-2.245149	0.038237108
204962	NM_001130058,NM_001320283,NM_001320285,NM_001320287,NM_152697	SLC44A5	solute carrier family 44 member 5	-2.249465	0.000554533
6666	NM_006943	SOX12	SRY-box 12	-2.249658	5.4238E-10

400604	NR_038458	TOB1-AS1	TOB1 antisense RNA 1	-2.256836	0.017537788
100124700	NR_003716,NR_047517,<br>NR_047518	HOTAIR	HOX transcript antisense RNA	-2.257685	5.06804E-16
284390	NM_001012753	ZNF763	zinc finger protein 763	-2.260494	0.013317031
570	NM_001127610,NM_001<br>701	BAAT	bile acid-CoA:amino acid N-acyltransferase	-2.260856	0.033326379
57730	NM_025190	ANKRD36B	ankyrin repeat domain 36B	-2.264712	1.07905E-05
102724312	NR_125994,NR_125995,<br>NR_125996	LINC01770	long intergenic non-protein coding RNA 1770	-2.265327	0.014941503
54756	NM_001318864,NM_017<br>563	IL17RD	interleukin 17 receptor D	-2.267450	0.001669179
79960	NM_001287437,NM_001<br>287439,NM_001287440,<br>NM_001287441,NM_001<br>287442,NM_001287443,<br>NM_024900,NM_199320	JADE1	jade family PHD finger 1	-2.269989	2.9427E-20
352954	NM_178831,NR_028038,<br>NR_028039,NR_028040	GATS	GATS, stromal antigen 3 opposite strand	-2.270613	7.88552E-07
80325	NM_032548,NM_172027<br>,NR_033429	ABTB1	ankyrin repeat and BTB domain containing 1	-2.271957	7.75595E-13
114991	NM_001318040,NM_001<br>318041,NM_001318042,<br>NM_133374	ZNF618	zinc finger protein 618	-2.277017	0.030423106
101927992	NR_110167,NR_110168	LINC01213	long intergenic non-protein coding RNA 1213	-2.278970	0.047916026

4322	NM_002427	MMP13	matrix metallopeptidase 13	-2.279576	0.004454246
133746	NM_152405	JMY	junction mediating and regulatory protein, p53 cofactor	-2.280108	2.06007E-18
57161	NM_021255	PELI2	pellino E3 ubiquitin protein ligase family member 2	-2.280483	4.03992E-11
3434	NM_001270927,NM_001270928,NM_001270929,NM_001270930,NM_001548	IFIT1	interferon induced protein with tetratricopeptide repeats 1	-2.290621	0.001362062
401303	NR_023382	ZNF815P	zinc finger protein 815, pseudogene	-2.291867	0.023682719
124751	NM_001304947,NM_213597	KRBA2	KRAB-A domain containing 2	-2.293726	0.020057257
100616668	NR_038258,NR_038259	TPTE2P5	transmembrane phosphoinositide 3-phosphatase and tensin homolog 2 pseudogene 5	-2.309969	0.00062962
402415	NM_212559	XKRX	XK related, X-linked	-2.310187	3.93569E-07
4215	NM_001330431,NM_002401,NM_203351	MAP3K3	mitogen-activated protein kinase kinase kinase 3	-2.310507	1.82084E-30
84733	NM_005189,NM_032647	CBX2	chromobox 2	-2.311330	1.7886E-15
118738	NM_001346932,NM_001346933,NM_001346934,NM_001346935,NM_001346936,NM_153034	ZNF488	zinc finger protein 488	-2.313317	8.10866E-19
8348	NM_003527	HIST1H2BO	histone cluster 1 H2B family member o	-2.316296	0.046459541

6519	NM_000341	SLC3A1	solute carrier family 3 member 1	-2.321827	0.001054527
29125	NM_001142946,NM_001329958,NR_138249	C11orf21	chromosome 11 open reading frame 21	-2.331612	1.17105E-05
10902	NM_001164326,NM_001300961,NM_001300962,NM_001300966,NM_006696,NM_139199	BRD8	bromodomain containing 8	-2.333195	5.21466E-54
102724668	NR_132360	DPY19L1P2	DPY19L1 pseudogene 2	-2.333789	0.002143857
400986	NM_001310154	ANKRD36C	ankyrin repeat domain 36C	-2.338402	5.29251E-09
8876	NM_004666	VNN1	vanin 1	-2.340783	7.83308E-14
10370	NM_001168388,NM_001168389,NM_006079	CITED2	Cbp/p300 interacting transactivator with Glu/Asp rich carboxy-terminal domain 2	-2.341017	4.08201E-07
64714	NM_006849	PDIA2	protein disulfide isomerase family A member 2	-2.348433	0.040182737
140738	NM_183240	TMEM37	transmembrane protein 37	-2.359163	5.68729E-05
147011	NM_001304949,NM_001304951,NM_001304952,NM_001304953,NM_001304954,NM_152465	PROCA1	protein interacting with cyclin A1	-2.368852	0.013775294
115330	NM_001303473,NM_001303474,NM_138445	GPR146	G protein-coupled receptor 146	-2.372495	0.001718771
440925	NR_027433	LINC01124	long intergenic non-protein coding RNA 1124	-2.376814	0.033080961
100528020	NM_001258400	FAM187A	family with sequence similarity 187 member A	-2.379458	0.009543882
83463	NM_001142935,NM_031	MXD3	MAX dimerization protein 3	-2.389761	1.36225E-12

	300				
91461	NM_138370	PKDCC	protein kinase domain containing, cytoplasmic	-2.401468	8.11275E-27
154043	NM_173515	CNKSR3	CNKSR family member 3	-2.402465	0.043080157
84961	NM_001184906,NM_032875	FBXL20	F-box and leucine rich repeat protein 20	-2.403562	1.8629E-14
93	NM_001106	ACVR2B	activin A receptor type 2B	-2.409027	3.55837E-13
54943	NM_001040192,NM_001320746,NM_017833	DNAJC28	DnaJ heat shock protein family (Hsp40) member C28	-2.412128	0.006352032
143879	NM_001330359,NM_152433,NM_198439	KBTBD3	kelch repeat and BTB domain containing 3	-2.417332	9.41365E-10
9771	NM_012294	RAPGEF5	Rap guanine nucleotide exchange factor 5	-2.417745	8.66078E-12
55315	NM_001174098,NM_018344,NR_033413,NR_033414	SLC29A3	solute carrier family 29 member 3	-2.418928	3.99723E-18
154743	NM_152556	BMT2	base methyltransferase of 25S rRNA 2 homolog	-2.426491	0.014408993
7754	NR_002722,NR_024553	ZNF204P	zinc finger protein 204, pseudogene	-2.426797	0.010593222
55088	NM_001321829,NM_018017,NM_153249,NR_135815	CCDC186	coiled-coil domain containing 186	-2.443778	0.000349256
100130111	NR_135221	LOC100130111	uncharacterized LOC100130111	-2.449293	0.007216856
9991	NM_001163788,NM_001163790,NM_001244896, NM_001244897,NM_001	PTBP3	polypyrimidine tract binding protein 3	-2.453560	0.000131965

	244898,NM_005156				
9514	NM_001318103,NM_001318104,NM_001318105,NM_001318106,NM_001318107,NM_001318108,NM_001318109,NM_001318110,NM_001318111,NM_001318112,NM_001318113,NM_001318114,NM_001318115,NM_001318116,NM_004861	GAL3ST1	galactose-3-O-sulfotransferase 1	-2.458083	0.017858932
9403	NM_004261,NM_203341,NR_144512,NR_144513	SELENOF	selenoprotein F	-2.466224	7.5777E-15
147912	NM_175875	SIX5	SIX homeobox 5	-2.467148	0.025067505
8329	NM_003509	HIST1H2AI	histone cluster 1 H2A family member i	-2.471876	7.18526E-52
135932	NM_001242773,NM_001242774,NM_001242775,NM_001282876,NM_001282877,NM_153345,NR_040003,NR_104250,NR_104251,NR_104252,NR_104253,NR_104254	TMEM139	transmembrane protein 139	-2.471911	5.6762E-07
81788	NM_030952	NUAK2	NUAK family kinase 2	-2.474398	9.69344E-05
59084	NM_001290072,NM_001290073,NM_021572	ENPP5	ectonucleotide pyrophosphatase/phosphodiesterase 5 (putative)	-2.484850	8.75401E-05
167691	NM_001122769,NM_181714	LCA5	LCA5, lebercilin	-2.495208	4.07752E-33

30061	NM_014585	SLC40A1	solute carrier family 40 member 1	-2.498155	5.87674E-06
8654	NM_001083,NM_033430,NM_033437	PDE5A	phosphodiesterase 5A	-2.502272	0.000932274
284618	NR_145424,NR_145425,NR_145426	RUSC1-AS1	RUSC1 antisense RNA 1	-2.524967	0.000932274
79946	NM_024886	C10orf95	chromosome 10 open reading frame 95	-2.534914	0.000114352
25928	NM_015464	SOSTDC1	sclerostin domain containing 1	-2.539003	5.86385E-26
126272	NM_152361	EID2B	EP300 interacting inhibitor of differentiation 2B	-2.549883	1.11269E-05
79849	NM_001168468,NM_024791,NR_033122	PDZD3	PDZ domain containing 3	-2.554129	0.000967466
11119	NM_001145008,NM_001145009,NM_007048,NM_194441	BTN3A1	butyrophilin subfamily 3 member A1	-2.554760	2.69389E-07
286336	NM_033387	FAM78A	family with sequence similarity 78 member A	-2.568446	0.00158276
100506810	NR_038856	LINC01132	long intergenic non-protein coding RNA 1132	-2.570257	0.000463126
143686	NM_001271594,NM_144665	SESN3	sestrin 3	-2.592892	1.33262E-09
1028	NM_000076,NM_001122630,NM_001122631	CDKN1C	cyclin dependent kinase inhibitor 1C	-2.593241	1.9419E-07
3223	NM_004503,NM_153693	HOXC6	homeobox C6	-2.595161	1.24019E-11
84787	NM_032701	KMT5C	lysine methyltransferase 5C	-2.598620	4.42057E-17
8660	NM_003749	IRS2	insulin receptor substrate 2	-2.605179	1.28541E-32

146330	NM_153350	FBXL16	F-box and leucine rich repeat protein 16	-2.606690	0.026971021
1740	NM_001142699,NM_001142700,NM_001142702,NM_001206769,NM_001300983,NM_001351274,NM_001351275,NM_001351276,NM_001364	DLG2	discs large MAGUK scaffold protein 2	-2.607109	0.018118117
55701	NM_001278529,NM_001278530,NM_018071	ARHGEF40	Rho guanine nucleotide exchange factor 40	-2.608894	8.66911E-14
2263	NM_000141,NM_001144913,NM_001144914,NM_001144915,NM_001144916,NM_001144917,NM_001144918,NM_001144919,NM_001320654,NM_001320658,NM_022970,NM_023029,NR_073009	FGFR2	fibroblast growth factor receptor 2	-2.613216	9.81768E-39
4661	NM_004535	MYT1	myelin transcription factor 1	-2.615672	1.89428E-08
130574	NM_001195685,NM_194317	LYPD6	LY6/PLAUR domain containing 6	-2.619840	0.000347725
1942	NM_004428,NM_182685	EFNA1	ephrin A1	-2.621528	8.00619E-17
23401	NM_012083	FRAT2	FRAT2, WNT signaling pathway regulator	-2.633235	3.06363E-37
80054	NR_026887	CEBPA-AS1	CEBPA antisense RNA 1 (head to head)	-2.638252	0.006751532
389384	NM_001010903	C6orf222	chromosome 6 open reading frame 222	-2.649366	0.011335988
3760	NM_001260508,NM_001	KCNJ3	potassium voltage-gated channel subfamily J	-2.667285	3.01243E-05

	260509,NM_001260510, NM_002239		member 3		
284358	NM_001130915,NM_001297753,NM_182574	MAMSTR	MEF2 activating motif and SAP domain containing transcriptional regulator	-2.672769	0.026086725
65986	NM_001105539,NM_001277145,NM_023929	ZBTB10	zinc finger and BTB domain containing 10	-2.673234	7.60627E-07
219699	NM_001244889,NM_170744	UNC5B	unc-5 netrin receptor B	-2.673535	0.03534963
29799	NM_013313,NR_130910	YPEL1	yippee like 1	-2.673668	0.007879739
283991	NM_182565	UBALD2	UBA like domain containing 2	-2.676700	1.09832E-42
10891	NM_001330751,NM_001330752,NM_001330753, NM_013261	PPARGC1A	PPARG coactivator 1 alpha	-2.688221	0.015216456
717	NM_000063,NM_001145903,NM_001178063,NM_001282457,NM_001282458,NM_001282459	C2	complement C2	-2.688533	0.042622263
2535	NM_001466	FZD2	frizzled class receptor 2	-2.697310	0.022482127
27244	NM_001199933,NM_001199934,NM_014454	SESN1	sestrin 1	-2.702948	6.68626E-32
2444	NM_002031	FRK	fyn related Src family tyrosine kinase	-2.703613	5.55622E-08
84206	NM_032246	MEX3B	mex-3 RNA binding family member B	-2.708792	2.44099E-05
474	NM_005172	ATOH1	atonal bHLH transcription factor 1	-2.714553	0.048335564

125170	NM_001144900,NM_139162,NM_148886	MIEF2	mitochondrial elongation factor 2	-2.728930	2.78143E-22
2043	NM_001304536,NM_001304537,NM_004438	EPHA4	EPH receptor A4	-2.730931	0.001427588
55766	NM_177925,NR_027716	H2AFJ	H2A histone family member J	-2.735505	0.009835064
114819	NR_023386	CROCCP3	ciliary rootlet coiled-coil, rootletin pseudogene 3	-2.736574	0.010351538
348110	NM_001282380,NM_182616	ARPIN	actin related protein 2/3 complex inhibitor	-2.747527	0.00885157
23177	NM_001319100,NM_001319101,NM_015147,NR_134966	CEP68	centrosomal protein 68	-2.752612	1.47993E-15
84666	NM_032579	RETNLB	resistin like beta	-2.758334	0.029137107
3718	NM_000215	JAK3	Janus kinase 3	-2.761517	0.006924549
8408	NM_003565	ULK1	unc-51 like autophagy activating kinase 1	-2.762410	2.56789E-26
65249	NM_023072	ZSWIM4	zinc finger SWIM-type containing 4	-2.773544	2.12668E-11
55281	NM_018295	TMEM140	transmembrane protein 140	-2.788289	6.88356E-07
3660	NM_002199	IRF2	interferon regulatory factor 2	-2.789128	2.7927E-24
126375	NM_175872	ZNF792	zinc finger protein 792	-2.798913	1.49879E-21
79818	NM_024762	ZNF552	zinc finger protein 552	-2.799453	1.86956E-54
56670	NM_033050	SUCNR1	succinate receptor 1	-2.827494	0.00690467
165679	NM_001040100,NM_001	SPTSSB	serine palmitoyltransferase small subunit B	-2.831953	7.01767E-11

	320679				
2306	NM_004474	FOXD2	forkhead box D2	-2.843108	0.000685896
4207	NM_005919,NR_027307, NR_027308	BORCS8-MEF2B	BORCS8-MEF2B readthrough	-2.850215	0.00114767
3268	NM_006076	AGFG2	ArfGAP with FG repeats 2	-2.860336	3.86808E-40
374907	NM_198540	B3GNT8	UDP-GlcNAc:betaGal beta-1,3-N-acetylglucosaminyltransferase 8	-2.919425	0.000510872
80853	NM_030647	KDM7A	lysine demethylase 7A	-2.934733	1.00551E-14
80352	NM_025236,NM_170769	RNF39	ring finger protein 39	-2.942031	1.80397E-14
19	NM_005502	ABCA1	ATP binding cassette subfamily A member 1	-2.955145	5.42155E-10
9881	NM_001329998,NM_014831	TRANK1	tetratricopeptide repeat and ankyrin repeat containing 1	-2.961427	3.22205E-11
23024	NM_001303139,NM_001303140,NM_001303141, NM_001303142,NM_015009	PDZRN3	PDZ domain containing ring finger 3	-2.972782	0.010393088
100506286	NR_135594	TBC1D8-AS1	TBC1 domain family member 8 antisense RNA 1	-2.974644	0.044748603
10420	NM_001320800,NM_007170	TESK2	testis-specific kinase 2	-2.984387	7.63007E-13
5727	NM_000264,NM_001083602,NM_001083603,NM_001083604,NM_001083605,NM_001083606,NM	PTCH1	patched 1	-2.986625	4.0569E-29

	_001083607				
85442	NM_001347864,NM_001347865,NM_001347866,NM_152643	KNDC1	kinase non-catalytic C-lobe domain containing 1	-2.992794	0.044369232
285513	NM_198281	GPRIN3	GPRIN family member 3	-3.000192	0.000354113
51063	NM_015916,NR_024552,NR_046344	CALHM2	calcium homeostasis modulator 2	-3.037424	7.2267E-13
7049	NM_001195683,NM_001195684,NM_003243,NR_036634	TGFBR3	transforming growth factor beta receptor 3	-3.067500	2.15318E-24
6097	NM_001001523,NM_005060	RORC	RAR related orphan receptor C	-3.075268	0.002841719
100534593	NR_037945	STX16-NPEPL1	STX16-NPEPL1 readthrough (NMD candidate)	-3.076024	0.043360316
10628	NM_001313972,NM_006472	TXNIP	thioredoxin interacting protein	-3.084861	1.0095E-70
57507	NM_020747	ZNF608	zinc finger protein 608	-3.090785	5.36674E-14
375287	NM_198557	RBM43	RNA binding motif protein 43	-3.091632	1.88485E-09
140893	NM_080833	RBBP8NL	RBBP8 N-terminal like	-3.093956	1.47184E-17
9754	NM_001142503,NM_001142504,NM_014725	STARD8	StAR related lipid transfer domain containing 8	-3.106016	0.001463871
100873955	NR_046827	LNX1-AS2	LNX1 antisense RNA 2	-3.123020	0.035858437
440028	NR_033972	LOC440028	uncharacterized LOC440028	-3.207169	0.01805221

145788	NM_001198784	C15orf65	chromosome 15 open reading frame 65	-3.212430	8.761E-10
10008	NM_005472	KCNE3	potassium voltage-gated channel subfamily E regulatory subunit 3	-3.219440	1.85775E-62
151050	NM_001307976,NM_152519	KANSL1L	KAT8 regulatory NSL complex subunit 1 like	-3.234456	3.44694E-26
153222	NM_001168393,NM_001168394,NM_153607	CREBRF	CREB3 regulatory factor	-3.239445	3.50897E-24
7294	NM_003328	TXK	TXK tyrosine kinase	-3.240078	2.3484E-05
84708	NM_001126328,NM_032622	LNX1	ligand of numb-protein X 1	-3.290444	7.94185E-17
171523	NR_144551	CYP2T1P	cytochrome P450 family 2 subfamily T member 1, pseudogene	-3.294667	0.001595813
57687	NM_020927	VAT1L	vesicle amine transport 1 like	-3.296776	0.025507207
101926935	NR_110001	LOC101926935	uncharacterized LOC101926935	-3.299285	0.034718238
255488	NM_182757	RNF144B	ring finger protein 144B	-3.300364	4.4358E-17
25822	NM_001135004,NM_001135005,NM_001349723,NM_001349724,NM_001349725,NM_012266	DNAJB5	DnaJ heat shock protein family (Hsp40) member B5	-3.312979	0.000758635
53349	NM_001281734,NM_001281735,NM_021260,NM_178441	ZFYVE1	zinc finger FYVE-type containing 1	-3.313566	3.0934E-14
54823	NM_001105518,NM_017	SWT1	SWT1, RNA endoribonuclease homolog	-3.319670	1.11297E-14

	673				
1944	NM_004952	EFNA3	ephrin A3	-3.325570	5.31672E-08
4857	NM_002515,NM_006489,NM_006491	NOVA1	NOVA alternative splicing regulator 1	-3.339821	0.000200658
55503	NM_018646	TRPV6	transient receptor potential cation channel subfamily V member 6	-3.340315	0.005898967
4013	NM_001130142,NM_014622,NM_198315	VWA5A	von Willebrand factor A domain containing 5A	-3.365078	0.001643032
868	NM_001321786,NM_001321788,NM_001321789,NM_001321790,NM_001321791,NM_001321793,NM_001321794,NM_001321795,NM_001321796,NM_001321797,NM_001321798,NM_001321799,NM_001321806,NM_001321807,NM_001321808,NM_001321811,NM_001321813,NM_001321816,NM_001321820,NM_001321822,NM_170662,NR_135806,NR_135807,NR_135808,NR_135809,NR_135810,NR_135811,NR_135812	CBLB	Cbl proto-oncogene B	-3.390710	1.35928E-30
9469	NM_004273	CHST3	carbohydrate sulfotransferase 3	-3.410704	3.83179E-25
5013	NM_001199770,NM_014	OTX1	orthodenticle homeobox 1	-3.411997	6.94002E-21

	562,NR_130153				
283209	NM_173582	PGM2L1	phosphoglucomutase 2 like 1	-3.445653	3.16046E-27
153020	NM_001300735,NM_001300736,NM_152545	RASGEF1B	RasGEF domain family member 1B	-3.472613	0.000788974
105371267	NR_136518,NR_147075	LOC105371267	p53-regulated lncRNA 1	-3.492232	0.002132294
5801	NM_001207015,NM_001207016,NM_002849,NM_130846,NR_073474	PTPRR	protein tyrosine phosphatase, receptor type R	-3.510118	2.05113E-09
79170	NM_024320	PRR15L	proline rich 15 like	-3.573185	5.90928E-35
399821	NR_033847	FLJ37035	uncharacterized LOC399821	-3.576616	0.004478795
107984921	NR_147210,NR_147211	LINC01772	long intergenic non-protein coding RNA 1772	-3.583718	0.013454015
54567	NM_019074	DLL4	delta like canonical Notch ligand 4	-3.587507	3.276E-05
344657	NM_001080460	LRRIQ4	leucine rich repeats and IQ motif containing 4	-3.615424	0.03454247
63982	NM_001313726,NM_001313727,NM_031418	ANO3	anoctamin 3	-3.642441	1.04356E-06
10194	NM_001308210,NM_005786	TSHZ1	teashirt zinc finger homeobox 1	-3.672204	1.98876E-53
1375	NM_001145134,NM_001145135,NM_001145137,NM_004377,NM_152245,NM_152246	CPT1B	carnitine palmitoyltransferase 1B	-3.708857	0.00017869
152926	NM_152542	PPM1K	protein phosphatase, Mg2+/Mn2+ dependent 1K	-3.716329	6.94867E-18

604	NM_001130845,NM_001134738,NM_001706	BCL6	B-cell CLL/lymphoma 6	-3.717538	6.06496E-45
389058	NM_001003845	SP5	Sp5 transcription factor	-3.720096	6.4749E-10
389072	NM_001080475	PLEKHM3	pleckstrin homology domain containing M3	-3.739233	2.41548E-34
6792	NM_001037343,NM_001323289,NM_003159	CDKL5	cyclin dependent kinase like 5	-3.765903	0.047342476
5453	NM_002699	POU3F1	POU class 3 homeobox 1	-3.769029	2.09873E-07
56204	NM_001286495,NM_019600,NR_104457	FAM214A	family with sequence similarity 214 member A	-3.773881	6.35323E-18
83719	NM_001145524,NM_031477	YPEL3	yippee like 3	-3.822659	5.60645E-25
100129722	NR_038389	STPG3-AS1	STPG3 antisense RNA 1	-3.861565	0.013701609
8431	NM_021969	NR0B2	nuclear receptor subfamily 0 group B member 2	-3.867591	3.99944E-07
8000	NM_005672,NR_033343	PSCA	prostate stem cell antigen	-4.014668	1.61752E-23
1906	NM_001168319,NM_001955	EDN1	endothelin 1	-4.087919	4.91257E-76
57653	NR_036527,NR_036528,NR_036529	LOC100499484-C9ORF174	LOC100499484-C9orf174 readthrough	-4.136917	0.001180688
388403	NM_001005404	YPEL2	yippee like 2	-4.174225	6.67509E-27
8360	NM_003539	HIST1H4D	histone cluster 1 H4 family member d	-4.208971	0.005652673
84189	NM_032229	SLITRK6	SLIT and NTRK like family member 6	-4.209327	1.87E-65

353355	NM_001207005,NM_001330529,NM_181756	ZNF233	zinc finger protein 233	-4.309364	2.65129E-07
8743	NM_001190942,NM_001190943,NM_003810,NR_033994	TNFSF10	TNF superfamily member 10	-4.366161	1.56307E-33
4306	NM_000901,NM_001166104	NR3C2	nuclear receptor subfamily 3 group C member 2	-4.445017	0.00017092
23285	NM_015237	KIAA1107	KIAA1107	-4.502848	5.86693E-10
5624	NM_000312	PROC	protein C, inactivator of coagulation factors Va and VIIIa	-4.637431	0.002094652
83875	NM_001037290,NM_001256397,NM_001256398,NM_001256400,NM_031938	BCO2	beta-carotene oxygenase 2	-4.660507	0.009911772
10957	NM_006813	PNRC1	proline rich nuclear receptor coactivator 1	-4.690316	3.28226E-29
4602	NM_001130172,NM_001130173,NM_001161656,NM_001161657,NM_001161658,NM_001161659,NM_001161660,NM_005375,NR_134958,NR_134959,NR_134960,NR_134961,NR_134962,NR_134963,NR_134964,NR_134965	MYB	MYB proto-oncogene, transcription factor	-4.710209	1.42099E-37
7832	NM_006763	BTG2	BTG anti-proliferation factor 2	-4.711613	2.01362E-70

2199	NM_001004019,NM_001165035,NM_001998	FBLN2	fibulin 2	-4.761109	0.004774162
121268	NM_001303126,NM_144593,NR_130123	RHEBL1	Ras homolog enriched in brain like 1	-4.782962	1.48394E-10
113791	NM_001135911,NM_052880	PIK3IP1	phosphoinositide-3-kinase interacting protein 1	-4.843752	0.000123644
333929	NM_178310	SNAI3	snail family transcriptional repressor 3	-4.891289	0.000180683
149428	NM_001159642,NM_138278	BNIPL	BCL2 interacting protein like	-4.900448	0.007042335
9953	NM_006041,NR_130138	HS3ST3B1	heparan sulfate-glucosamine 3-sulfotransferase 3B1	-5.141548	0.046004343
286204	NM_173689,NR_104603	CRB2	crumbs 2, cell polarity complex component	-5.290100	1.55933E-08
83992	NM_033427	CTTNBP2	cortactin binding protein 2	-5.337789	3.14338E-10
5166	NM_002612	PDK4	pyruvate dehydrogenase kinase 4	-5.364554	1.22953E-19
26959	NM_001244262,NM_012257	HBP1	HMG-box transcription factor 1	-5.770567	6.7953E-99
10023	NM_005479	FRAT1	FRAT1, WNT signaling pathway regulator	-5.975201	9.2339E-12
84159	NM_001244638,NM_032199	ARID5B	AT-rich interaction domain 5B	-6.554911	5.2763E-128
729533	NM_001123168,NM_001317901,NR_134239	FAM72A	family with sequence similarity 72 member A	-6.951512	0.01128773
54800	NM_001349413,NM_001349414,NM_001349415,	KLHL24	kelch like family member 24	-7.059956	4.66445E-10

	NM_001349416,NM_001349417,NM_001349418,NM_001349419,NM_001349420,NM_001349421,NM_001349422,NM_001349423,NM_001349424,NM_001349425,NM_001349426,NM_001349427,NM_001349428,NM_001349429,NM_017644,NR_146169,NR_146170				
2886	NM_001030002,NM_001242442,NM_001242443,NM_001330207,NM_005310	GRB7	growth factor receptor bound protein 7	-7.513283	4.38723E-94
83478	NM_001025616,NM_001042669,NM_001287805,NM_001346093,NM_031305	ARHGAP24	Rho GTPase activating protein 24	-8.206625	1.72787E-05
94241	NM_001135733,NM_033285	TP53INP1	tumor protein p53 inducible nuclear protein 1	-8.590492	2.91061E-19
729438	NM_001145064	CASTOR2	cytosolic arginine sensor for mTORC1 subunit 2	-27.338462	1.83591E-13
388692	NM_001013644	LOC388692	uncharacterized LOC388692	-33.954716	0.022983273

Table S5 List of differentially expressed genes between CotG-p75-stimulated and wild-type spore-stimulated HT-29 cells

Gene ID	Transcript ID	Gene Symbol	Description	Fold change (G75/WT)	Raw *p*-val
1543	NM_000499,NM_001319216,NM_001319217	CYP1A1	cytochrome P450 family 1 subfamily A member 1	251.652220	5.68413E-96
101060321	NM_001291462,NM_001291462_dup1,NM_001291462_dup2,NM_001291462_dup3	TBC1D3G	TBC1 domain family member 3G	142.323217	0.000680907
904	NM_001240,NM_001277842	CCNT1	cyclin T1	32.757921	0.001508658
121274	NM_001172681,NM_001172682,NM_152320	ZNF641	zinc finger protein 641	14.578613	5.48174E-10
1545	NM_000104	CYP1B1	cytochrome P450 family 1 subfamily B member 1	8.934501	0
149041	NM_001300850,NM_001300851,NM_001300852, NM_172071	RC3H1	ring finger and CCCH-type domains 1	8.755187	0.008819437
100131234	NR_040073	MIR181A1HG	MIR181A1 host gene	7.759207	0.014822303
220	NM_000693,NM_001293815	ALDH1A3	aldehyde dehydrogenase 1 family member A3	7.523263	5.7102E-308
6699	NM_003125	SPRR1B	small proline rich protein 1B	6.687715	4.32868E-24
51702	NM_016233	PADI3	peptidyl arginine deiminase 3	6.395291	1.83103E-28
168451	NM_001130475,NM_001287598,NM_001287599, NM_001287601,NM_182529	THAP5	THAP domain containing 5	6.164148	0.016686814
6707	NM_001097589,NM_005416	SPRR3	small proline rich protein 3	6.118204	6.37206E-27

114882	NM_001003712,NM_001319652,NM_001319653,NM_001319655,NM_020841	OSBPL8	oxysterol binding protein like 8	6.107394	0.005820532
6317	NM_006919	SERPINB3	serpin family B member 3	6.046358	0.00056539
375791	NM_199001	CYSRT1	cysteine rich tail 1	5.802612	1.36895E-24
346389	NM_182762	MACC1	MACC1, MET transcriptional regulator	5.756295	0.004581468
8120	NM_001278511,NM_001278512,NM_001348440,NM_001348441,NM_004644	AP3B2	adaptor related protein complex 3 beta 2 subunit	5.748581	6.12125E-06
6398	NM_003004	SECTM1	secreted and transmembrane 1	5.435386	2.84165E-13
29114	NM_001008272,NM_001008273,NM_013259	TAGLN3	transgelin 3	5.369125	5.66741E-12
164312	NM_152611	LRRN4	leucine rich repeat neuronal 4	5.212794	9.66806E-20
84239	NM_032279	ATP13A4	ATPase 13A4	4.940219	0.001652684
2736	NM_005270	GLI2	GLI family zinc finger 2	4.877476	1.96835E-14
79094	NM_001142776,NM_024111	CHAC1	ChaC glutathione specific gamma-glutamylcyclotransferase 1	4.743025	3.64426E-16
347365	NM_198510	ITIH6	inter-alpha-trypsin inhibitor heavy chain family member 6	4.706921	0.007476815
1588	NM_000103,NM_001347248,NM_001347249,NM_001347250,NM_001347251,NM_001347252,NM_001347253,NM_001347254,NM_001347255,NM_001347256,NM_031226	CYP19A1	cytochrome P450 family 19 subfamily A member 1	4.514089	0.004948059
10170	NM_001142270,NM_001	DHRS9	dehydrogenase/reductase 9	4.503366	1.77855E-41

	142271,NM_001289763,NM_199204				
2702	NM_005266,NM_181703	GJA5	gap junction protein alpha 5	4.455071	6.15392E-28
7133	NM_001066	TNFRSF1B	TNF receptor superfamily member 1B	4.341483	2.24005E-10
85409	NM_001271082,NM_033120	NKD2	naked cuticle homolog 2	3.944249	8.07598E-46
5582	NM_001316329,NM_002739	PRKCG	protein kinase C gamma	3.864862	5.77407E-15
84290	NM_032330	CAPNS2	calpain small subunit 2	3.663600	0.000254949
8771	NM_003823	TNFRSF6B	TNF receptor superfamily member 6b	3.662200	5.36865E-05
221472	NM_173558	FGD2	FYVE, RhoGEF and PH domain containing 2	3.623011	0.003796263
6536	NM_001024845,NM_001261380,NM_001328626,NM_001328627,NM_001328628,NM_001328629,NM_001328630,NM_006934,NM_201649,NR_048548	SLC6A9	solute carrier family 6 member 9	3.612395	1.93522E-05
3038	NM_001199280,NM_005329,NM_138612	HAS3	hyaluronan synthase 3	3.601491	1.43771E-09
254439	NM_001136485	C11orf86	chromosome 11 open reading frame 86	3.560029	4.18317E-06
10896	NM_022375	OCLM	oculomedin	3.449083	0.01149653
255324	NM_001270989,NM_001270990,NM_001270991,NM_001270992,NM_001270993,NR_073114,NR_073117	EPGN	epithelial mitogen	3.417381	0.028726372
100131378	NM_001166692	C11orf91	chromosome 11 open reading frame 91	3.401440	0.000263494

8482	NM_001146029,NM_001146030,NM_003612	SEMA7A	semaphorin 7A (John Milton Hagen blood group)	3.381057	5.68696E-06
54541	NM_019058	DDIT4	DNA damage inducible transcript 4	3.352270	2.92733E-81
285268	NM_001098414,NM_001287245,NM_198484	ZNF621	zinc finger protein 621	3.332213	0.018896531
7433	NM_001251882,NM_001251883,NM_001251884,NM_001251885,NM_004624	VIPR1	vasoactive intestinal peptide receptor 1	3.301784	3.25269E-79
1291	NM_001848	COL6A1	collagen type VI alpha 1 chain	3.236719	0.004570486
3009	NM_005322	HIST1H1B	histone cluster 1 H1 family member b	3.192682	0.024532126
7307	NM_001025203,NM_001025204,NM_006758	U2AF1	U2 small nuclear RNA auxiliary factor 1	3.150412	0.018229073
1026	NM_000389,NM_001220777,NM_001220778,NM_001291549,NM_078467	CDKN1A	cyclin dependent kinase inhibitor 1A	3.117541	1.36729E-90
356	NM_000639,NM_001302746	FASLG	Fas ligand	3.109825	0.014009789
285154	NR_027252	CYP1B1-AS1	CYP1B1 antisense RNA 1	3.109478	2.68426E-08
4856	NM_002514	NOV	nephroblastoma overexpressed	3.087774	0.006320913
9788	NM_001282971,NM_001282974,NM_014751	MTSS1	MTSS1, I-BAR domain containing	3.067953	2.73134E-24
79718	NM_001321193,NM_001321194,NM_001321195,NM_024665	TBL1XR1	transducin beta like 1 X-linked receptor 1	3.047832	0.018662363
400221	NR_039985	FLJ22447	uncharacterized LOC400221	3.046488	0.012697101
2840	NM_001161415,NM_001161416,NM_001161417,	GPR17	G protein-coupled receptor 17	3.027350	2.04266E-05

	NM_005291				
55084	NM_018013	SOBP	sine oculis binding protein homolog	2.886317	0.00767208
3352	NM_000864	HTR1D	5-hydroxytryptamine receptor 1D	2.880084	9.63148E-39
3575	NM_002185,NR_120485	IL7R	interleukin 7 receptor	2.825362	0.017604775
57214	NM_001293298,NM_001293304,NM_018689	CEMIP	cell migration inducing hyaluronan binding protein	2.824266	3.04199E-51
250	NM_001632	ALPP	alkaline phosphatase, placental	2.810857	0.002970607
85495	NR_002312	RPPH1	ribonuclease P RNA component H1	2.788936	0.001490797
5743	NM_000963	PTGS2	prostaglandin-endoperoxide synthase 2	2.769494	4.93081E-55
100499467	NR_036488,NR_137280, NR_137281	LINC00673	long intergenic non-protein coding RNA 673	2.758183	1.43804E-49
3872	NM_000422	KRT17	keratin 17	2.741327	1.18658E-42
9839	NM_001171653,NM_014795,NR_033258	ZEB2	zinc finger E-box binding homeobox 2	2.709612	0.023755334
8862	NM_017413	APLN	apelin	2.709293	0.030804032
400619	NR_033876	LINC00511	long intergenic non-protein coding RNA 511	2.686109	1.85669E-07
9518	NM_004864	GDF15	growth differentiation factor 15	2.677628	5.47262E-45
117157	NM_053282	SH2D1B	SH2 domain containing 1B	2.674360	0.032607663
6698	NM_001199828,NM_005987	SPRR1A	small proline rich protein 1A	2.629925	0.012450711
219855	NM_001145290,NM_198277	SLC37A2	solute carrier family 37 member 2	2.628228	1.86397E-10
8193	NM_001135155,NM_001135156,NM_001289978, NM_004647	DPF1	double PHD fingers 1	2.599998	0.000949037
9536	NM_004878	PTGES	prostaglandin E synthase	2.569392	1.3564E-08

105374177	NR_135546	LINC02029	long intergenic non-protein coding RNA 2029	2.566250	2.37434E-06
3008	NM_005321	HIST1H1E	histone cluster 1 H1 family member e	2.565770	0.01874117
245973	NM_001039362,NM_144583	ATP6V1C2	ATPase H+ transporting V1 subunit C2	2.542487	3.65581E-05
78990	NM_023112	OTUB2	OTU deubiquitinase, ubiquitin aldehyde binding 2	2.535288	7.51788E-16
2069	NM_001432	EREG	epiregulin	2.534662	7.90063E-78
84623	NM_001161707,NM_001301097,NM_032531	KIRREL3	kin of IRRE like 3 (Drosophila)	2.504548	0.000857494
23328	NM_001346505,NM_001346506,NM_001346507,NM_001346508,NM_001346509,NM_015278	SASH1	SAM and SH3 domain containing 1	2.498133	4.04766E-25
266977	NM_025048,NM_153840	ADGRF1	adhesion G protein-coupled receptor F1	2.465793	1.35153E-62
101929125	NR_109859	LINC01730	long intergenic non-protein coding RNA 1730	2.461604	0.037708918
3283	NM_000862,NM_001328615	HSD3B1	hydroxy-delta-5-steroid dehydrogenase, 3 beta- and steroid delta-isomerase 1	2.456860	6.16907E-19
25758	NM_012194	KIAA1549L	KIAA1549 like	2.439854	0.031338418
93429	NR_027003	LOC93429	uncharacterized LOC93429	2.433951	0.000369655
84803	NM_001256421,NM_001256422,NM_032717	GPAT3	glycerol-3-phosphate acyltransferase 3	2.433066	1.09083E-44
54206	NM_018948	ERRFI1	ERBB receptor feedback inhibitor 1	2.425053	3.21918E-70
374	NM_001657,NM_001657_dup1	AREG	amphiregulin	2.422047	1.79058E-63
25907	NM_015444	TMEM158	transmembrane protein 158 (gene/pseudogene)	2.388364	1.99138E-11
5054	NM_000602	SERPINE1	serpin family E member 1	2.384717	3.57682E-08
8061	NM_001300844,NM_001	FOSL1	FOS like 1, AP-1 transcription factor subunit	2.359547	1.59604E-36

	300855,NM_001300856, NM_001300857,NM_005438,NR_125339				
100529207	NR_037714	RAD51L3-RFFL	RAD51L3-RFFL readthrough	2.353991	1.91162E-14
101241892	NR_103844	NPTN-IT1	NPTN intronic transcript 1	2.348173	0.002501013
6004	NM_002928	RGS16	regulator of G protein signaling 16	2.343767	1.08309E-09
1958	NM_001964	EGR1	early growth response 1	2.339189	0.000284156
91663	NM_001020818,NM_001020819,NM_001020820, NM_001020821,NM_001290188,NM_001290189, NM_001290190,NM_001290191,NM_001290192, NM_001290193,NM_001290194,NM_138373	MYADM	myeloid associated differentiation marker	2.337193	1.46948E-45
6484	NM_001254757,NM_001254758,NM_001254759, NM_001348396,NM_001348397,NM_001348398, NM_001348399,NM_001348400,NM_006278,NR_145671	ST3GAL4	ST3 beta-galactoside alpha-2,3-sialyltransferase 4	2.333121	4.21025E-88
64856	NM_022834,NM_199121	VWA1	von Willebrand factor A domain containing 1	2.325877	0.005795822
2669	NM_005261,NM_181702	GEM	GTP binding protein overexpressed in skeletal muscle	2.322999	0.003322849
10397	NM_001135242,NM_001258432,NM_001258433, NM_006096	NDRG1	N-myc downstream regulated 1	2.316012	8.19325E-18
7163	NM_001025252,NM_001	TPD52	tumor protein D52	2.311504	0.017259717

	025253,NM_001287140,<br>NM_001287142,NM_001<br>287143,NM_001287144,<br>NM_005079,NR_105033,<br>NR_105034,NR_105035,<br>NR_105036,NR_105037				
164832	NM_198461	LONRF2	LON peptidase N-terminal domain and ring finger 2	2.305796	1.36108E-06
3708	NM_001099952,NM_001<br>168272,NM_002222	ITPR1	inositol 1,4,5-trisphosphate receptor type 1	2.301864	6.88832E-05
29126	NM_001267706,NM_001<br>314029,NM_014143,NR_<br>052005	CD274	CD274 molecule	2.301860	5.30667E-15
9965	NM_005117	FGF19	fibroblast growth factor 19	2.291692	1.08496E-52
1839	NM_001945	HBEGF	heparin binding EGF like growth factor	2.276884	6.87643E-07
11240	NM_007365	PADI2	peptidyl arginine deiminase 2	2.271231	1.17788E-15
112574	NM_001102575,NM_001<br>145427,NM_052870	SNX18	sorting nexin 18	2.262652	4.09351E-41
100820829	NM_001018100,NM_152<br>451	MYZAP	myocardial zonula adherens protein	2.260287	1.03033E-40
100271836	NR_027155	SMG1P3	SMG1P3, nonsense mediated mRNA decay associated PI3K related kinase pseudogene 3	2.241815	0.010224267
8351	NM_003530	HIST1H3D	histone cluster 1 H3 family member d	2.226521	0.023351291
49854	NM_001098402,NM_001<br>098403,NM_001320729,<br>NM_001320731,NM_020<br>727	ZBTB21	zinc finger and BTB domain containing 21	2.222759	0.002308992
7425	NM_003378	VGF	VGF nerve growth factor inducible	2.218771	0.001915295
5366	NM_021127	PMAIP1	phorbol-12-myristate-13-acetate-induced protein	2.207396	6.60661E-13

			1		
168455	NM_175884	CCDC71L	coiled-coil domain containing 71 like	2.200319	1.19826E-28
6781	NM_003155	STC1	stanniocalcin 1	2.191549	0.001585076
677792	NR_003026	SNORA1	small nucleolar RNA, H/ACA box 1	2.191086	0.006180666
5420	NM_001018111,NM_005397	PODXL	podocalyxin like	2.176138	0.010339434
2331	NM_002023,NR_103757	FMOD	fibromodulin	2.173312	0.026720261
4313	NM_001127891,NM_001302508,NM_001302509,NM_001302510,NM_004530	MMP2	matrix metallopeptidase 2	2.164409	0.015385435
8572	NM_001131027,NM_003687	PDLIM4	PDZ and LIM domain 4	2.161603	0.014193164
1959	NM_000399,NM_001136177,NM_001136178,NM_001136179,NM_001321037	EGR2	early growth response 2	2.147234	0.010228801
145781	NM_001018090,NM_001018091,NM_001285900,NR_104367,NR_104368,NR_104369,NR_104370,NR_104371	GCOM1	GRINL1A complex locus 1	2.146996	0.044976356
7128	NM_001270507,NM_001270508,NM_006290	TNFAIP3	TNF alpha induced protein 3	2.137841	2.92645E-06
254427	NM_153256	PROSER2	proline and serine rich 2	2.120174	9.32208E-43
10123	NM_001282431,NM_005737	ARL4C	ADP ribosylation factor like GTPase 4C	2.110321	1.1124E-30
100127888	NR_024470	SLCO4A1-AS1	SLCO4A1 antisense RNA 1	2.109623	3.20155E-09

285848	NM_001145716,NM_001145717,NM_173676	PNPLA1	patatin like phospholipase domain containing 1	2.109227	0.001987264
619567	NR_002587	SNORD2	small nucleolar RNA, C/D box 2	2.099448	0.028512101
1437	NM_000758	CSF2	colony stimulating factor 2	2.094039	0.000180958
339894	NR_034007	LINC00880	long intergenic non-protein coding RNA 880	2.090512	0.000313462
6581	NM_021977	SLC22A3	solute carrier family 22 member 3	2.090052	6.0234E-08
7151	NR_002719	TOP1P1	topoisomerase (DNA) I pseudogene 1	2.089298	0.032720728
6513	NM_006516	SLC2A1	solute carrier family 2 member 1	2.085711	1.73693E-56
360	NM_001318144,NM_004925	AQP3	aquaporin 3 (Gill blood group)	2.078793	4.95187E-29
27063	NM_014391	ANKRD1	ankyrin repeat domain 1	2.078411	0.001651042
83667	NM_031459	SESN2	sestrin 2	2.078149	6.04423E-27
5163	NM_001278549,NM_002610,NR_103729,NR_103731	PDK1	pyruvate dehydrogenase kinase 1	2.073394	0.042826941
137994	NM_001199659,NM_001199660,NM_001286787,NM_001286819,NM_001286821,NM_001330515,NM_144652	LETM2	leucine zipper and EF-hand containing transmembrane protein 2	2.065416	2.4226E-09
23657	NM_014331	SLC7A11	solute carrier family 7 member 11	2.058163	8.76275E-39
59285	NM_031897,NM_145814,NM_145815,NR_102308	CACNG6	calcium voltage-gated channel auxiliary subunit gamma 6	2.057113	2.07579E-07
9940	NM_001321153,NM_007335,NM_007337	DLEC1	deleted in lung and esophageal cancer 1	2.056522	0.003405503
4586	NM_001304359	MUC5AC	mucin 5AC, oligomeric mucus/gel-forming	2.054319	1.20413E-06
7477	NM_058238	WNT7B	Wnt family member 7B	2.046873	1.10086E-32

22822	NM_007350	PHLDA1	pleckstrin homology like domain family A member 1	2.042188	4.72694E-62
80153	NM_001142443,NM_001142444,NM_001351378,NM_001351379,NM_025083	EDC3	enhancer of mRNA decapping 3	2.030632	5.26669E-51
63979	NM_001042762,NM_001287492,NM_001287493,NM_001287494,NM_001287495,NM_001287496,NM_001346558,NM_001346559,NM_001346560,NM_001346561,NM_001346562,NM_001346563,NM_001346564,NM_001346565,NM_022116	FIGNL1	fidgetin like 1	2.021638	0.003932982
2633	NM_002053	GBP1	guanylate binding protein 1	2.021594	0.001490207
9590	NM_005100,NM_144497	AKAP12	A-kinase anchoring protein 12	2.021564	3.62708E-26
1045	NM_001265	CDX2	caudal type homeobox 2	2.021214	2.72068E-05
6274	NM_002960	S100A3	S100 calcium binding protein A3	2.017240	0.000168012
29950	NM_013376	SERTAD1	SERTA domain containing 1	2.011789	2.69252E-33
57491	NM_001242412,NM_020731	AHRR	aryl-hydrocarbon receptor repressor	2.011649	8.71824E-09
8140	NM_003486	SLC7A5	solute carrier family 7 member 5	2.000856	5.82735E-53
641649	NM_001042595,NM_001098821,NM_001098822,NM_001098823,NM_001098824,NM_001098825	TMEM91	transmembrane protein 91	-2.000339	0.001119804
283487	NR_027701	LINC00346	long intergenic non-protein coding RNA 346	-2.000835	1.51003E-14

2738	NM_138465	GLI4	GLI family zinc finger 4	-2.004452	2.34992E-05
100506810	NR_038856	LINC01132	long intergenic non-protein coding RNA 1132	-2.006112	0.007105163
728658	NR_026712	RPL13AP5	ribosomal protein L13a pseudogene 5	-2.010750	0.002730684
23177	NM_001319100,NM_001319101,NM_015147,NR_134966	CEP68	centrosomal protein 68	-2.011913	8.38263E-09
55809	NM_001297573,NM_033502	TRERF1	transcriptional regulating factor 1	-2.012895	1.63599E-18
283	NM_001097577,NM_001145	ANG	angiogenin	-2.015502	0.022409084
375287	NM_198557	RBM43	RNA binding motif protein 43	-2.021572	0.000161918
147912	NM_175875	SIX5	SIX homeobox 5	-2.021773	1.06162E-10
619279	NM_001033723	ZNF704	zinc finger protein 704	-2.023544	1.16234E-11
133746	NM_152405	JMY	junction mediating and regulatory protein, p53 cofactor	-2.025527	1.07079E-14
400986	NM_001310154	ANKRD36C	ankyrin repeat domain 36C	-2.025909	1.33964E-06
4212	NM_001220482,NM_002399,NM_170674,NM_170675,NM_170676,NM_170677,NM_172315,NM_172316,NR_051953	MEIS2	Meis homeobox 2	-2.034676	5.56109E-21
1942	NM_004428,NM_182685	EFNA1	ephrin A1	-2.036785	6.37623E-11
9514	NM_001318103,NM_001318104,NM_001318105,NM_001318106,NM_001318107,NM_001318108,NM_001318109,NM_001318110,NM_001318111,NM_001318112,NM_001	GAL3ST1	galactose-3-O-sulfotransferase 1	-2.036832	0.001169572

	318113,NM_001318114,<br>NM_001318115,NM_001<br>318116,NM_004861				
3707	NM_002221	ITPKB	inositol-trisphosphate 3-kinase B	-2.039767	1.07076E-14
100287896	NM_001319240	LOC100287896	uncharacterized LOC100287896	-2.041738	7.79124E-09
27086	NM_001012505,NM_001<br>244808,NM_001244810,<br>NM_001244812,NM_001<br>244813,NM_001244814,<br>NM_001244815,NM_001<br>244816,NM_001349337,<br>NM_001349338,NM_001<br>349339,NM_001349340,<br>NM_001349341,NM_001<br>349342,NM_001349343,<br>NM_001349344,NM_032<br>682,NR_146142,NR_146<br>143	FOXP1	forkhead box P1	-2.042796	4.42326E-19
23002	NM_001270520,NM_014<br>992	DAAM1	dishevelled associated activator of morphogenesis 1	-2.045423	1.40515E-18
285313	NM_001178145,NM_001<br>178146,NM_178822	IGSF10	immunoglobulin superfamily member 10	-2.046808	0.003918783
100271722	NR_027036	LINC00899	long intergenic non-protein coding RNA 899	-2.050820	0.003161769
57568	NM_020808	SIPA1L2	signal induced proliferation associated 1 like 2	-2.051439	1.37313E-10
3201	NM_002141	HOXA4	homeobox A4	-2.060516	6.04085E-08
84952	NM_001252335,NM_032<br>866	CGNL1	cingulin like 1	-2.067470	9.25385E-06
4661	NM_004535	MYT1	myelin transcription factor 1	-2.069241	7.43354E-06
57476	NM_001286563,NM_001<br>286564,NM_001330396,	GRAMD1B	GRAM domain containing 1B	-2.072899	3.05155E-33

	NM_020716				
6745	NM_001292008,NM_003144,NR_120448	SSR1	signal sequence receptor subunit 1	-2.073854	0.020042573
143666	NR_026967	LOC143666	uncharacterized LOC143666	-2.084615	2.47917E-05
100130581	NR_027412,NR_027413	LINC00910	long intergenic non-protein coding RNA 910	-2.091788	2.96929E-05
27244	NM_001199933,NM_001199934,NM_014454	SESN1	sestrin 1	-2.092593	1.63056E-19
65986	NM_001105539,NM_001277145,NM_023929	ZBTB10	zinc finger and BTB domain containing 10	-2.092991	0.000120953
149076	NM_152493	ZNF362	zinc finger protein 362	-2.095283	0.012822191
140766	NM_080722,NM_139155	ADAMTS14	ADAM metallopeptidase with thrombospondin type 1 motif 14	-2.096145	0.042210059
126375	NM_175872	ZNF792	zinc finger protein 792	-2.100750	7.55419E-13
102725126	NR_136306	LOC102725126	FSHD region gene 1 pseudogene	-2.109121	0.039562565
400604	NR_038458	TOB1-AS1	TOB1 antisense RNA 1	-2.110490	0.019745398
6662	NM_000346	SOX9	SRY-box 9	-2.114768	2.91489E-35
10902	NM_001164326,NM_001300961,NM_001300962,NM_001300966,NM_006696,NM_139199	BRD8	bromodomain containing 8	-2.115750	3.92904E-47
55281	NM_018295	TMEM140	transmembrane protein 140	-2.118455	0.000141496
9501	NM_001190411,NM_001190412,NM_001190413,NM_006987	RPH3AL	rabphilin 3A like (without C2 domains)	-2.120876	0.028309383
55701	NM_001278529,NM_001278530,NM_018071	ARHGEF40	Rho guanine nucleotide exchange factor 40	-2.125223	7.67973E-10
138199	NM_001320497,NM_152	CARNMT1	carnosine N-methyltransferase 1	-2.126288	0.014904857

	420,NR_135282				
64599	NM_022574	GIGYF1	GRB10 interacting GYF protein 1	-2.128060	2.40231E-49
402415	NM_212559	XKRX	XK related, X-linked	-2.128247	1.70275E-06
1944	NM_004952	EFNA3	ephrin A3	-2.135306	0.000281691
8654	NM_001083,NM_033430,NM_033437	PDE5A	phosphodiesterase 5A	-2.135979	0.000130245
26140	NM_001025930,NR_037162	TTLL3	tubulin tyrosine ligase like 3	-2.136430	0.000317746
3221	NM_014620,NM_153633	HOXC4	homeobox C4	-2.137568	1.08367E-05
283991	NM_182565	UBALD2	UBA like domain containing 2	-2.141023	1.93077E-29
135932	NM_001242773,NM_001242774,NM_001242775,NM_001282876,NM_001282877,NM_153345,NR_040003,NR_104250,NR_104251,NR_104252,NR_104253,NR_104254	TMEM139	transmembrane protein 139	-2.144604	6.33772E-42
23248	NM_001297673,NM_001297674,NM_015203	RPRD2	regulation of nuclear pre-mRNA domain containing 2	-2.145428	0.021964959
5444	NM_000446	PON1	paraoxonase 1	-2.146405	0.027206597
101928079	NR_104131	SLC44A3-AS1	SLC44A3 antisense RNA 1	-2.147474	0.045641487
4215	NM_001330431,NM_002401,NM_203351	MAP3K3	mitogen-activated protein kinase kinase kinase 3	-2.149370	3.62239E-28
11074	NM_007028,NR_134870,NR_134871	TRIM31	tripartite motif containing 31	-2.149389	8.83604E-56
118738	NM_001346932,NM_001346933,NM_001346934,NM_001346935,NM_001	ZNF488	zinc finger protein 488	-2.152253	9.47493E-18

	346936,NM_153034				
79960	NM_001287437,NM_001287439,NM_001287440,NM_001287441,NM_001287442,NM_001287443,NM_024900,NM_199320	JADE1	jade family PHD finger 1	-2.156453	7.91739E-20
286336	NM_033387	FAM78A	family with sequence similarity 78 member A	-2.156977	0.005752239
256691	NM_001347990,NM_153267,NR_125850	MAMDC2	MAM domain containing 2	-2.157780	0.006761224
105374105	NR_146710	LINC02014	long intergenic non-protein coding RNA 2014	-2.159366	0.041177908
8431	NM_021969	NR0B2	nuclear receptor subfamily 0 group B member 2	-2.160623	0.001986097
143879	NM_001330359,NM_152433,NM_198439	KBTBD3	kelch repeat and BTB domain containing 3	-2.160680	1.73812E-08
126272	NM_152361	EID2B	EP300 interacting inhibitor of differentiation 2B	-2.164072	0.000138876
283460	NR_024345	HNF1A-AS1	HNF1A antisense RNA 1	-2.170476	6.39426E-05
79818	NM_024762	ZNF552	zinc finger protein 552	-2.172415	3.01563E-34
54567	NM_019074	DLL4	delta like canonical Notch ligand 4	-2.181370	0.008477062
6666	NM_006943	SOX12	SRY-box 12	-2.185934	1.51729E-10
25928	NM_015464	SOSTDC1	sclerostin domain containing 1	-2.192398	1.24235E-20
115761	NM_138450	ARL11	ADP ribosylation factor like GTPase 11	-2.194899	0.029047981
4322	NM_002427	MMP13	matrix metallopeptidase 13	-2.201102	0.005427818
1028	NM_000076,NM_001122630,NM_001122631	CDKN1C	cyclin dependent kinase inhibitor 1C	-2.217160	3.65608E-06
55108	NM_001143888,NM_001143889,NM_001143890,NM_001300958,NM_018045,NR_125352	BSDC1	BSD domain containing 1	-2.217842	0.008689981

80790	NM_030629,NM_198390	CMIP	c-Maf inducing protein	-2.219218	0.026448742
11119	NM_001145008,NM_001145009,NM_007048,NM_194441	BTN3A1	butyrophilin subfamily 3 member A1	-2.219566	3.75757E-06
84787	NM_032701	KMT5C	lysine methyltransferase 5C	-2.220369	9.42585E-14
9771	NM_012294	RAPGEF5	Rap guanine nucleotide exchange factor 5	-2.223952	4.81324E-10
54899	NM_001289095,NM_001289096,NM_001289098,NM_001289099,NM_001289100,NM_001289101,NM_001349488,NM_001349489,NM_001349490,NM_001349491,NM_001349492,NM_001349493,NM_001349494,NM_001349495,NM_001349496,NM_001349497,NM_001349498,NM_001349499,NM_001349500,NM_001349501,NM_001349502,NM_001349503,NM_001349504,NM_001349506,NM_001349507,NM_001349508,NM_001349509,NM_001349510,NM_001349511,NM_001349512,NM_001349513,NM_001349514,NM_001349515,NM_001349516,NM_001349517,NM_001349518,NM_001349519,NM_001349520,NM_001349521,NM_001349522,NM_001	PXK	PX domain containing serine/threonine kinase like	-2.224303	0.004090299

	349524,NM_001349525, NM_001349526,NM_001349527,NM_001349528, NM_001349529,NM_001349530,NM_001349531, NM_001349532,NM_001349533,NM_001349534, NM_001349535,NM_001349536,NM_001349537, NM_001349538,NM_001349539,NM_001349540, NM_017771,NR_146193, NR_146194				
51265	NM_001113575,NM_001300853,NM_001349363, NM_001349364,NM_001349365,NM_001349366, NM_016508	CDKL3	cyclin dependent kinase like 3	-2.225359	0.003990332
100507564	NR_038953	LOC100507564	uncharacterized LOC100507564	-2.231478	0.034865193
374907	NM_198540	B3GNT8	UDP-GlcNAc:betaGal beta-1,3-N-acetylglucosaminyltransferase 8	-2.235287	0.005485022
401427	NM_001005328	OR2A7	olfactory receptor family 2 subfamily A member 7	-2.243263	0.028009991
2624	NM_001145661,NM_001145662,NM_032638	GATA2	GATA binding protein 2	-2.247863	1.01379E-58
391322	NM_001144931	LOC391322	D-dopachrome tautomerase-like	-2.259800	0.033805828
79156	NM_024310	PLEKHF1	pleckstrin homology and FYVE domain containing 1	-2.275888	0.001262455
2302	NM_001454	FOXJ1	forkhead box J1	-2.278521	0.001205449
91461	NM_138370	PKDCC	protein kinase domain containing, cytoplasmic	-2.281410	6.13778E-27

100124700	NR_003716,NR_047517,NR_047518	HOTAIR	HOX transcript antisense RNA	-2.282267	9.66504E-18
400798	NR_033186	C1orf220	chromosome 1 open reading frame 220	-2.284086	0.044244403
55521	NM_001017397,NM_001017398,NM_001300752,NM_001300759,NM_018700	TRIM36	tripartite motif containing 36	-2.285408	0.023430038
3223	NM_004503,NM_153693	HOXC6	homeobox C6	-2.285542	6.48658E-10
9481	NM_001204051,NM_001204052,NM_004277	SLC25A27	solute carrier family 25 member 27	-2.285879	2.75173E-09
3660	NM_002199	IRF2	interferon regulatory factor 2	-2.289977	1.22045E-17
125170	NM_001144900,NM_139162,NM_148886	MIEF2	mitochondrial elongation factor 2	-2.291195	1.68502E-17
283876	NR_033904	LINC00921	long intergenic non-protein coding RNA 921	-2.297351	0.018934569
93	NM_001106	ACVR2B	activin A receptor type 2B	-2.299519	1.28607E-12
124751	NM_001304947,NM_213597	KRBA2	KRAB-A domain containing 2	-2.312777	0.016722397
55315	NM_001174098,NM_018344,NR_033413,NR_033414	SLC29A3	solute carrier family 29 member 3	-2.315817	1.26613E-18
7294	NM_003328	TXK	TXK tyrosine kinase	-2.317667	0.00172095
84666	NM_032579	RETNLB	resistin like beta	-2.326930	0.045574408
53349	NM_001281734,NM_001281735,NM_021260,NM_178441	ZFYVE1	zinc finger FYVE-type containing 1	-2.338932	1.4293E-08
23401	NM_012083	FRAT2	FRAT2, WNT signaling pathway regulator	-2.340012	2.37105E-32
5453	NM_002699	POU3F1	POU class 3 homeobox 1	-2.342706	0.000444353

140738	NM_183240	TMEM37	transmembrane protein 37	-2.343463	1.8251E-05
121268	NM_001303126,NM_144593,NR_130123	RHEBL1	Ras homolog enriched in brain like 1	-2.345184	0.000205079
80352	NM_025236,NM_170769	RNF39	ring finger protein 39	-2.347094	1.55773E-10
10420	NM_001320800,NM_007170	TESK2	testis-specific kinase 2	-2.349675	8.10348E-09
148418	NM_001010971,NM_001134663,NM_001134664	SAMD13	sterile alpha motif domain containing 13	-2.350638	0.003101361
11067	NM_007021	C10orf10	chromosome 10 open reading frame 10	-2.361606	2.39364E-07
80853	NM_030647	KDM7A	lysine demethylase 7A	-2.363573	4.90318E-10
4781	NM_001190737,NM_001190738,NM_001282787,NM_005596	NFIB	nuclear factor I B	-2.375905	1.7552E-28
3706	NM_002220	ITPKA	inositol-trisphosphate 3-kinase A	-2.384256	0.006760443
147011	NM_001304949,NM_001304951,NM_001304952,NM_001304953,NM_001304954,NM_152465	PROCA1	protein interacting with cyclin A1	-2.385062	0.006041175
145788	NM_001198784	C15orf65	chromosome 15 open reading frame 65	-2.397576	1.42333E-06
3268	NM_006076	AGFG2	ArfGAP with FG repeats 2	-2.412339	4.30882E-32
100130172	NR_037900	LOC100130172	uncharacterized LOC100130172	-2.415106	0.024665799
8408	NM_003565	ULK1	unc-51 like autophagy activating kinase 1	-2.421657	1.35572E-22
79849	NM_001168468,NM_024791,NR_033122	PDZD3	PDZ domain containing 3	-2.430099	0.000878735
55503	NM_018646	TRPV6	transient receptor potential cation channel subfamily V member 6	-2.434226	0.028217301
10402	NM_001271142,NM_001	ST3GAL6	ST3 beta-galactoside alpha-2,3-sialyltransferase	-2.436052	0.034746191

	271145,NM_001271146,NM_001271147,NM_001271148,NM_001323352,NM_001323353,NM_001323358,NM_001323359,NM_001323360,NM_001323362,NM_001323363,NM_001323364,NM_001323365,NM_001323366,NM_001323367,NM_001323368,NM_006100		6		
25822	NM_001135004,NM_001135005,NM_001349723,NM_001349724,NM_001349725,NM_012266	DNAJB5	DnaJ heat shock protein family (Hsp40) member B5	-2.436501	0.008953561
389384	NM_001010903	C6orf222	chromosome 6 open reading frame 222	-2.436702	0.011930825
8646	NM_001304472,NM_001304473,NM_001304474,NM_003741,NR_130747	CHRD	chordin	-2.448884	0.032337721
2263	NM_000141,NM_001144913,NM_001144914,NM_001144915,NM_001144916,NM_001144917,NM_001144918,NM_001144919,NM_001320654,NM_001320658,NM_022970,NM_023029,NR_073009	FGFR2	fibroblast growth factor receptor 2	-2.470570	5.91603E-38
146330	NM_153350	FBXL16	F-box and leucine rich repeat protein 16	-2.470816	0.024139757
153020	NM_001300735,NM_001300736,NM_152545	RASGEF1B	RasGEF domain family member 1B	-2.471993	0.011721877
3760	NM_001260508,NM_001	KCNJ3	potassium voltage-gated channel subfamily J	-2.478320	0.00010366

	260509,NM_001260510, NM_002239		member 3		
140893	NM_080833	RBBP8NL	RBBP8 N-terminal like	-2.480655	1.4903E-13
54943	NM_001040192,NM_001320746,NM_017833	DNAJC28	DnaJ heat shock protein family (Hsp40) member C28	-2.485050	0.003509181
84206	NM_032246	MEX3B	mex-3 RNA binding family member B	-2.489985	0.00011926
51063	NM_015916,NR_024552, NR_046344	CALHM2	calcium homeostasis modulator 2	-2.507757	4.94941E-10
171523	NR_144551	CYP2T1P	cytochrome P450 family 2 subfamily T member 1, pseudogene	-2.508933	0.011223731
643224	NR_027156	TUBBP5	tubulin beta pseudogene 5	-2.515162	0.000756579
9881	NM_001329998,NM_014831	TRANK1	tetratricopeptide repeat and ankyrin repeat containing 1	-2.515379	7.22618E-09
56670	NM_033050	SUCNR1	succinate receptor 1	-2.526491	0.013616041
9754	NM_001142503,NM_001142504,NM_014725	STARD8	StAR related lipid transfer domain containing 8	-2.529701	0.007631885
4857	NM_002515,NM_006489,NM_006491	NOVA1	NOVA alternative splicing regulator 1	-2.536522	0.002628715
284390	NM_001012753	ZNF763	zinc finger protein 763	-2.541747	0.002336205
150759	NR_103734	LINC00342	long intergenic non-protein coding RNA 342	-2.541918	2.40694E-12
50640	NM_001256007,NM_001256008,NM_001256009, NM_001256010,NM_001256011,NM_015723	PNPLA8	patatin like phospholipase domain containing 8	-2.549353	2.39178E-06
692247	NR_147607,NR_147608	LOC692247	uncharacterized LOC692247	-2.563328	0.034056902
440925	NR_027433	LINC01124	long intergenic non-protein coding RNA 1124	-2.567584	0.014481564
1041	NM_001264	CDSN	corneodesmosin	-2.571259	0.023049373

153222	NM_001168393,NM_001168394,NM_153607	CREBRF	CREB3 regulatory factor	-2.592848	1.12361E-16
10008	NM_005472	KCNE3	potassium voltage-gated channel subfamily E regulatory subunit 3	-2.613029	1.34468E-46
5801	NM_001207015,NM_001207016,NM_002849,NM_130846,NR_073474	PTPRR	protein tyrosine phosphatase, receptor type R	-2.619971	1.73308E-06
65249	NM_023072	ZSWIM4	zinc finger SWIM-type containing 4	-2.623646	3.73855E-11
100506211	NR_038262	MIR210HG	MIR210 host gene	-2.624467	0.001465882
151050	NM_001307976,NM_152519	KANSL1L	KAT8 regulatory NSL complex subunit 1 like	-2.635078	1.22658E-18
114991	NM_001318040,NM_001318041,NM_001318042, NM_133374	ZNF618	zinc finger protein 618	-2.637277	0.007742573
5166	NM_002612	PDK4	pyruvate dehydrogenase kinase 4	-2.643950	1.26808E-07
5727	NM_000264,NM_001083602,NM_001083603,NM_001083604,NM_001083605,NM_001083606,NM_001083607	PTCH1	patched 1	-2.644359	7.62094E-24
7049	NM_001195683,NM_001195684,NM_003243,NR_036634	TGFBR3	transforming growth factor beta receptor 3	-2.680618	3.05842E-20
84708	NM_001126328,NM_032622	LNX1	ligand of numb-protein X 1	-2.681327	3.67293E-13
203102	NM_001313994,NM_145004	ADAM32	ADAM metallopeptidase domain 32	-2.695968	0.045315912
54823	NM_001105518,NM_017673	SWT1	SWT1, RNA endoribonuclease homolog	-2.697513	6.99381E-11

255488	NM_182757	RNF144B	ring finger protein 144B	-2.701887	4.6069E-13
388403	NM_001005404	YPEL2	yippee like 2	-2.702401	3.83035E-14
154043	NM_173515	CNKSR3	CNKSR family member 3	-2.706868	0.014655299
570	NM_001127610,NM_001701	BAAT	bile acid-CoA:amino acid N-acyltransferase	-2.714458	0.006142394
80054	NR_026887	CEBPA-AS1	CEBPA antisense RNA 1 (head to head)	-2.746127	0.002053536
84225	NM_001136046,NM_001267822,NM_032265	ZMYND15	zinc finger MYND-type containing 15	-2.750226	0.028834904
283209	NM_173582	PGM2L1	phosphoglucomutase 2 like 1	-2.775190	3.66781E-20
100129722	NR_038389	STPG3-AS1	STPG3 antisense RNA 1	-2.783711	0.044087949
19	NM_005502	ABCA1	ATP binding cassette subfamily A member 1	-2.797493	1.07021E-09
9469	NM_004273	CHST3	carbohydrate sulfotransferase 3	-2.816502	7.1416E-20
8000	NM_005672,NR_033343	PSCA	prostate stem cell antigen	-2.823519	3.68944E-16
57730	NM_025190	ANKRD36B	ankyrin repeat domain 36B	-2.827125	8.94813E-09
152926	NM_152542	PPM1K	protein phosphatase, Mg2+/Mn2+ dependent 1K	-2.836499	2.04188E-12
400673	NM_001017921	VMAC	vimentin-type intermediate filament associated coiled-coil protein	-2.850594	7.77788E-07
56204	NM_001286495,NM_019600,NR_104457	FAM214A	family with sequence similarity 214 member A	-2.860436	1.12003E-12
728661	NM_001110781,NM_001290264	SLC35E2B	solute carrier family 35 member E2B	-2.867416	0.044463585
56964	NM_001284395,NM_001284396,NM_020212	WDR93	WD repeat domain 93	-2.869681	0.037589887
80125	NM_001287181,NM_025055,NM_182791	CCDC33	coiled-coil domain containing 33	-2.884855	0.042331391
81788	NM_030952	NUAK2	NUAK family kinase 2	-2.930433	2.20151E-10

79170	NM_024320	PRR15L	proline rich 15 like	-2.931377	2.76266E-28
868	NM_001321786,NM_001321788,NM_001321789, NM_001321790,NM_001321791,NM_001321793, NM_001321794,NM_001321795,NM_001321796, NM_001321797,NM_001321798,NM_001321799, NM_001321806,NM_001321807,NM_001321808, NM_001321811,NM_001321813,NM_001321816, NM_001321820,NM_001321822,NM_170662,NR_135806,NR_135807,NR_135808,NR_135809,NR_135810,NR_135811,NR_135812	CBLB	Cbl proto-oncogene B	-2.933387	4.52894E-26
8743	NM_001190942,NM_001190943,NM_003810,NR_033994	TNFSF10	TNF superfamily member 10	-2.937008	2.93864E-20
2306	NM_004474	FOXD2	forkhead box D2	-2.939699	0.00014124
4306	NM_000901,NM_001166104	NR3C2	nuclear receptor subfamily 3 group C member 2	-2.993710	0.00644183
10957	NM_006813	PNRC1	proline rich nuclear receptor coactivator 1	-2.996192	1.53335E-16
100996741	NM_001348152	LOC100996741	uncharacterized LOC100996741	-3.018987	0.025225247
353355	NM_001207005,NM_001330529,NM_181756	ZNF233	zinc finger protein 233	-3.026900	4.33786E-05
604	NM_001130845,NM_001134738,NM_001706	BCL6	B-cell CLL/lymphoma 6	-3.061415	3.21364E-35

389072	NM_001080475	PLEKHM3	pleckstrin homology domain containing M3	-3.064015	4.9333E-25
728039	NR_027292	SSR4P1	signal sequence receptor subunit 4 pseudogene 1	-3.086888	0.029732538
284161	NM_001165993,NM_001165994,NM_182569	GDPD1	glycerophosphodiester phosphodiesterase domain containing 1	-3.096141	0.008566433
389058	NM_001003845	SP5	Sp5 transcription factor	-3.113411	1.58137E-08
1906	NM_001168319,NM_001955	EDN1	endothelin 1	-3.117498	7.1134E-55
85376	NM_015672	RIMBP3	RIMS binding protein 3	-3.142990	0.036404038
6372	NM_002993	CXCL6	C-X-C motif chemokine ligand 6	-3.155778	0.005594648
5013	NM_001199770,NM_014562,NR_130153	OTX1	orthodenticle homeobox 1	-3.254754	3.33608E-21
7832	NM_006763	BTG2	BTG anti-proliferation factor 2	-3.285938	7.07287E-46
100134868	NR_004846	LOC100134868	uncharacterized LOC100134868	-3.298808	0.026541877
100506084	NM_001039083,NM_001103154,NM_001330240,NM_001330240_dup1,NM_001330240_dup2	ARL17B	ADP ribosylation factor like GTPase 17B	-3.316684	0.014368957
10194	NM_001308210,NM_005786	TSHZ1	teashirt zinc finger homeobox 1	-3.329511	1.09996E-48
83719	NM_001145524,NM_031477	YPEL3	yippee like 3	-3.385649	1.98541E-23
84189	NM_032229	SLITRK6	SLIT and NTRK like family member 6	-3.393941	3.28623E-50
494513	NM_001042702	DFNB59	deafness, autosomal recessive 59	-3.406434	0.007928289
63982	NM_001313726,NM_001313727,NM_031418	ANO3	anoctamin 3	-3.433070	1.81407E-06
83875	NM_001037290,NM_001256397,NM_001256398,	BCO2	beta-carotene oxygenase 2	-3.436449	0.025476223

	NM_001256400,NM_031938				
57653	NR_036527,NR_036528,NR_036529	LOC100499484-C9ORF174	LOC100499484-C9orf174 readthrough	-3.496523	0.002753204
399821	NR_033847	FLJ37035	uncharacterized LOC399821	-3.509572	0.00294633
57507	NM_020747	ZNF608	zinc finger protein 608	-3.513394	2.23631E-17
4602	NM_001130172,NM_001130173,NM_001161656,NM_001161657,NM_001161658,NM_001161659,NM_001161660,NM_005375,NR_134958,NR_134959,NR_134960,NR_134961,NR_134962,NR_134963,NR_134964,NR_134965	MYB	MYB proto-oncogene, transcription factor	-3.571684	4.01031E-28
26959	NM_001244262,NM_012257	HBP1	HMG-box transcription factor 1	-3.595660	3.1189E-56
333929	NM_178310	SNAI3	snail family transcriptional repressor 3	-3.615010	0.001287168
5624	NM_000312	PROC	protein C, inactivator of coagulation factors Va and VIIIa	-3.615844	0.006860512
3750	NM_004979	KCND1	potassium voltage-gated channel subfamily D member 1	-3.637660	0.006085576
284723	NM_207348	SLC25A34	solute carrier family 25 member 34	-3.651650	0.033507094
114800	NM_001080433,NM_001348512,NM_001348513,NM_001348514,NM_001348515,NM_001348516	CCDC85A	coiled-coil domain containing 85A	-3.743221	0.041051922
113791	NM_001135911,NM_052880	PIK3IP1	phosphoinositide-3-kinase interacting protein 1	-3.749360	0.00056901

83992	NM_033427	CTTNBP2	cortactin binding protein 2	-3.758192	3.0954E-07
2199	NM_001004019,NM_001165035,NM_001998	FBLN2	fibulin 2	-3.882214	0.01144694
83478	NM_001025616,NM_001042669,NM_001287805,NM_001346093,NM_031305	ARHGAP24	Rho GTPase activating protein 24	-4.012685	0.002118861
53840	NM_001003827,NM_021616,NM_130390	TRIM34	tripartite motif containing 34	-4.029748	0.020175162
440804	NM_001128635,NM_001128635_dup1	RIMBP3B	RIMS binding protein 3B	-4.117855	0.026404111
261729	NM_001040665,NM_001040666,NM_001244944,NM_001244945,NM_001244946,NM_152999	STEAP2	STEAP2 metalloreductase	-4.144198	0.012923035
29799	NM_013313,NR_130910	YPEL1	yippee like 1	-4.152153	5.80717E-05
114819	NR_023386	CROCCP3	ciliary rootlet coiled-coil, rootletin pseudogene 3	-4.291523	0.000212619
106821730	NM_001128628,NM_001128629	BUB1B-PAK6	BUB1B-PAK6 readthrough	-4.400101	0.035643721
25764	NM_001199885,NM_016400	HYPK	huntingtin interacting protein K	-4.550992	1.65864E-08
54800	NM_001349413,NM_001349414,NM_001349415,NM_001349416,NM_001349417,NM_001349418,NM_001349419,NM_001349420,NM_001349421,NM_001349422,NM_001349423,NM_001349424,NM_001349425,NM_001	KLHL24	kelch like family member 24	-4.561804	3.56391E-07

	349426,NM_001349427, NM_001349428,NM_001 349429,NM_017644,NR_ 146169,NR_146170				
729540	NM_001037866,NM_001 037866_dup1,NM_00112 3363,NM_001123363_du p1	RGPD6	RANBP2-like and GRIP domain containing 6	-4.562495	0.036527558
10023	NM_005479	FRAT1	FRAT1, WNT signaling pathway regulator	-4.573867	5.05807E-10
23285	NM_015237	KIAA1107	KIAA1107	-4.606404	3.70513E-10
286204	NM_173689,NR_104603	CRB2	crumbs 2, cell polarity complex component	-4.717512	1.94476E-08
2886	NM_001030002,NM_001 242442,NM_001242443, NM_001330207,NM_005 310	GRB7	growth factor receptor bound protein 7	-5.015969	7.35429E-69
729533	NM_001123168,NM_001 317901,NR_134239	FAM72A	family with sequence similarity 72 member A	-5.067758	0.024565155
84159	NM_001244638,NM_032 199	ARID5B	AT-rich interaction domain 5B	-5.172166	1.4704E-105
26152	NM_001290261,NM_015 655	ZNF337	zinc finger protein 337	-6.200863	0.008729761
94241	NM_001135733,NM_033 285	TP53INP1	tumor protein p53 inducible nuclear protein 1	-6.397238	5.99016E-15
3020	NM_002107	H3F3A	H3 histone family member 3A	-13.262167	0.008363007
100861412	NM_001256141	FSBP	fibrinogen silencer binding protein	-13.296177	0.003590694
54809	NM_001193307,NM_017 654	SAMD9	sterile alpha motif domain containing 9	-28.867482	0.010328588
729438	NM_001145064	CASTOR2	cytosolic arginine sensor for mTORC1 subunit 2	-29.786042	1.5073E-15

Table S6 Gene ontology (GO) enrichment analysis of differentially expressed genes in HT-29 stimulated with wild-type spore

GO ID	GO description	*p*-value	Corrected *p*-value[a]	Cluster frequency[b]	Total frequency[c]	Genes
786	nucleosome	2.04E-08	1.77E-05	6/48 12.5%	65/17791 0.3%	*HIST1H2BO HIST1H2AI HIST1H2BF HIST1H2BG HIST2H3C HIST1H3D*
6334	nucleosome assembly	9.00E-08	3.08E-05	6/48 12.5%	83/17791 0.4%	*HIST1H2BO HIST1H2AI HIST1H2BF HIST1H2BG HIST2H3C HIST1H3D*
31497	chromatin assembly	1.20E-07	3.08E-05	6/48 12.5%	87/17791 0.4%	*HIST1H2BO HIST1H2AI HIST1H2BF HIST1H2BG HIST2H3C HIST1H3D*
65004	protein-DNA complex assembly	1.67E-07	3.08E-05	6/48 12.5%	92/17791 0.5%	*HIST1H2BO HIST1H2AI HIST1H2BF HIST1H2BG HIST2H3C HIST1H3D*
34728	nucleosome organization	1.78E-07	3.08E-05	6/48 12.5%	93/17791 0.5%	*HIST1H2BO HIST1H2AI HIST1H2BF HIST1H2BG HIST2H3C HIST1H3D*
32993	protein-DNA complex	2.29E-07	3.30E-05	6/48 12.5%	97/17791 0.5%	*HIST1H2BO HIST1H2AI HIST1H2BF HIST1H2BG HIST2H3C HIST1H3D*
6323	DNA packaging	7.32E-07	9.04E-05	6/48 12.5%	118/17791 0.6%	*HIST1H2BO HIST1H2AI HIST1H2BF HIST1H2BG HIST2H3C HIST1H3D*
6333	chromatin assembly or disassembly	1.13E-06	1.22E-04	6/48 12.5%	127/17791 0.7%	*HIST1H2BO HIST1H2AI HIST1H2BF HIST1H2BG HIST2H3C HIST1H3D*
71103	DNA conformation change	1.91E-06	1.84E-04	6/48 12.5%	139/17791 0.7%	*HIST1H2BO HIST1H2AI HIST1H2BF HIST1H2BG HIST2H3C HIST1H3D*
785	chromatin	1.64E-05	1.42E-03	6/48 12.5%	202/17791 1.1%	*HIST1H2BO HIST1H2AI HIST1H2BF HIST1H2BG HIST2H3C HIST1H3D*

34622	cellular macromolecular complex assembly	2.06E-05	1.62E-03	7/48 14.5%	316/17791 1.7%	*TUBA1A HIST1H2BO HIST1H2AI HIST1H2BF HIST1H2BG HIST2H3C HIST1H3D*
34621	cellular macromolecular complex subunit organization	4.40E-05	3.17E-03	7/48 14.5%	356/17791 2.0%	*TUBA1A HIST1H2BO HIST1H2AI HIST1H2BF HIST1H2BG HIST2H3C HIST1H3D*
65003	macromolecular complex assembly	6.95E-05	4.62E-03	9/48 18.7%	676/17791 3.7%	*TUBA1A HIST1H2BO HIST1H2AI HIST1H2BF HIST1H2BG HIST2H3C PPARGC1A HIST1H3D TGM2*
43933	macromolecular complex subunit organization	1.17E-04	7.24E-03	9/48 18.7%	724/17791 4.0%	*TUBA1A HIST1H2BO HIST1H2AI HIST1H2BF HIST1H2BG HIST2H3C PPARGC1A HIST1H3D TGM2*
44427	chromosomal part	6.10E-04	3.51E-02	6/48 12.5%	391/17791 2.1%	*HIST1H2BO HIST1H2AI HIST1H2BF HIST1H2BG HIST2H3C HIST1H3D*
22607	cellular component assembly	6.53E-04	3.53E-02	9/48 18.7%	913/17791 5.1%	*TUBA1A HIST1H2BO HIST1H2AI HIST1H2BF HIST1H2BG HIST2H3C PPARGC1A HIST1H3D TGM2*
6325	chromatin organization	8.12E-04	4.13E-02	6/48 12.5%	413/17791 2.3%	*HIST1H2BO HIST1H2AI HIST1H2BF HIST1H2BG HIST2H3C HIST1H3D*

[a] *p*-value in hypergeometric test after correction

[b] The denominator represents the total number of genes with GO annotation and the numerator represents the number of genes belonging to each GO term

[c] The denominator represents the number of reference genes with GO annotation and the numerator represents the number of references genes annotated in the listed GO terms

Table S7 Gene ontology (GO) enrichment analysis of differentially expressed genes in HT-29 stimulated with CotG-p75

GO ID	GO description	*p*-value	Corrected *p*-value[a]	Cluster frequency[b]	Total frequency[c]	Genes
48731	system development	5.92E-12	2.04E-08	103/387 26.6%	2421/17777 13.6%	*ERRFI1 CSF2 SERPINE1 TNC IRS2 AQP3 GJA5 MYC SALL4 DPF1 HOXA5 KIRREL3 EPHA4 UNC5B OSR1 ARID5B POU3F1 MYT1 GEM EREG TXNIP ULK1 TRIM15 TAGLN3 FREM2 FLG SEMA7A PKDCC TSHZ1 STC1 GATA2 OTX1 HOXC6 EGR1 FZD2 FZD4 PTCH1 LRRN4 NR0B2 ARHGAP24 POU6F1 EFNA1 NR4A2 IGSF10 GJB2 TMIE BCL6 FGF19 CYP1A1 SPRR1A SPRR1B CDKN1C GPSM1 CSRNP1 CDKN1A BTG2 PTPRR GAL3ST1 CXCL8 CITED2 CHD6 SECTM1 FASLG PTPRJ SOBP GLI2 LFNG SIX5 ZNF488 EID2B SNTA1 EDN1 ITPKB TGFBR3 SLC7A5 ALDH1A3 AHRR PDE5A RAPGEF5 IVL MDFI KDM7A HBEGF CHST3 SPRR3 SRF PTGS2 DLL4 ERBB3 SLITRK6 SH2B3 ATOH1 CDX2 WNT7B GRHL3 CCK ACVR2B APLN GATM EPGN DHRS9 KRT17 FMOD*
48856	anatomical structure development	1.24E-10	2.14E-07	106/387 27.3%	2655/17777 14.9%	*ERRFI1 CSF2 SERPINE1 TNC IRS2 AQP3 GJA5 MYC SALL4 DPF1 HOXA5 KIRREL3 EPHA4 UNC5B OSR1 ARID5B BHLHA15 POU3F1 MYT1 GEM EREG TXNIP ULK1 TRIM15 TAGLN3 FREM2 FLG SEMA7A PKDCC TSHZ1 STC1 GATA2 OTX1 HOXC6 EGR1 FZD2 FZD4 PTCH1 LRRN4 NR0B2 ARHGAP24 POU6F1 EFNA1 NR4A2 IGSF10 GJB2 TMIE BCL6 FGF19 CYP1A1 SPRR1A SPRR1B CDKN1C GPSM1 CSRNP1 CDKN1A BTG2 PTPRR GAL3ST1 CXCL8 CITED2 SLC40A1 CHD6 SECTM1 FASLG PTPRJ SOBP GLI2 LFNG SIX5 ZNF488 EID2B SNTA1 EDN1 PTBP3 ITPKB TGFBR3 SLC7A5 ALDH1A3 AHRR PDE5A RAPGEF5 IVL MDFI KDM7A HBEGF CHST3 SPRR3 SRF PTGS2 DLL4 ERBB3 SLITRK6 SH2B3 ATOH1 CDX2 WNT7B GRHL3 CCK ACVR2B APLN GATM EPGN DHRS9 KRT17 FMOD*

48513	organ development	2.82E-10	3.23E-07	80/387 20.6%	1791/17777 10.0%	*ERRFI1 CSRNP1 CDKN1A CSF2 CXCL8 CITED2 SERPINE1 TNC SECTM1 IRS2 FASLG PTPRJ SOBP AQP3 GLI2 LFNG SIX5 GJA5 MYC SALL4 HOXA5 EID2B SNTA1 KIRREL3 EDN1 OSR1 ARID5B POU3F1 GEM EREG ITPKB TGFBR3 ALDH1A3 TXNIP AHRR ULK1 TRIM15 TAGLN3 FREM2 IVL MDFI KDM7A HBEGF FLG SPRR3 PKDCC SRF TSHZ1 GATA2 PTGS2 DLL4 ERBB3 OTX1 SH2B3 ATOH1 EGR1 FZD2 CDX2 WNT7B PTCH1 GRHL3 NR0B2 ACVR2B ARHGAP24 POU6F1 APLN EFNA1 IGSF10 GJB2 GATM TMIE EPGN DHRS9 BCL6 KRT17 FGF19 CYP1A1 FMOD SPRR1A SPRR1B*
9653	anatomical structure morphogenesis	1.30E-08	1.12E-05	58/387 14.9%	1217/17777 6.8%	*ERRFI1 CSRNP1 CDKN1A CXCL8 CITED2 SERPINE1 TNC SLC40A1 FASLG PTPRJ SOBP AQP3 GLI2 LFNG GJA5 MYC SALL4 HOXA5 EPHA4 EDN1 UNC5B OSR1 ARID5B GEM PTBP3 EREG TGFBR3 ALDH1A3 ULK1 TRIM15 PDE5A FREM2 MDFI CHST3 FLG SRF TSHZ1 DLL4 ERBB3 SLITRK6 OTX1 ATOH1 FZD2 CDX2 WNT7B PTCH1 CCK NR0B2 ACVR2B ARHGAP24 EFNA1 NR4A2 IGSF10 GATM TMIE EPGN BCL6 FMOD*
7275	multicellular organismal development	1.68E-08	1.16E-05	108/387 27.9%	2970/17777 16.7%	*ERRFI1 CSF2 SERPINE1 TNC IRS2 AQP3 FRAT1 FRAT2 GJA5 MYC SALL4 DPF1 HOXA5 KIRREL3 EPHA4 UNC5B OSR1 ARID5B POU3F1 MYT1 GEM EREG MFNG TXNIP ULK1 TRIM15 TAGLN3 FREM2 FLG SEMA7A PKDCC TSHZ1 STC1 GATA2 OTX1 HOXC6 EGR1 FZD2 FZD4 PTCH1 LRRN4 NR0B2 ARHGAP24 POU6F1 EFNA1 NR4A2 IGSF10 GJB2 TMIE BCL6 FGF19 CYP1A1 SPRR1A SPRR1B CDKN1C GPSM1 CSRNP1 CDKN1A BTG2 PTPRR GAL3ST1 CXCL8 CITED2 CHD6 SECTM1 FASLG PTPRJ SOBP GLI2 LFNG SPRED3 SIX5 ZNF488 EID2B SNTA1 EDN1 ITPKB TGFBR3 SLC7A5 ALDH1A3 AHRR PDE5A RAPGEF5 IVL MDFI KDM7A HBEGF CHST3 SPRR3 CATSPER2 SRF PTGS2 DLL4 ERBB3 SLITRK6 SH2B3 ATOH1 CDX2 WNT7B GRHL3 CCK ACVR2B APLN GATM EPGN DHRS9 KRT17 FMOD*

32502	developmental process	6.15E-08	3.53E-05	113/387 29.1%	3233/17777 18.1%	*ERRFI1 CSF2 SERPINE1 TNC IRS2 AQP3 FRAT1 FRAT2 GJA5 MYC SALL4 DPF1 HOXA5 KIRREL3 EPHA4 UNC5B OSR1 ARID5B BHLHA15 POU3F1 MYT1 GEM EREG MFNG TXNIP ULK1 TRIM15 TAGLN3 FREM2 FLG SEMA7A PKDCC TSHZ1 STC1 GATA2 OTX1 PPARGC1A HOXC6 EGR1 FZD2 FZD4 PTCH1 LRRN4 NR0B2 ARHGAP24 POU6F1 EFNA1 NR4A2 IGSF10 GJB2 TMIE BCL6 FGF19 CYP1A1 SPRR1A SPRR1B CDKN1C GPSM1 CSRNP1 CDKN1A BTG2 PTPRR GAL3ST1 CXCL8 CITED2 SLC40A1 CHD6 SECTM1 FASLG PTPRJ SOBP GLI2 LFNG SPRED3 SIX5 ZNF488 EID2B SNTA1 EDN1 PTBP3 ITPKB TGFBR3 SLC7A5 ALDH1A3 AHRR PDE5A RAPGEF5 IVL MDFI KDM7A HBEGF CHST3 SPRR3 CATSPER2 SRF PTGS2 DLL4 ERBB3 SLITRK6 SH2B3 ATOH1 CDX2 WNT7B GRHL3 CCK ACVR2B APLN GATM EPGN DHRS9 KRT17 FMOD FRK*
48568	embryonic organ development	2.58E-07	1.27E-04	19/387 4.9%	215/17777 1.2%	*FLG EDN1 PKDCC CDX2 CITED2 WNT7B TSHZ1 OSR1 GATA2 SOBP GLI2 EFNA1 ALDH1A3 TMIE OTX1 SH2B3 HOXA5 MDFI ATOH1*
50794	regulation of cellular process	3.14E-07	1.35E-04	183/387 47.2%	6218/17777 34.9%	*ERRFI1 VIPR1 CSF2 TNFRSF6B ZBTB24 SERPINE1 GUCA1B TNC RORC JADE1 IRS2 CBLB KNDC1 AQP3 NR3C2 ARHGAP42 SESN3 MYC SESN1 SALL4 MYB LIPG DPF1 PDK4 TNFSF10 FBXO8 PHLDA1 ZNF565 HOXA5 AGFG2 PRKCG EPHA4 PDIA2 UNC5B KMT5C MAMSTR STARD8 OSR1 ARID5B DLEC1 SOX12 BHLHA15 POU3F1 MYT1 CPT1B GEM EREG KAT6B DDIT4 TXNIP ULK1 ZNF552 PTGES FLG CD274 ZNF792 GRB7 ZNF395 PKDCC TXK TSHZ1 KRBA2 GATA2 RHOBTB1 PDZD3 NUAK2 OTX1 PPARGC1A HOXC6 ABCA1 EGR1 FZD2 SNAI3 PTCH1 CBX2 HTR1D ZBTB10 CIDEB NR0B2 ELL2 ARHGAP24 POU6F1 FOSL1 EFNA1 NR4A2 SEPSECS ARHGAP31 VNN1 DLG2 PROC BCL6 TRPV6 AGO4 GKAP1 FGF19 CCDC88C CAPRIN2 JMY CYP1A1 SP5 HBP1 CALM2 HIST1H4D FGFR2 MXD4 CDKN1C ZNF512B GPSM1 CSRNP1 CDKN1A BTG2 CXCL8 CITED2 CITED4 HNF4G CHD6 SECTM1 TNFAIP3 FASLG AREG GLI2 AKAP12*

						SPRED3 SIX5 RASGEF1B ANKRD1 JAK3 ZNF488 ZNF763 EID2B TIGD3 KDM6B ZNF362 EDN1 FOXD2 TNFRSF1B MTSS1 ITPKB ARL4C TGFBR3 ALDH1A3 CNKSR3 IRF2 AHRR PDE5A RAPGEF5 MDFI KDM7A HBEGF CHST3 ZNF233 CREB5 INPP1 SRF RGS16 ADCY4 PTGS2 ADCY5 FGD2 DLL4 GNA14 ERBB3 DNAJB5 TP53INP1 GPAT3 BRD8 ATOH1 MAP3K3 CDX2 CCL20 GDF15 WNT7B GRHL3 CCK ACVR2B PNRC1 APLN EPGN ZNF618 ZYX RHEBL1 FRK BNIPL
7166	cell surface receptor linked signaling pathway	4.06E-07	1.55E-04	56/387 14.4%	1279/17777 7.1%	*CSRNP1 VIPR1 CXCL8 GUCA1B IRS2 CBLB PTPRJ AREG GLI2 AKAP12 FRAT1 FRAT2 SOSTDC1 EPHA4 EDN1 GPR37 ARID5B BHLHA15 TNFRSF1B MTSS1 GEM EREG ITPKB TGFBR3 GIGYF1 TXNIP HBEGF FLG CD274 GRB7 ADCY4 STC1 ADCY5 NKD2 DLL4 GNA14 PDZD3 ERBB3 BRD8 ABCA1 GPR17 FZD2 FZD4 GDF15 WNT7B PTCH1 HTR1D CCK ACVR2B EFNA1 FGF19 CCDC88C HBP1 FMOD CALM2 FGFR2*
9887	organ morphogenesis	4.71E-07	1.62E-04	35/387 9.0%	634/17777 3.5%	*FLG ERRFI1 CSRNP1 CDKN1A SRF TSHZ1 TNC FASLG SOBP AQP3 GLI2 LFNG ERBB3 MYC OTX1 HOXA5 ATOH1 EDN1 CDX2 WNT7B OSR1 PTCH1 ARID5B NR0B2 ACVR2B GEM EREG EFNA1 TGFBR3 ALDH1A3 TMIE TRIM15 FMOD FREM2 MDFI*
50789	regulation of biological process	1.20E-06	3.76E-04	188/387 48.5%	6548/17777 36.8%	*ERRFI1 VIPR1 CSF2 TNFRSF6B ZBTB24 SERPINE1 GUCA1B TNC RORC JADE1 IRS2 CBLB KNDC1 BEST3 AQP3 NR3C2 ARHGAP42 SESN3 MYC SESN1 SALL4 MYB LIPG DPF1 PDK4 TNFSF10 FBXO8 PHLDA1 ZNF565 HOXA5 AGFG2 PRKCG EPHA4 PDIA2 UNC5B KMT5C MAMSTR STARD8 OSR1 DIO3 ARID5B DLEC1 SOX12 BHLHA15 POU3F1 MYT1 CPT1B GEM EREG KAT6B DDIT4 TXNIP ULK1 ZNF552 PTGES FLG CD274 ZNF792 GRB7 ZNF395 PKDCC KCNE3 TXK TSHZ1 KRBA2 GATA2 RHOBTB1 C2 PDZD3 NUAK2 OTX1 PPARGC1A HOXC6 ABCA1 EGR1 FZD2 SNAI3 PTCH1 CBX2 HTR1D ZBTB10 CIDEB NR0B2 ELL2 ARHGAP24 POU6F1 FOSL1 EFNA1 NR4A2 SEPSECS ARHGAP31 VNN1 DLG2 PROC BCL6*

						TRPV6 AGO4 GKAP1 FGF19 CCDC88C CAPRIN2 JMY CYP1A1 SP5 HBP1 CALM2 HIST1H4D FGFR2 MXD4 CDKN1C ZNF512B GPSM1 CSRNP1 CDKN1A BTG2 CXCL8 CITED2 CITED4 HNF4G CHD6 SECTM1 TNFAIP3 FASLG AREG GLI2 AKAP12 SPRED3 H2AFJ SIX5 RASGEF1B ANKRD1 JAK3 ZNF488 ZNF763 EID2B TIGD3 KDM6B ZNF362 EDN1 FOXD2 TNFRSF1B MTSS1 ITPKB ARL4C TGFBR3 ALDH1A3 CNKSR3 IRF2 AHRR PDE5A RAPGEF5 MDFI KDM7A HBEGF CHST3 ZNF233 CREB5 INPP1 SRF RGS16 ADCY4 PTGS2 ADCY5 FGD2 DLL4 GNA14 ERBB3 DNAJB5 TP53INP1 GPAT3 BRD8 ATOH1 MAP3K3 CDX2 CCL20 GDF15 WNT7B GRHL3 CCK ACVR2B PNRC1 APLN EPGN ZNF618 ZYX RHEBL1 FRK BNIPL
65007	biological regulation	1.82E-06	5.21E-04	196/387 50.6%	6937/17777 39.0%	*ERRFI1 VIPR1 TNFRSF6B SERPINE1 CBLB AQP3 MYC MYB LIPG DPF1 PDK4 TNFSF10 CYP1B1 FBXO8 HOXA5 AGFG2 PRKCG EPHA4 UNC5B STARD8 DLEC1 SOX12 BHLHA15 CPT1B EREG DDIT4 TXNIP ULK1 PTGES FLG GRB7 ZNF395 TXK TSHZ1 STC1 GATA2 C2 NUAK2 PPARGC1A HOXC6 ABCA1 EGR1 FZD2 SNAI3 CIDEB NR0B2 ELL2 POU6F1 FOSL1 VNN1 AGO4 GKAP1 CCDC88C CYP1A1 HBP1 CALM2 HIST1H4D CDKN1C CSRNP1 CDKN1A BTG2 CITED2 CITED4 HNF4G SECTM1 AREG GLI2 AKAP12 ANKRD1 JAK3 ZNF488 TIGD3 ZNF362 EDN1 FOXD2 MTSS1 ITPKB CNKSR3 AHRR RAPGEF5 MDFI HBEGF CHST3 ZNF233 INPP1 ADCY4 PTGS2 ADCY5 FGD2 GPAT3 BRD8 MAP3K3 CDX2 CCL20 GDF15 WNT7B GAD1 GRHL3 EPGN DHRS9 ZYX FRK CSF2 ZBTB24 GUCA1B TNC RORC JADE1 IRS2 KNDC1 BEST3 NR3C2 ARHGAP42 SESN3 SESN1 SALL4 PHLDA1 ZNF565 PDIA2 KMT5C MAMSTR OSR1 DIO3 ARID5B POU3F1 MYT1 GEM KAT6B ZNF552 CD274 ZNF792 PKDCC KCNE3 KRBA2 RHOBTB1 PDZD3 OTX1 PTCH1 CBX2 HTR1D ZBTB10 ARHGAP24 EFNA1 NR4A2 SEPSECS ARHGAP31 DLG2 PROC BCL6 TRPV6 FGF19 CAPRIN2 JMY SP5 FGFR2 MXD4 ZNF512B GPSM1 GAL3ST1 CXCL8 SLC40A1 CHD6 TNFAIP3 FASLG SPRED3 H2AFJ SIX5*

						RASGEF1B ZNF763 EID2B KDM6B TNFRSF1B ARL4C TGFBR3 ALDH1A3 IRF2 PDE5A KDM7A CREB5 SRF RGS16 DLL4 GNA14 ERBB3 DNAJB5 TP53INP1 BCO2 ATOH1 HSD3B1 CCK ACVR2B PNRC1 APLN ZNF618 RHEBL1 BNIPL
7167	enzyme linked receptor protein signaling pathway	2.29E-06	6.06E-04	23/387 5.9%	346/17777 1.9%	*FLG EPHA4 GRB7 CSRNP1 GDF15 GUCA1B ARID5B IRS2 PTPRJ AREG ACVR2B MTSS1 EREG EFNA1 TGFBR3 GIGYF1 PDZD3 ERBB3 FGF19 TXNIP FMOD FGFR2 HBEGF*
48523	negative regulation of cellular process	2.49E-06	6.13E-04	70/387 18.0%	1844/17777 10.3%	*CDKN1C ERRFI1 CDKN1A BTG2 CSF2 TNFRSF6B CXCL8 CITED2 SERPINE1 JADE1 TNFAIP3 IRS2 CBLB GLI2 SESN3 SIX5 MYC SESN1 SALL4 HOXA5 PRKCG KDM6B EDN1 OSR1 ARID5B DLEC1 GEM EREG TGFBR3 KAT6B DDIT4 CNKSR3 IRF2 TXNIP ULK1 MDFI HBEGF PTGES FLG CD274 PKDCC RGS16 PTGS2 DLL4 PDZD3 NUAK2 ERBB3 DNAJB5 TP53INP1 ABCA1 EGR1 CDX2 PTCH1 CBX2 CCK NR0B2 FOSL1 NR4A2 VNN1 PROC BCL6 AGO4 FGF19 CAPRIN2 JMY HBP1 HIST1H4D FRK MXD4 BNIPL*
23052	signaling	2.99E-06	6.46E-04	104/387 26.8%	3130/17777 17.6%	*VIPR1 GUCA1B TNC IRS2 CBLB NR3C2 FRAT1 ARHGAP42 FRAT2 GJA5 PDK4 TNFSF10 HOXA5 EPHA4 UNC5B STARD8 OSR1 ARID5B BHLHA15 POU3F1 GEM EREG TXNIP ULK1 PTGES FLG CD274 GRB7 TXK STC1 EFNA4 RHOBTB1 PDZD3 PPARGC1A ABCA1 GPR17 FZD2 FZD4 PTCH1 HTR1D CIDEB NR0B2 ARHGAP24 EFNA1 NR4A2 GJB2 EFNA3 ARHGAP31 DLG2 GKAP1 FGF19 CCDC88C HBP1 CALM2 HIST1H4D FGFR2 GPSM1 CSRNP1 CDKN1A GAL3ST1 CXCL8 FASLG PTPRJ AREG GLI2 AKAP12 SPRED3 ANKRD1 SOSTDC1 JAK3 EDN1 GPR37 TNFRSF1B MTSS1 ITPKB ARL4C TGFBR3 GIGYF1 PDE5A RAPGEF5 MDFI HBEGF INPP1 ADCY4 ADCY5 NKD2 DLL4 GNA14 ERBB3 BRD8 SH2B3 MAP3K3 CCL20 GDF15 WNT7B GAD1 CCK ACVR2B APLN EPGN NOVA1 ZYX FMOD RHEBL1*

9888	tissue development	3.01E-06	6.46E-04	37/387 9.5%	748/17777 4.2%	*FLG ERRFI1 SPRR3 CITED2 SRF SERPINE1 TNC SECTM1 PTGS2 GLI2 ERBB3 MYC SALL4 HOXA5 SNTA1 EDN1 FZD2 WNT7B OSR1 PTCH1 GRHL3 POU3F1 ACVR2B EREG TGFBR3 IGSF10 ALDH1A3 GATM DHRS9 KRT17 FGF19 TXNIP TRIM15 SPRR1A FREM2 IVL SPRR1B*
7154	cell communication	3.25E-06	6.58E-04	39/387 10.0%	812/17777 4.5%	*FLG CDKN1A VIPR1 GAL3ST1 GUCA1B TNC STC1 FASLG PTPRJ AREG EFNA4 GLI2 DLL4 NUAK2 GJA5 TNFSF10 ENPP5 HOXA5 EDN1 FZD2 FZD4 CCL20 GDF15 WNT7B GAD1 HTR1D BHLHA15 POU3F1 ACVR2B EREG FOSL1 EFNA1 NR4A2 GJB2 SNX18 EFNA3 NOVA1 ZYX FREM2*
48519	negative regulation of biological process	4.38E-06	8.37E-04	74/387 19.1%	2019/17777 11.3%	*CDKN1C ERRFI1 CDKN1A BTG2 CSF2 TNFRSF6B CXCL8 CITED2 SERPINE1 JADE1 TNFAIP3 IRS2 FASLG CBLB BEST3 GLI2 SESN3 SIX5 MYC SESN1 SALL4 HOXA5 PRKCG KDM6B EDN1 OSR1 ARID5B DLEC1 TNFRSF1B GEM EREG TGFBR3 KAT6B DDIT4 CNKSR3 IRF2 TXNIP ULK1 MDFI HBEGF PTGES FLG CD274 PKDCC RGS16 PTGS2 DLL4 PDZD3 NUAK2 ERBB3 DNAJB5 TP53INP1 ABCA1 EGR1 CDX2 PTCH1 CBX2 CCK NR0B2 FOSL1 NR4A2 VNN1 PROC BCL6 AGO4 FGF19 CAPRIN2 JMY HBP1 CALM2 HIST1H4D FRK MXD4 BNIPL*
60429	epithelium development	4.86E-06	8.69E-04	22/387 5.6%	336/17777 1.8%	*FLG ERRFI1 SPRR3 EDN1 FZD2 WNT7B SRF OSR1 PTCH1 TNC POU3F1 GLI2 EREG ALDH1A3 DHRS9 MYC TXNIP SPRR1A HOXA5 FREM2 IVL SPRR1B*
6464	protein modification process	5.05E-06	8.69E-04	60/387 15.5%	1526/17777 8.5%	*CDKN1A BTG2 PTPRR GAL3ST1 JADE1 TESK2 TNFAIP3 PTPRJ PDK4 JAK3 PCMTD1 PRKCG KDM6B DUSP5 EPHA4 CDKL5 KMT5C OSR1 MAMDC2 MYT1 TGFBR3 RNF144B B3GNT8 KAT6B PADI3 PADI2 ULK1 PDZRN3 PADI1 IVL MDFI KDM7A FLG LIPT2 OTUB2 PKDCC TXK PPM1K FUT2 GNA14 NUAK2 ERBB3 ST3GAL4 BRD8 ABCA1 MAP3K3 GAD1 B3GALT4 CCK ACVR2B EFNA1 MEX3B EPGN MGAT4A LNX1 PTPN7 SPRR1A FRK SPRR1B FGFR2*

7169	transmembrane receptor protein tyrosine kinase signaling pathway	6.48E-06	1.06E-03	17/387 4.3%	219/17777 1.2%	*FLG EPHA4 GRB7 CSRNP1 ARID5B IRS2 PTPRJ AREG MTSS1 EREG EFNA1 GIGYF1 ERBB3 FGF19 TXNIP FGFR2 HBEGF*
9790	embryonic development	8.96E-06	1.39E-03	31/387 8.0%	602/17777 3.3%	*FLG PTPRR PKDCC CITED2 SRF TSHZ1 GATA2 SOBP GLI2 FRAT1 GJA5 SALL4 OTX1 SH2B3 HOXA5 ATOH1 HOXC6 EDN1 CDX2 WNT7B OSR1 PTCH1 ACVR2B EFNA1 ALDH1A3 GATM TMIE CYP1A1 AHRR TRIM15 MDFI*
23033	signaling pathway	9.28E-06	1.39E-03	75/387 19.3%	2099/17777 11.8%	*CSRNP1 CDKN1A VIPR1 CXCL8 GUCA1B IRS2 CBLB PTPRJ AREG GLI2 AKAP12 SPRED3 FRAT1 FRAT2 SOSTDC1 JAK3 EPHA4 EDN1 GPR37 OSR1 ARID5B BHLHA15 TNFRSF1B MTSS1 GEM EREG ITPKB ARL4C TGFBR3 GIGYF1 TXNIP ULK1 RAPGEF5 MDFI HBEGF FLG CD274 GRB7 TXK ADCY4 STC1 RHOBTB1 ADCY5 NKD2 DLL4 GNA14 PDZD3 ERBB3 BRD8 PPARGC1A SH2B3 ABCA1 GPR17 MAP3K3 FZD2 FZD4 GDF15 WNT7B PTCH1 HTR1D CIDEB CCK NR0B2 ACVR2B EFNA1 EPGN ARHGAP31 FGF19 CCDC88C HBP1 FMOD RHEBL1 CALM2 HIST1H4D FGFR2*
30154	cell differentiation	1.02E-05	1.47E-03	63/387 16.2%	1668/17777 9.3%	*CDKN1C GPSM1 BTG2 PTPRR CSF2 CITED2 TNC FASLG PTPRJ GLI2 SIX5 ZNF488 HOXA5 EID2B SNTA1 EPHA4 EDN1 UNC5B BHLHA15 POU3F1 MYT1 EREG ITPKB TGFBR3 SLC7A5 TXNIP AHRR ULK1 TRIM15 IVL MDFI CHST3 FLG SPRR3 SEMA7A CATSPER2 PKDCC SRF GATA2 DLL4 ERBB3 SLITRK6 PPARGC1A SH2B3 ATOH1 EGR1 FZD2 CDX2 FZD4 WNT7B PTCH1 CCK ARHGAP24 EFNA1 NR4A2 IGSF10 DHRS9 BCL6 FGF19 CYP1A1 SPRR1A FRK SPRR1B*
48562	embryonic organ morphogenesis	1.19E-05	1.64E-03	13/387 3.3%	140/17777 0.7%	*FLG EDN1 TSHZ1 OSR1 SOBP GLI2 EFNA1 ALDH1A3 TMIE OTX1 HOXA5 MDFI ATOH1*
42471	ear morphogenesis	1.24E-05	1.64E-03	9/387 2.3%	66/17777 0.3%	*FLG TMIE EDN1 TSHZ1 OSR1 OTX1 SOBP ATOH1 GLI2*

9913	epidermal cell differentiation	1.59E-05	2.02E-03	9/387 2.3%	68/17777 0.3%	*FLG SPRR3 TXNIP POU3F1 SPRR1A IVL SPRR1B GLI2 EREG*
7165	signal transduction	1.70E-05	2.09E-03	68/387 17.5%	1877/17777 10.5%	*GPSM1 CDKN1A VIPR1 CXCL8 GUCA1B TNC IRS2 FASLG CBLB NR3C2 AKAP12 SPRED3 ARHGAP42 PDK4 TNFSF10 ANKRD1 JAK3 EPHA4 EDN1 UNC5B STARD8 OSR1 BHLHA15 MTSS1 GEM EREG ITPKB ARL4C TGFBR3 ULK1 PDE5A RAPGEF5 MDFI HBEGF PTGES FLG CD274 GRB7 INPP1 TXK ADCY4 RHOBTB1 ADCY5 DLL4 GNA14 PDZD3 ERBB3 BRD8 ABCA1 MAP3K3 FZD2 CCL20 GDF15 PTCH1 HTR1D CCK ACVR2B ARHGAP24 APLN EFNA1 NR4A2 EPGN ARHGAP31 DLG2 GKAP1 ZYX RHEBL1 HIST1H4D*
48869	cellular developmental process	2.38E-05	2.78E-03	63/387 16.2%	1714/17777 9.6%	*CDKN1C GPSM1 BTG2 PTPRR CSF2 CITED2 TNC FASLG PTPRJ GLI2 SIX5 ZNF488 HOXA5 EID2B SNTA1 EPHA4 EDN1 UNC5B BHLHA15 POU3F1 MYT1 EREG ITPKB TGFBR3 SLC7A5 TXNIP AHRR ULK1 TRIM15 IVL MDFI CHST3 FLG SPRR3 SEMA7A CATSPER2 PKDCC SRF GATA2 DLL4 ERBB3 SLITRK6 PPARGC1A SH2B3 ATOH1 EGR1 FZD2 CDX2 FZD4 WNT7B PTCH1 CCK ARHGAP24 EFNA1 NR4A2 IGSF10 DHRS9 BCL6 FGF19 CYP1A1 SPRR1A FRK SPRR1B*
43412	macromolecule modification	2.42E-05	2.78E-03	60/387 15.5%	1607/17777 9.0%	*CDKN1A BTG2 PTPRR GAL3ST1 JADE1 TESK2 TNFAIP3 PTPRJ PDK4 JAK3 PCMTD1 PRKCG KDM6B DUSP5 EPHA4 CDKL5 KMT5C OSR1 MAMDC2 MYT1 TGFBR3 RNF144B B3GNT8 KAT6B PADI3 PADI2 ULK1 PDZRN3 PADI1 IVL MDFI KDM7A FLG LIPT2 OTUB2 PKDCC TXK PPM1K FUT2 GNA14 NUAK2 ERBB3 ST3GAL4 BRD8 ABCA1 MAP3K3 GAD1 B3GALT4 CCK ACVR2B EFNA1 MEX3B EPGN MGAT4A LNX1 PTPN7 SPRR1A FRK SPRR1B FGFR2*
7389	pattern specification process	2.93E-05	3.25E-03	18/387 4.6%	271/17777 1.5%	*BTG2 EDN1 CDX2 CITED2 WNT7B SRF TSHZ1 PTCH1 GRHL3 ACVR2B GLI2 LFNG FRAT1 MFNG OTX1 HOXA5 MDFI HOXC6*

42573	retinoic acid metabolic process	3.23E-05	3.47E-03	5/387 1.2%	18/17777 0.1%	*ALDH1A3 DHRS9 CYP1A1 AHRR BCO2*
60021	palate development	3.60E-05	3.75E-03	7/387 1.8%	43/17777 0.2%	*TGFBR3 CSRNP1 PKDCC TSHZ1 OSR1 ARID5B ACVR2B*
48598	embryonic morphogenesis	5.10E-05	5.09E-03	20/387 5.1%	336/17777 1.8%	*FLG EDN1 WNT7B SRF TSHZ1 OSR1 PTCH1 SOBP ACVR2B GLI2 EFNA1 ALDH1A3 TMIE GJA5 SALL4 TRIM15 OTX1 HOXA5 MDFI ATOH1*
48518	positive regulation of biological process	5.18E-05	5.09E-03	75/387 19.3%	2207/17777 12.4%	*CDKN1C CSRNP1 CDKN1A BTG2 VIPR1 CSF2 CXCL8 CITED2 SERPINE1 CITED4 SECTM1 IRS2 FASLG CBLB AREG AQP3 GLI2 AKAP12 MYC SALL4 LIPG DPF1 TNFSF10 ZNF488 PHLDA1 HOXA5 PRKCG EDN1 OSR1 DIO3 BHLHA15 POU3F1 EREG ITPKB ALDH1A3 KAT6B CNKSR3 TXNIP AHRR PDE5A HBEGF CHST3 CREB5 FLG PKDCC SRF GATA2 PTGS2 C2 FGD2 ERBB3 TP53INP1 PPARGC1A ATOH1 ABCA1 EGR1 MAP3K3 CDX2 WNT7B CIDEB GRHL3 CCK NR0B2 ACVR2B FOSL1 NR4A2 EPGN VNN1 BCL6 FGF19 JMY CYP1A1 CALM2 FGFR2 BNIPL*
32501	multicellular organismal process	5.88E-05	5.50E-03	129/387 33.3%	4374/17777 24.6%	*ERRFI1 VIPR1 CSF2 SERPINE1 GUCA1B TNC TESK2 IRS2 AQP3 FRAT1 FRAT2 GJA5 MYC SALL4 DPF1 CYP1B1 HOXA5 PRKCG KIRREL3 EPHA4 UNC5B OSR1 ARID5B CACNA2D4 POU3F1 MYT1 GEM EREG MFNG TXNIP ULK1 TRIM15 TAGLN3 FREM2 FLG SEMA7A PKDCC TSHZ1 STC1 GATA2 SPATA7 OTX1 PPARGC1A HOXC6 ABCA1 EGR1 FZD2 FZD4 PTCH1 HTR1D LRRN4 NR0B2 ARHGAP24 POU6F1 FOSL1 EFNA1 NR4A2 IGSF10 OCLM GJB2 TMIE BCL6 CTRL FGF19 CYP1A1 CALM2 SPRR1A SPRR1B CDKN1C GPSM1 CSRNP1 CDKN1A BTG2 PTPRR GAL3ST1 CXCL8 CITED2 CHD6 SECTM1 FASLG PTPRJ SOBP GLI2 LFNG SPRED3 SIX5 ZNF488 EID2B SNTA1 EDN1 BAAT MUC5AC ITPKB TGFBR3 SLC7A5 ALDH1A3 MMP13 AHRR PDE5A RAPGEF5 IVL MDFI KDM7A HBEGF CHST3 SPRR3 CATSPER2 SRF RGS16 PTGS2 ADCY5 DLL4 ERBB3 SLITRK6 SH2B3 ATOH1 CDX2 WNT7B GAD1 GRHL3 CCK ACVR2B APLN GATM EPGN*

						DHRS9 KRT17 NOVA1 FMOD
8544	epidermis development	5.91E-05	5.50E-03	14/387 3.6%	186/17777 1.0%	*FLG ERRFI1 SPRR3 PTCH1 GRHL3 PTGS2 POU3F1 GLI2 EREG KRT17 TXNIP SPRR1A IVL SPRR1B*
30216	keratinocyte differentiation	6.39E-05	5.78E-03	8/387 2.0%	63/17777 0.3%	*FLG SPRR3 TXNIP POU3F1 SPRR1A IVL SPRR1B EREG*
9991	response to extracellular stimulus	7.48E-05	6.60E-03	17/387 4.3%	265/17777 1.4%	*CDKN1A FZD4 WNT7B PTCH1 STC1 CCK PTGS2 SUOX AQP3 FOSL1 NR4A2 GATM NUAK2 LIPG CYP1A1 AHRR PPARGC1A*
30855	epithelial cell differentiation	8.15E-05	7.01E-03	13/387 3.3%	168/17777 0.9%	*FLG SPRR3 FZD2 WNT7B PTCH1 POU3F1 EREG DHRS9 TXNIP SPRR1A HOXA5 IVL SPRR1B*
31326	regulation of cellular biosynthetic process	8.54E-05	7.10E-03	95/387 24.5%	3021/17777 16.9%	*CDKN1C ZNF512B CSRNP1 BTG2 CSF2 ZBTB24 CITED2 CITED4 GUCA1B RORC HNF4G JADE1 CHD6 IRS2 AREG GLI2 NR3C2 AKAP12 SIX5 MYC SALL4 MYB DPF1 PDK4 ANKRD1 ZNF488 ZNF565 ZNF763 HOXA5 EID2B TIGD3 KDM6B ZNF362 EDN1 FOXD2 KMT5C MAMSTR ARID5B SOX12 BHLHA15 POU3F1 MYT1 GEM EREG KAT6B IRF2 TXNIP AHRR MDFI KDM7A HBEGF ZNF233 ZNF552 CREB5 ZNF792 ZNF395 SRF TSHZ1 ADCY4 KRBA2 GATA2 PTGS2 ADCY5 FGD2 PDZD3 DNAJB5 BRD8 OTX1 PPARGC1A ATOH1 HOXC6 ABCA1 EGR1 CDX2 SNAI3 CBX2 ZBTB10 GRHL3 CCK NR0B2 ELL2 ACVR2B PNRC1 POU6F1 FOSL1 NR4A2 SEPSECS BCL6 ZNF618 AGO4 FGF19 JMY SP5 RHEBL1 MXD4*
9636	response to toxin	8.95E-05	7.10E-03	8/387 2.0%	66/17777 0.3%	*NR4A2 CDKN1A PDZD3 SRF HTR1D CYP1A1 AHRR SLC7A11*
7267	cell-cell signaling	9.33E-05	7.10E-03	28/387 7.2%	587/17777 3.3%	*FLG VIPR1 GUCA1B TNC STC1 FASLG PTPRJ AREG EFNA4 GLI2 GJA5 TNFSF10 HOXA5 EDN1 FZD2 CCL20 GDF15 WNT7B GAD1 HTR1D BHLHA15 ACVR2B EREG EFNA1 GJB2 EFNA3 NOVA1 ZYX*

18101	peptidyl-citrulline biosynthetic process from peptidyl-arginine	9.91E-05	7.10E-03	3/387 0.7%	5/17777 0.0%	*PADI3 PADI2 PADI1*
42904	9-cis-retinoic acid biosynthetic process	9.91E-05	7.10E-03	3/387 0.7%	5/17777 0.0%	*DHRS9 CYP1A1 AHRR*
42905	9-cis-retinoic acid metabolic process	9.91E-05	7.10E-03	3/387 0.7%	5/17777 0.0%	*DHRS9 CYP1A1 AHRR*
35238	vitamin A biosynthetic process	9.91E-05	7.10E-03	3/387 0.7%	5/17777 0.0%	*DHRS9 CYP1A1 AHRR*
4668	protein-arginine deiminase activity	9.91E-05	7.10E-03	3/387 0.7%	5/17777 0.0%	*PADI3 PADI2 PADI1*
7507	heart development	1.05E-04	7.40E-03	15/387 3.8%	221/17777 1.2%	*EDN1 CITED2 SRF OSR1 PTCH1 TNC PTPRJ ACVR2B POU6F1 GLI2 TGFBR3 ERBB3 GJA5 SALL4 FGF19*
7423	sensory organ development	1.08E-04	7.45E-03	16/387 4.1%	247/17777 1.3%	*FLG EDN1 WNT7B TSHZ1 OSR1 FASLG SOBP GLI2 ALDH1A3 TMIE ERBB3 SIX5 CYP1A1 AHRR OTX1 ATOH1*
9889	regulation of biosynthetic process	1.15E-04	7.76E-03	95/387 24.5%	3045/17777 17.1%	*CDKN1C ZNF512B CSRNP1 BTG2 CSF2 ZBTB24 CITED2 CITED4 GUCA1B RORC HNF4G JADE1 CHD6 IRS2 AREG GLI2 NR3C2 AKAP12 SIX5 MYC SALL4 MYB DPF1 PDK4 ANKRD1 ZNF488 ZNF565 ZNF763 HOXA5 EID2B TIGD3 KDM6B ZNF362 EDN1 FOXD2 KMT5C MAMSTR ARID5B SOX12 BHLHA15 POU3F1 MYT1 GEM EREG KAT6B IRF2 TXNIP AHRR MDFI KDM7A HBEGF ZNF233 ZNF552 CREB5 ZNF792 ZNF395 SRF TSHZ1 ADCY4 KRBA2 GATA2 PTGS2 ADCY5 FGD2 PDZD3 DNAJB5 BRD8 OTX1 PPARGC1A ATOH1 HOXC6 ABCA1 EGR1 CDX2 SNAI3 CBX2 ZBTB10 GRHL3 CCK NR0B2 ELL2 ACVR2B PNRC1 POU6F1 FOSL1 NR4A2 SEPSECS BCL6 ZNF618 AGO4 FGF19 JMY SP5 RHEBL1 MXD4*

43687	post-translational protein modification	1.20E-04	7.95E-03	48/387 12.4%	1266/17777 7.1%	*FLG OTUB2 CDKN1A BTG2 PTPRR PKDCC TXK JADE1 TESK2 TNFAIP3 PTPRJ PPM1K GNA14 NUAK2 ERBB3 PDK4 BRD8 JAK3 PRKCG KDM6B DUSP5 EPHA4 MAP3K3 CDKL5 KMT5C GAD1 OSR1 MAMDC2 CCK MYT1 ACVR2B EFNA1 TGFBR3 RNF144B MEX3B EPGN KAT6B LNX1 ULK1 PTPN7 SPRR1A PDZRN3 IVL MDFI FRK SPRR1B FGFR2 KDM7A*
23046	signaling process	1.30E-04	8.31E-03	72/387 18.6%	2156/17777 12.1%	*GPSM1 CDKN1A VIPR1 GAL3ST1 CXCL8 GUCA1B TNC IRS2 FASLG CBLB NR3C2 AKAP12 SPRED3 ARHGAP42 PDK4 TNFSF10 ANKRD1 JAK3 EPHA4 EDN1 UNC5B STARD8 OSR1 BHLHA15 POU3F1 MTSS1 GEM EREG ITPKB ARL4C TGFBR3 ULK1 PDE5A RAPGEF5 MDFI HBEGF PTGES FLG CD274 GRB7 INPP1 TXK ADCY4 RHOBTB1 ADCY5 DLL4 GNA14 PDZD3 ERBB3 BRD8 ABCA1 MAP3K3 FZD2 CCL20 GDF15 GAD1 PTCH1 HTR1D CCK ACVR2B ARHGAP24 APLN EFNA1 NR4A2 EPGN ARHGAP31 DLG2 NOVA1 GKAP1 ZYX RHEBL1 HIST1H4D*
23060	signal transmission	1.30E-04	8.31E-03	72/387 18.6%	2156/17777 12.1%	*GPSM1 CDKN1A VIPR1 GAL3ST1 CXCL8 GUCA1B TNC IRS2 FASLG CBLB NR3C2 AKAP12 SPRED3 ARHGAP42 PDK4 TNFSF10 ANKRD1 JAK3 EPHA4 EDN1 UNC5B STARD8 OSR1 BHLHA15 POU3F1 MTSS1 GEM EREG ITPKB ARL4C TGFBR3 ULK1 PDE5A RAPGEF5 MDFI HBEGF PTGES FLG CD274 GRB7 INPP1 TXK ADCY4 RHOBTB1 ADCY5 DLL4 GNA14 PDZD3 ERBB3 BRD8 ABCA1 MAP3K3 FZD2 CCL20 GDF15 GAD1 PTCH1 HTR1D CCK ACVR2B ARHGAP24 APLN EFNA1 NR4A2 EPGN ARHGAP31 DLG2 NOVA1 GKAP1 ZYX RHEBL1 HIST1H4D*
48468	cell development	1.37E-04	8.57E-03	29/387 7.4%	632/17777 3.5%	*FLG CDKN1C SRF TNC FASLG GATA2 GLI2 ERBB3 SIX5 SLITRK6 ZNF488 HOXA5 ATOH1 SNTA1 EPHA4 EDN1 CDX2 UNC5B WNT7B CCK BHLHA15 POU3F1 EREG NR4A2 TGFBR3 BCL6 FGF19 ULK1 CHST3*

7398	ectoderm development	1.43E-04	8.78E-03	14/387 3.6%	202/17777 1.1%	*FLG ERRFI1 SPRR3 PTCH1 GRHL3 PTGS2 POU3F1 GLI2 EREG KRT17 TXNIP SPRR1A IVL SPRR1B*
40007	growth	1.94E-04	1.14E-02	14/387 3.6%	208/17777 1.1%	*FLG WNT7B SERPINE1 ARID5B PTGS2 ACVR2B GLI2 EREG TGFBR3 IGSF10 GATM ULK1 HOXA5 FGFR2*
42362	fat-soluble vitamin biosynthetic process	1.95E-04	1.14E-02	3/387 0.7%	6/17777 0.0%	*DHRS9 CYP1A1 AHRR*
19240	citrulline biosynthetic process	1.95E-04	1.14E-02	3/387 0.7%	6/17777 0.0%	*PADI3 PADI2 PADI1*
6357	regulation of transcription from RNA polymerase II promoter	2.18E-04	1.24E-02	32/387 8.2%	747/17777 4.2%	*CDKN1C CSRNP1 CITED2 SRF HNF4G GATA2 GLI2 DNAJB5 MYC SALL4 ANKRD1 BRD8 OTX1 PPARGC1A HOXA5 ATOH1 HOXC6 KDM6B EGR1 CDX2 CBX2 SOX12 BHLHA15 NR0B2 FOSL1 NR4A2 BCL6 JMY IRF2 TXNIP MDFI MXD4*
42127	regulation of cell proliferation	2.19E-04	1.24E-02	35/387 9.0%	847/17777 4.7%	*FLG CDKN1C CD274 CDKN1A BTG2 VIPR1 CSF2 CXCL8 SERPINE1 IRS2 FASLG CBLB PTGS2 GLI2 ERBB3 SIX5 MYC SESN1 HOXA5 EDN1 PTCH1 DLEC1 CCK EREG FOSL1 TGFBR3 EPGN BCL6 FGF19 TXNIP FRK FGFR2 PTGES MXD4 BNIPL*
51171	regulation of nitrogen compound metabolic process	2.78E-04	1.54E-02	93/387 24.0%	3039/17777 17.0%	*CDKN1C ZNF512B CSRNP1 BTG2 CSF2 ZBTB24 CITED2 CITED4 GUCA1B TNC RORC HNF4G JADE1 CHD6 AREG GLI2 NR3C2 AKAP12 SIX5 MYC SALL4 MYB DPF1 ANKRD1 ZNF488 ZNF565 ZNF763 HOXA5 EID2B AGFG2 PRKCG TIGD3 KDM6B ZNF362 EDN1 FOXD2 KMT5C MAMSTR ARID5B SOX12 BHLHA15 TNFRSF1B POU3F1 MYT1 GEM EREG KAT6B IRF2 TXNIP AHRR MDFI KDM7A ZNF233 ZNF552 CREB5 ZNF792 ZNF395 SRF TSHZ1 ADCY4 KRBA2 GATA2 PTGS2 ADCY5 FGD2 PDZD3 DNAJB5 BRD8 OTX1 PPARGC1A ATOH1 HOXC6 ABCA1 EGR1 CDX2 SNAI3 CBX2 ZBTB10 GRHL3 CCK NR0B2 ELL2 ACVR2B PNRC1 POU6F1 FOSL1 NR4A2 BCL6 ZNF618 JMY SP5 RHEBL1 MXD4*

19219	regulation of nucleobase, nucleoside, nucleotide and nucleic acid metabolic process	3.27E-04	1.79E-02	92/387 23.7%	3013/17777 16.9%	*CDKN1C ZNF512B CSRNP1 BTG2 CSF2 ZBTB24 CITED2 CITED4 GUCA1B TNC RORC HNF4G JADE1 CHD6 AREG GLI2 NR3C2 AKAP12 SIX5 MYC SALL4 MYB DPF1 ANKRD1 ZNF488 ZNF565 ZNF763 HOXA5 EID2B AGFG2 PRKCG TIGD3 KDM6B ZNF362 EDN1 FOXD2 KMT5C MAMSTR ARID5B SOX12 BHLHA15 TNFRSF1B POU3F1 MYT1 GEM EREG KAT6B IRF2 TXNIP AHRR MDFI KDM7A ZNF233 ZNF552 CREB5 ZNF792 ZNF395 SRF TSHZ1 ADCY4 KRBA2 GATA2 ADCY5 FGD2 PDZD3 DNAJB5 BRD8 OTX1 PPARGC1A ATOH1 HOXC6 ABCA1 EGR1 CDX2 SNAI3 CBX2 ZBTB10 GRHL3 CCK NR0B2 ELL2 ACVR2B PNRC1 POU6F1 FOSL1 NR4A2 BCL6 ZNF618 JMY SP5 RHEBL1 MXD4*
52	citrulline metabolic process	3.36E-04	1.81E-02	3/387 0.7%	7/17777 0.0%	*PADI3 PADI2 PADI1*
19222	regulation of metabolic process	3.41E-04	1.81E-02	114/387 29.4%	3916/17777 22.0%	*ERRFI1 CSF2 ZBTB24 SERPINE1 GUCA1B TNC RORC JADE1 IRS2 CBLB NR3C2 MYC SALL4 MYB LIPG DPF1 PDK4 ZNF565 HOXA5 AGFG2 PRKCG KMT5C MAMSTR OSR1 ARID5B SOX12 BHLHA15 POU3F1 MYT1 CPT1B GEM EREG KAT6B TXNIP ULK1 ZNF552 FLG ZNF792 ZNF395 TSHZ1 KRBA2 GATA2 PDZD3 OTX1 PPARGC1A HOXC6 ABCA1 EGR1 SNAI3 CBX2 ZBTB10 NR0B2 ELL2 POU6F1 FOSL1 EFNA1 NR4A2 SEPSECS BCL6 AGO4 FGF19 CCDC88C JMY SP5 MXD4 CDKN1C ZNF512B CSRNP1 CDKN1A BTG2 CITED2 CITED4 HNF4G CHD6 AREG GLI2 AKAP12 SPRED3 H2AFJ SIX5 ANKRD1 ZNF488 ZNF763 EID2B TIGD3 KDM6B ZNF362 EDN1 FOXD2 TNFRSF1B CNKSR3 IRF2 AHRR MDFI KDM7A HBEGF ZNF233 CREB5 SRF ADCY4 PTGS2 ADCY5 FGD2 DNAJB5 BRD8 ATOH1 CDX2 GRHL3 CCK ACVR2B PNRC1 EPGN ZNF618 RHEBL1*
43583	ear development	3.58E-04	1.84E-02	9/387 2.3%	101/17777 0.5%	*FLG TMIE EDN1 TSHZ1 OSR1 OTX1 SOBP ATOH1 GLI2*
90068	positive regulation of cell cycle process	3.59E-04	1.84E-02	6/387 1.5%	44/17777 0.2%	*CDKN1A EDN1 EPGN CYP1A1 AHRR EREG*

9966	regulation of signal transduction	3.96E-04	2.01E-02	32/387 8.2%	773/17777 4.3%	*FLG ERRFI1 CSF2 RGS16 SERPINE1 SECTM1 TNFAIP3 FASLG KNDC1 FGD2 AKAP12 SPRED3 ERBB3 RASGEF1B TNFSF10 GPAT3 FBXO8 AGFG2 MAP3K3 EDN1 CCK EREG ITPKB EFNA1 EPGN BCL6 FGF19 DDIT4 CNKSR3 RAPGEF5 MDFI HBEGF*
1523	retinoid metabolic process	4.34E-04	2.03E-02	5/387 1.2%	30/17777 0.1%	*ALDH1A3 DHRS9 CYP1A1 AHRR BCO2*
6776	vitamin A metabolic process	4.34E-04	2.03E-02	5/387 1.2%	30/17777 0.1%	*ALDH1A3 DHRS9 CYP1A1 AHRR BCO2*
16101	diterpenoid metabolic process	4.34E-04	2.03E-02	5/387 1.2%	30/17777 0.1%	*ALDH1A3 DHRS9 CYP1A1 AHRR BCO2*
31323	regulation of cellular metabolic process	4.37E-04	2.03E-02	109/387 28.1%	3733/17777 20.9%	*ERRFI1 CSF2 ZBTB24 GUCA1B TNC RORC JADE1 IRS2 NR3C2 MYC SALL4 MYB LIPG DPF1 PDK4 ZNF565 HOXA5 AGFG2 PRKCG KMT5C MAMSTR ARID5B SOX12 BHLHA15 POU3F1 MYT1 CPT1B GEM EREG KAT6B TXNIP ULK1 ZNF552 ZNF792 ZNF395 TSHZ1 KRBA2 GATA2 PDZD3 OTX1 PPARGC1A HOXC6 ABCA1 EGR1 SNAI3 CBX2 ZBTB10 NR0B2 ELL2 POU6F1 FOSL1 EFNA1 NR4A2 SEPSECS BCL6 AGO4 FGF19 CCDC88C JMY SP5 MXD4 CDKN1C ZNF512B CSRNP1 CDKN1A BTG2 CITED2 CITED4 HNF4G CHD6 AREG GLI2 AKAP12 SPRED3 SIX5 ANKRD1 ZNF488 ZNF763 EID2B TIGD3 KDM6B ZNF362 EDN1 FOXD2 TNFRSF1B CNKSR3 IRF2 AHRR MDFI KDM7A HBEGF ZNF233 CREB5 SRF ADCY4 PTGS2 ADCY5 FGD2 DNAJB5 BRD8 ATOH1 CDX2 GRHL3 CCK ACVR2B PNRC1 EPGN ZNF618 RHEBL1*
23051	regulation of signaling process	4.43E-04	2.03E-02	32/387 8.2%	778/17777 4.3%	*FLG ERRFI1 CSF2 RGS16 SERPINE1 SECTM1 TNFAIP3 FASLG KNDC1 FGD2 AKAP12 SPRED3 ERBB3 RASGEF1B TNFSF10 GPAT3 FBXO8 AGFG2 MAP3K3 EDN1 CCK EREG ITPKB EFNA1 EPGN BCL6 FGF19 DDIT4 CNKSR3 RAPGEF5 MDFI HBEGF*

16711	flavonoid 3'-monooxygenase activity	4.73E-04	2.03E-02	2/387 0.5%	2/17777 0.0%	*CYP1A1 AHRR*
17143	insecticide metabolic process	4.73E-04	2.03E-02	2/387 0.5%	2/17777 0.0%	*CYP1A1 AHRR*
9635	response to herbicide	4.73E-04	2.03E-02	2/387 0.5%	2/17777 0.0%	*CYP1A1 AHRR*
19341	dibenzo-p-dioxin catabolic process	4.73E-04	2.03E-02	2/387 0.5%	2/17777 0.0%	*CYP1A1 AHRR*
60482	lobar bronchus development	4.73E-04	2.03E-02	2/387 0.5%	2/17777 0.0%	*WNT7B HOXA5*
60535	trachea cartilage morphogenesis	4.73E-04	2.03E-02	2/387 0.5%	2/17777 0.0%	*WNT7B HOXA5*
60536	cartilage morphogenesis	4.73E-04	2.03E-02	2/387 0.5%	2/17777 0.0%	*WNT7B HOXA5*
9404	toxin metabolic process	5.29E-04	2.22E-02	3/387 0.7%	8/17777 0.0%	*CYP1A1 CYP1B1 AHRR*
60638	mesenchymal-epithelial cell signaling	5.29E-04	2.22E-02	3/387 0.7%	8/17777 0.0%	*FLG TNC HOXA5*
42221	response to chemical stimulus	5.72E-04	2.37E-02	51/387 13.1%	1466/17777 8.2%	*CDKN1A BTG2 CXCL8 CITED2 SERPINE1 TNC IRS2 FASLG SLC7A11 AQP3 CXCL2 MYC LIPG CYP1B1 PRSS8 CHAC1 PDIA2 EDN1 OSR1 EREG TGFBR3 ALDH1A3 DDIT4 TXNIP AHRR PDE5A SRF ADCY4 STC1 PTGS2 NDRG1 ADCY5 PDZD3 ERBB3 DNAJB5 EGR1 FZD4 CCL20 WNT7B PTCH1 HTR1D CCK SUOX NR0B2 ACVR2B FOSL1 NR4A2 GATM VNN1 CYP1A1 CALM2*
7050	cell cycle arrest	5.87E-04	2.41E-02	9/387 2.3%	108/17777 0.6%	*CDKN1C CDKN1A CXCL8 SESN3 MYC SESN1 TP53INP1 JMY HBP1*
34754	cellular hormone metabolic process	6.21E-04	2.51E-02	7/387 1.8%	67/17777 0.3%	*ALDH1A3 DHRS9 HSD3B1 CYP1A1 CYP1B1 AHRR BCO2*

51094	positive regulation of developmental process	6.36E-04	2.54E-02	18/387 4.6%	347/17777 1.9%	*FLG CSF2 PKDCC CDX2 WNT7B SRF SERPINE1 CCK GATA2 PTGS2 ACVR2B GLI2 ITPKB VNN1 MYC ZNF488 HOXA5 ATOH1*
35466	regulation of signaling pathway	6.44E-04	2.55E-02	38/387 9.8%	1003/17777 5.6%	*FLG CDKN1C GPSM1 ERRFI1 CSF2 CITED2 RGS16 SECTM1 TNFAIP3 FASLG CBLB KNDC1 GLI2 FGD2 AKAP12 SPRED3 ERBB3 RASGEF1B TNFSF10 GPAT3 FBXO8 AGFG2 MAP3K3 EDN1 PTCH1 CCK ACVR2B EREG ITPKB EFNA1 EPGN BCL6 FGF19 CNKSR3 ULK1 RAPGEF5 MDFI HBEGF*
8285	negative regulation of cell proliferation	6.86E-04	2.68E-02	19/387 4.9%	379/17777 2.1%	*CDKN1C CD274 CDKN1A BTG2 CXCL8 PTCH1 CBLB DLEC1 PTGS2 EREG FOSL1 TGFBR3 BCL6 SIX5 SESN1 FRK PTGES MXD4 BNIPL*
3002	regionalization	7.54E-04	2.91E-02	13/387 3.3%	211/17777 1.1%	*BTG2 EDN1 CDX2 WNT7B TSHZ1 PTCH1 ACVR2B GLI2 LFNG OTX1 HOXA5 MDFI HOXC6*
17085	response to insecticide	7.80E-04	2.94E-02	3/387 0.7%	9/17777 0.0%	*NR4A2 CYP1A1 AHRR*
18195	peptidyl-arginine modification	7.80E-04	2.94E-02	3/387 0.7%	9/17777 0.0%	*PADI3 PADI2 PADI1*
10035	response to inorganic substance	7.86E-04	2.94E-02	14/387 3.6%	239/17777 1.3%	*CDKN1A PTCH1 SERPINE1 TNC PTGS2 AQP3 NDRG1 FOSL1 NR4A2 GATM CYP1A1 TXNIP AHRR CALM2*
80090	regulation of primary metabolic process	8.58E-04	3.18E-02	103/387 26.6%	3552/17777 19.9%	*ERRFI1 CSF2 ZBTB24 SERPINE1 GUCA1B TNC RORC JADE1 IRS2 CBLB NR3C2 MYC SALL4 MYB DPF1 ZNF565 HOXA5 AGFG2 PRKCG KMT5C MAMSTR ARID5B SOX12 BHLHA15 POU3F1 MYT1 CPT1B GEM EREG KAT6B TXNIP ZNF552 ZNF792 ZNF395 TSHZ1 KRBA2 GATA2 PDZD3 OTX1 PPARGC1A HOXC6 ABCA1 EGR1 SNAI3 CBX2 ZBTB10 NR0B2 ELL2 POU6F1 FOSL1 NR4A2 SEPSECS BCL6 AGO4 FGF19 CCDC88C JMY SP5 MXD4 CDKN1C ZNF512B CSRNP1 BTG2 CITED2 CITED4 HNF4G CHD6 AREG GLI2 AKAP12 SIX5 ANKRD1 ZNF488 ZNF763 EID2B TIGD3 KDM6B ZNF362 EDN1 FOXD2 TNFRSF1B CNKSR3 IRF2 AHRR MDFI KDM7A ZNF233*

						CREB5 SRF ADCY4 PTGS2 ADCY5 FGD2 DNAJB5 BRD8 ATOH1 CDX2 GRHL3 CCK ACVR2B PNRC1 ZNF618 RHEBL1
45787	positive regulation of cell cycle	8.81E-04	3.23E-02	7/387 1.8%	71/17777 0.3%	*FOSL1 EDN1 EPGN CITED2 CYP1A1 AHRR EREG*
4879	ligand-dependent nuclear receptor activity	8.95E-04	3.24E-02	6/387 1.5%	52/17777 0.2%	*NR4A2 RORC HNF4G BRD8 NR0B2 NR3C2*
4713	protein tyrosine kinase activity	9.41E-04	3.37E-02	10/387 2.5%	139/17777 0.7%	*FLG EPHA4 EFNA3 ERBB3 TXK TESK2 JAK3 EFNA4 FRK FGFR2*
31667	response to nutrient levels	9.61E-04	3.41E-02	14/387 3.6%	244/17777 1.3%	*FZD4 WNT7B PTCH1 STC1 CCK PTGS2 SUOX AQP3 GATM NUAK2 LIPG CYP1A1 AHRR PPARGC1A*
10469	regulation of receptor activity	9.87E-04	3.44E-02	4/387 1.0%	21/17777 0.1%	*ERRFI1 EPGN SERPINE1 EREG*
42472	inner ear morphogenesis	9.91E-04	3.44E-02	6/387 1.5%	53/17777 0.2%	*FLG TMIE OTX1 SOBP ATOH1 GLI2*
48729	tissue morphogenesis	1.03E-03	3.55E-02	15/387 3.8%	274/17777 1.5%	*FLG ERRFI1 EDN1 WNT7B SRF OSR1 PTCH1 TNC GLI2 TGFBR3 ALDH1A3 MYC TRIM15 HOXA5 FREM2*
48522	positive regulation of cellular process	1.05E-03	3.57E-02	64/387 16.5%	2003/17777 11.2%	*CDKN1C CSRNP1 CDKN1A VIPR1 CSF2 CITED2 SERPINE1 CITED4 SECTM1 IRS2 FASLG AREG GLI2 AKAP12 MYC SALL4 DPF1 TNFSF10 ZNF488 PHLDA1 HOXA5 PRKCG EDN1 BHLHA15 EREG ITPKB ALDH1A3 KAT6B TXNIP AHRR PDE5A HBEGF CHST3 CREB5 FLG PKDCC SRF GATA2 PTGS2 FGD2 ERBB3 TP53INP1 PPARGC1A ATOH1 ABCA1 EGR1 MAP3K3 CDX2 WNT7B CIDEB GRHL3 CCK NR0B2 ACVR2B FOSL1 NR4A2 EPGN VNN1 BCL6 FGF19 JMY CYP1A1 FGFR2 BNIPL*
46685	response to arsenic	1.10E-03	3.68E-02	3/387 0.7%	10/17777 0.0%	*CDKN1A CYP1A1 AHRR*

48732	gland development	1.10E-03	3.68E-02	12/387 3.1%	193/17777 1.0%	*FLG ALDH1A3 ERBB3 CITED2 WNT7B PTCH1 TNC IRS2 GATA2 HOXA5 GLI2 APLN*
45595	regulation of cell differentiation	1.11E-03	3.68E-02	24/387 6.2%	553/17777 3.1%	*FLG ABCA1 ERRFI1 EDN1 CSF2 PKDCC CDX2 WNT7B SRF OSR1 CCK AQP3 ACVR2B GLI2 EREG ITPKB EFNA1 VNN1 BCL6 ULK1 ZNF488 HIST1H4D HOXA5 ATOH1*
10468	regulation of gene expression	1.18E-03	3.88E-02	87/387 22.4%	2928/17777 16.4%	*CDKN1C ZNF512B CSRNP1 BTG2 CSF2 ZBTB24 CITED2 SERPINE1 CITED4 RORC HNF4G JADE1 CHD6 GLI2 NR3C2 H2AFJ SIX5 MYC SALL4 MYB DPF1 ANKRD1 ZNF488 ZNF565 ZNF763 HOXA5 EID2B TIGD3 KDM6B ZNF362 FOXD2 KMT5C MAMSTR OSR1 ARID5B SOX12 BHLHA15 TNFRSF1B POU3F1 MYT1 GEM EREG KAT6B IRF2 TXNIP AHRR MDFI KDM7A ZNF233 ZNF552 CREB5 FLG ZNF792 ZNF395 SRF TSHZ1 KRBA2 GATA2 DNAJB5 BRD8 OTX1 PPARGC1A ATOH1 HOXC6 EGR1 CDX2 SNAI3 CBX2 ZBTB10 GRHL3 CCK NR0B2 ELL2 ACVR2B PNRC1 POU6F1 FOSL1 NR4A2 SEPSECS BCL6 ZNF618 AGO4 CCDC88C JMY SP5 RHEBL1 MXD4*
48565	digestive tract development	1.21E-03	3.90E-02	6/387 1.5%	55/17777 0.3%	*PKDCC CYP1A1 AHRR ACVR2B HOXA5 GLI2*
7584	response to nutrient	1.21E-03	3.90E-02	11/387 2.8%	169/17777 0.9%	*GATM FZD4 WNT7B PTCH1 LIPG CYP1A1 AHRR STC1 PTGS2 SUOX AQP3*
48469	cell maturation	1.32E-03	4.08E-02	7/387 1.8%	76/17777 0.4%	*FLG CDKN1C NR4A2 BHLHA15 GATA2 HOXA5 EREG*
6721	terpenoid metabolic process	1.32E-03	4.08E-02	5/387 1.2%	38/17777 0.2%	*ALDH1A3 DHRS9 CYP1A1 AHRR BCO2*
21700	developmental maturation	1.34E-03	4.08E-02	8/387 2.0%	98/17777 0.5%	*FLG CDKN1C NR4A2 BHLHA15 GATA2 PTGS2 HOXA5 EREG*

7399	nervous system development	1.39E-03	4.08E-02	41/387 10.5%	1155/17777 6.4%	*FLG CDKN1C GPSM1 BTG2 SEMA7A PTPRR GAL3ST1 CITED2 SRF CHD6 IRS2 GATA2 GLI2 ERBB3 DPF1 SLITRK6 OTX1 ZNF488 ATOH1 EPHA4 FZD2 UNC5B FZD4 WNT7B PTCH1 LRRN4 CCK POU3F1 MYT1 POU6F1 EFNA1 NR4A2 SLC7A5 ALDH1A3 FGF19 ULK1 PDE5A TAGLN3 RAPGEF5 KDM7A CHST3*
33829	O-fucosylpeptide 3-beta-N-acetylglucosaminyltransferase activity	1.40E-03	4.08E-02	2/387 0.5%	3/17777 0.0%	*LFNG MFNG*
42178	xenobiotic catabolic process	1.40E-03	4.08E-02	2/387 0.5%	3/17777 0.0%	*CYP1A1 AHRR*
10041	response to iron(III) ion	1.40E-03	4.08E-02	2/387 0.5%	3/17777 0.0%	*CYP1A1 AHRR*
18894	dibenzo-p-dioxin metabolic process	1.40E-03	4.08E-02	2/387 0.5%	3/17777 0.0%	*CYP1A1 AHRR*
60534	trachea cartilage development	1.40E-03	4.08E-02	2/387 0.5%	3/17777 0.0%	*WNT7B HOXA5*
5294	neutral L-amino acid secondary active transmembrane transporter activity	1.40E-03	4.08E-02	2/387 0.5%	3/17777 0.0%	*SLC6A9 SLC7A11*
72080	nephron tubule development	1.40E-03	4.08E-02	2/387 0.5%	3/17777 0.0%	*WNT7B OSR1*
1533	cornified envelope	1.41E-03	4.08E-02	4/387 1.0%	23/17777 0.1%	*FLG SPRR1A IVL SPRR1B*
16813	hydrolase activity, acting on carbon-nitrogen (but not peptide) bonds, in linear amidines	1.48E-03	4.18E-02	3/387 0.7%	11/17777 0.0%	*PADI3 PADI2 PADI1*
60428	lung epithelium development	1.48E-03	4.18E-02	3/387 0.7%	11/17777 0.0%	*ERRFI1 WNT7B HOXA5*

55093	response to hyperoxia	1.48E-03	4.18E-02	3/387 0.7%	11/17777 0.0%	*CDKN1A CYP1A1 AHRR*
122	negative regulation of transcription from RNA polymerase II promoter	1.58E-03	4.41E-02	15/387 3.8%	286/17777 1.6%	*CDKN1C KDM6B EGR1 CDX2 CITED2 CBX2 NR0B2 GLI2 BCL6 DNAJB5 SALL4 IRF2 TXNIP MDFI MXD4*
4672	protein kinase activity	1.59E-03	4.41E-02	24/387 6.2%	568/17777 3.1%	*PRKCG FLG EPHA4 MAP3K3 CDKN1A CDKL5 PKDCC TXK OSR1 TESK2 CCK MYT1 EFNA4 ACVR2B TGFBR3 EPGN EFNA3 NUAK2 ERBB3 PDK4 ULK1 JAK3 FRK FGFR2*
10556	regulation of macromolecule biosynthetic process	1.62E-03	4.46E-02	85/387 21.9%	2877/17777 16.1%	*CDKN1C ZNF512B CSRNP1 BTG2 CSF2 ZBTB24 CITED2 CITED4 RORC HNF4G JADE1 CHD6 IRS2 AREG GLI2 NR3C2 SIX5 MYC SALL4 MYB DPF1 ANKRD1 ZNF488 ZNF565 ZNF763 HOXA5 EID2B TIGD3 KDM6B ZNF362 EDN1 FOXD2 KMT5C MAMSTR ARID5B SOX12 BHLHA15 POU3F1 MYT1 GEM EREG KAT6B IRF2 TXNIP AHRR MDFI KDM7A HBEGF ZNF233 ZNF552 CREB5 ZNF792 ZNF395 SRF TSHZ1 KRBA2 GATA2 DNAJB5 BRD8 OTX1 PPARGC1A ATOH1 HOXC6 EGR1 CDX2 SNAI3 CBX2 ZBTB10 GRHL3 CCK NR0B2 ELL2 ACVR2B PNRC1 POU6F1 FOSL1 NR4A2 SEPSECS BCL6 ZNF618 AGO4 JMY SP5 RHEBL1 MXD4*
42058	regulation of epidermal growth factor receptor signaling pathway	1.66E-03	4.48E-02	4/387 1.0%	24/17777 0.1%	*ERRFI1 EPGN FASLG EREG*
6775	fat-soluble vitamin metabolic process	1.68E-03	4.48E-02	5/387 1.2%	40/17777 0.2%	*ALDH1A3 DHRS9 CYP1A1 AHRR BCO2*
31970	organelle envelope lumen	1.68E-03	4.48E-02	5/387 1.2%	40/17777 0.2%	*GATM HSD3B1 TXNIP SUOX PTGES*
45786	negative regulation of cell cycle	1.68E-03	4.48E-02	11/387 2.8%	176/17777 0.9%	*CDKN1C CDKN1A CXCL8 SESN3 BCL6 MYC SESN1 TP53INP1 JMY HBP1 GEM*

30182	neuron differentiation	1.74E-03	4.60E-02	20/387 5.1%	442/17777 2.4%	*FLG CDKN1C EPHA4 BTG2 PTPRR FZD2 UNC5B FZD4 WNT7B SRF CCK GATA2 GLI2 EFNA1 NR4A2 ERBB3 SLITRK6 ULK1 ATOH1 CHST3*
19199	transmembrane receptor protein kinase activity	1.78E-03	4.68E-02	7/387 1.8%	80/17777 0.4%	*FLG TGFBR3 EPHA4 EFNA3 ERBB3 EFNA4 FGFR2*
51173	positive regulation of nitrogen compound metabolic process	1.91E-03	4.98E-02	27/387 6.9%	677/17777 3.8%	*CSRNP1 CSF2 CITED2 SRF CITED4 GATA2 PTGS2 AREG GLI2 FGD2 AKAP12 MYC SALL4 PPARGC1A HOXA5 ATOH1 PRKCG ABCA1 EGR1 EDN1 GRHL3 BHLHA15 EREG FOSL1 NR4A2 KAT6B CREB5*
42981	regulation of apoptosis	1.93E-03	5.00E-02	32/387 8.2%	851/17777 4.7%	*FLG CDKN1A BTG2 CSF2 TNFRSF6B CITED2 SERPINE1 TNFAIP3 IRS2 FASLG PTGS2 FGD2 NUAK2 ERBB3 TP53INP1 DPF1 TNFSF10 PHLDA1 HOXA5 OSR1 CIDEB CCK FOSL1 NR4A2 ALDH1A3 VNN1 PROC BCL6 JMY TXNIP PDE5A BNIPL*
45742	positive regulation of epidermal growth factor receptor signaling pathway	1.95E-03	5.00E-02	3/387 0.7%	12/17777 0.0%	*EPGN FASLG EREG*

[a] *p*-value in hypergeometric test after correction

[b] The denominator represents the total number of genes with GO annotation and the numerator represents the number of genes belonging to each GO term

[c] The denominator represents the number of reference genes with GO annotation and the numerator represents the number of references genes annotated in the listed GO terms

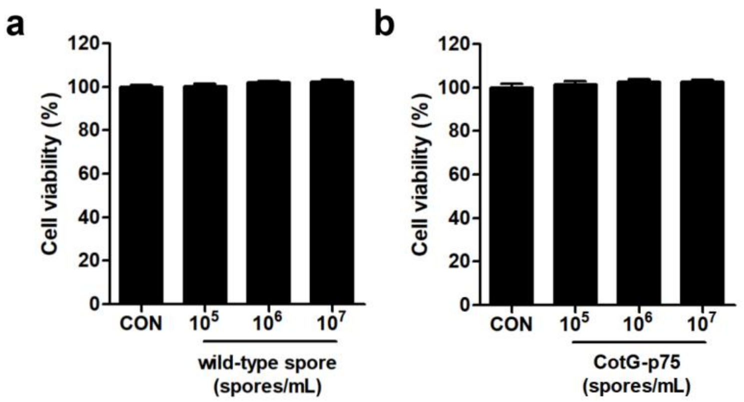

Figure S1. Effect of wild-type spores (a) and CotG-p75 (b) on cell viability of intestinal epithelial HT-29 cells. Cell viability was determined after 24 h incubation with each spore. Each column represents the mean ± standard deviation (n = 3) with respect to 100% control. Statistical analysis was performed by a one-way ANOVA and Tukey-Kramer post-hoc analysis.

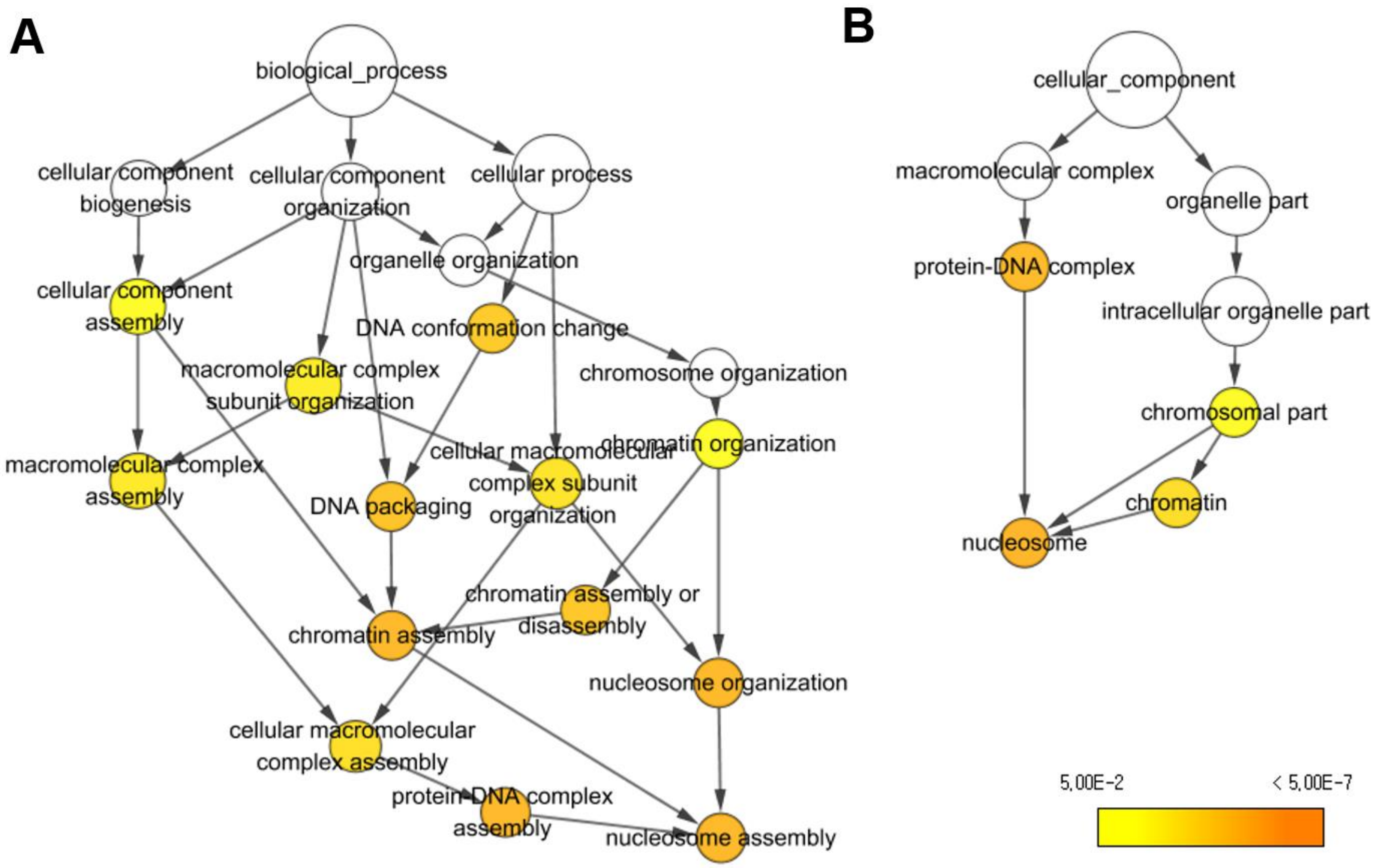

Figure S2. Illustration of Gene ontology (GO) term networks of differentially expressed genes between wild-type spore-stimulated HT-29 cells and control. The networks were generated by Cytoscape BiNGO. All significantly enriched GO terms in biological process (A) and cellular component (B) are presented. Circle size represents GO hierarchy. Yellow shade represents enrichment level. The threshold of hypergeometric distribution of the functional annotation was set at corrected *p*-value < 0.005

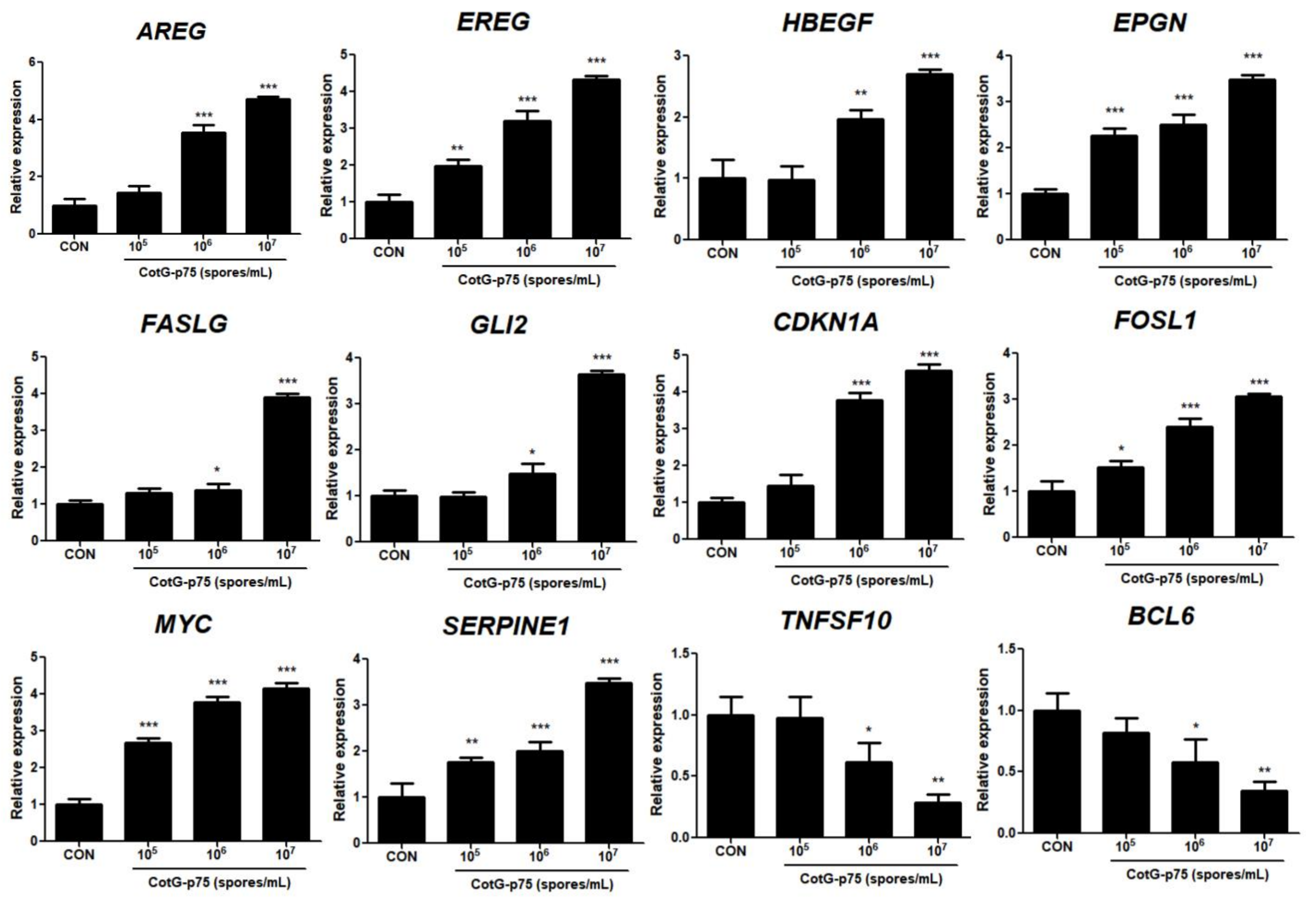

Figure S3. The mRNA expression of the genes involved in cell survival and proliferation from HT-29 stimulated by different doses of CotG-p75. HT-29 cells were incubated with 10^5, 10^6, and 10^7 spores/mL of CotG-p75 for 3 h. The relative expression level was determined by real-time quantitative PCR. RT-qPCR data presented as mean ± standard deviation (n = 3). Statistical analysis was performed by a one-way ANOVA and Tukey-Kramer post-hoc analysis. Asterisks (*) indicate a significance difference from the control (* $p < 0.05$, ** $p < 0.01$, *** $p < 0.001$).

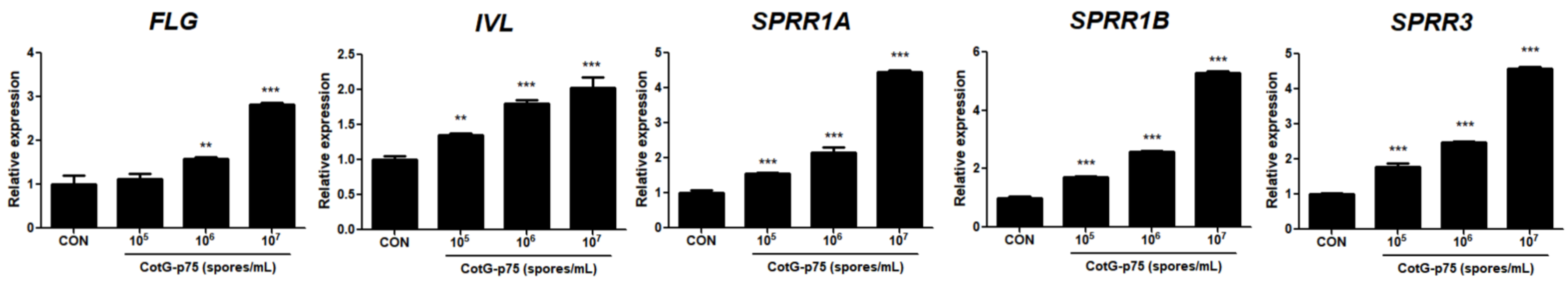

Figure S4. The mRNA expression of the genes involved in epithelium development and differentiation from HT-29 stimulated by different doses of CotG-p75. HT-29 cells were incubated with 10^5, 10^6, and 10^7 spores/mL of CotG-p75 for 3 h. The relative expression level was determined by real-time quantitative PCR. RT-qPCR data presented as mean ± standard deviation (n = 3). Statistical analysis was performed by a one-way ANOVA and Tukey-Kramer post-hoc analysis. Asterisks (*) indicate a significance difference from the control (* $p < 0.05$, ** $p < 0.01$, *** $p < 0.001$).

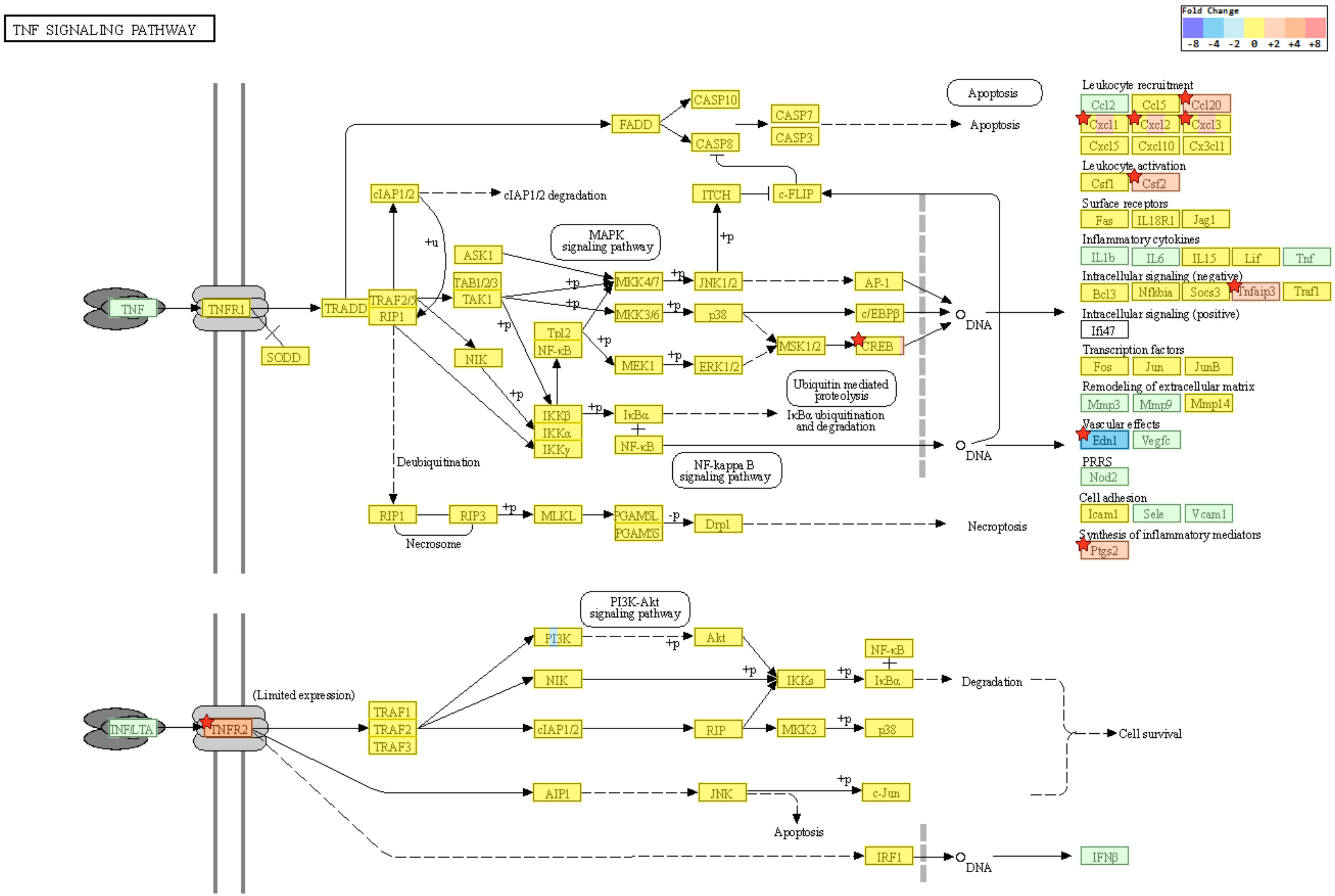

Figure S5. TNF signaling pathway in KEGG pathway analysis. Red and blue colors represent up-regulated and down-regulated DEGs, respectively.

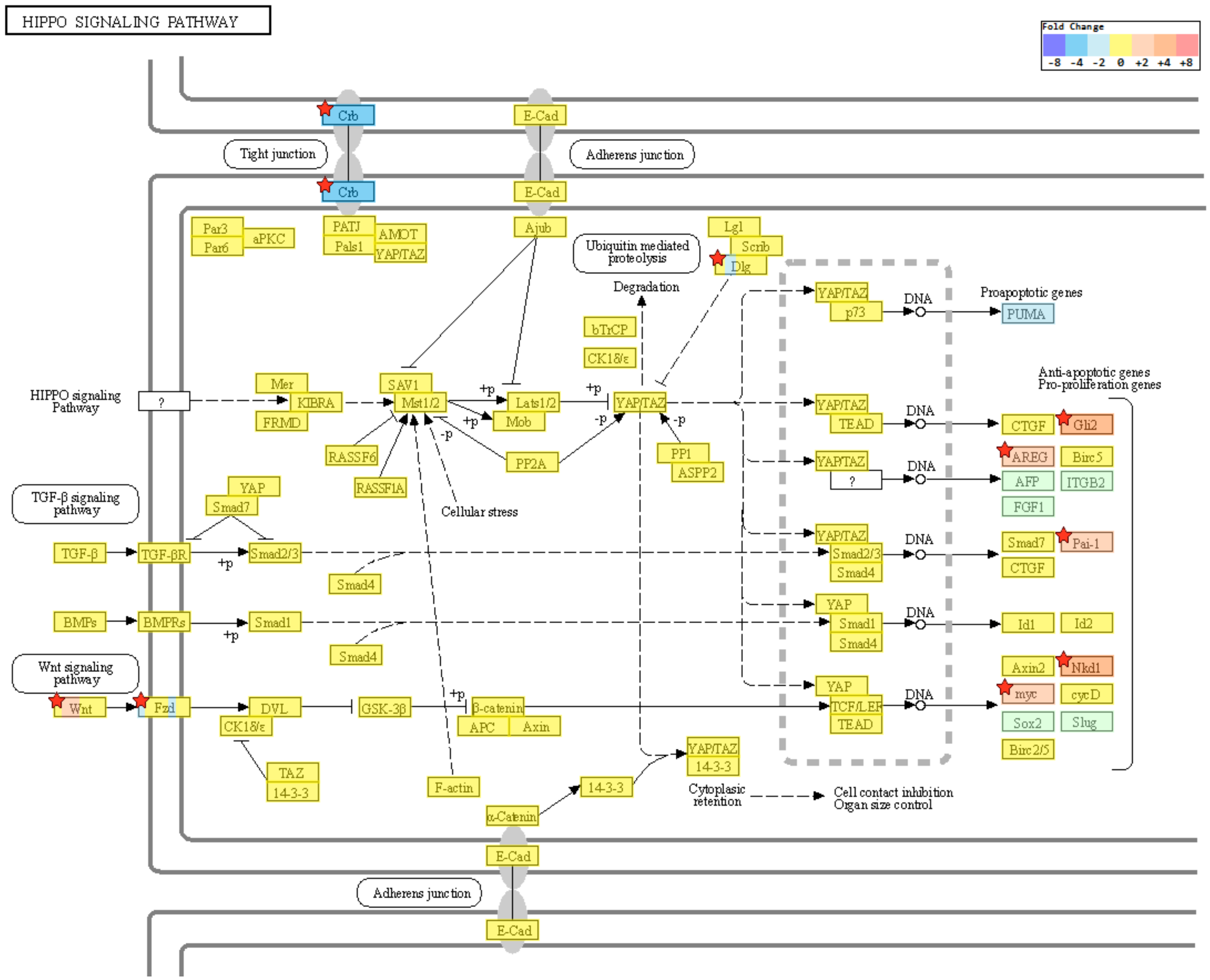

Figure S6. Hippo signaling pathway in KEGG pathway analysis. Red and blue colors represent up-regulated and down-regulated DEGs, respectively.
